# Characterization of molecular subtypes based on chromatin regulators and identification of the role of NPAS2 in lung adenocarcinoma

**DOI:** 10.1186/s13148-023-01486-w

**Published:** 2023-04-29

**Authors:** Yongbiao Huang, Lingyan Xiao, Motuma Yigezu Daba, Duo Xu, Yuan Wang, Long Li, Qian Li, Bo Liu, Wan Qin, Huixian Zhang, Xianglin Yuan

**Affiliations:** 1 Department of Oncology, Tongji Hospital, Tongji Medical College, Huazhong University of Science and Technology, Wuhan, China; 2 Department of Pathophysiology, School of Basic Medicine, Tongji Medical College, Huazhong University of Science and Technology, Wuhan, China; 3 Department of Medical Oncology, The First Affiliated Hospital of Zhengzhou University, Zhengzhou, China

**Keywords:** Lung adenocarcinoma, Chromatin regulator, Molecular subtype, Tumor microenvironment, *NPAS2*

## Abstract

**Background:**

Chromatin regulators (CRs) are critical epigenetic modifiers and have been reported to play critical roles during the progression of various tumors, but their role in lung adenocarcinoma (LUAD) has not been comprehensively studied.

**Methods:**

Differential expression and univariate Cox regression analyses were conducted to identify the prognostic CRs. Consensus clustering was applied to classify the subtypes of LUAD based on prognostic CRs. LASSO-multivariate Cox regression method was used for construction of a prognostic signature and development of chromatin regulator-related gene index (CRGI). The capacity of CRGI to distinguish survival was evaluated via Kaplan–Meier method in multiple datasets. Relationship between CRGI and tumor microenvironment (TME) was evaluated. Additionally, clinical variables and CRGI were incorporated to create a nomogram. The role of the prognostic gene *NPAS2* in LUAD was elucidated via clinical samples validation and a series of in vitro and in vivo experiments.

**Results:**

Two subtypes of LUAD were classified based on 46 prognostic CRs via consensus clustering which had significantly different survival and TME. A prognostic signature consisting of six CRs (*MOCS*, *PBK*, *CBX3*, *A1CF*, *NPAS2*, and *CTCFL*) was developed and proved to be an effective survival predictor in multiple independent datasets. The prognostic signature was also demonstrated to be an indicator of TME and sensitivity to immunotherapy and chemotherapy. The nomogram was suggested to be a simple tool that can predict survival accurately. Clinical samples show that *NPAS2* is highly expressed in LUAD tissues, and in vitro and in vivo experiments demonstrated that inhibition of *NPAS2* impeded malignant progression of LUAD cells.

**Conclusions:**

Our study comprehensively unveiled the functions of CRs in LUAD, developed a classifier to predict survival and response to treatments, and suggested that *NPAS2* promoted LUAD progression for the first time.

**Supplementary Information:**

The online version contains supplementary material available at 10.1186/s13148-023-01486-w.

## Introduction

Lung cancer is one of the most frequently diagnosed cancers which is made up of diverse histological subtypes, with lung adenocarcinoma representing approximately 54.7% of all lung cancer cases [1]. Despite the encouraging progresses achieved in screening and treatment, the prognosis of patient with LUAD is not optimistic as only 26.4% of LUAD individuals can survive 5 years or longer [1]. Application of predictive biomarkers can aid in stratifying patients with varying degrees of mortality risk. Poor survival of LUAD makes it necessary to discover more robust biomarkers to achieve individual treatment.

CRs consisting of DNA methylators, histone modifiers, and chromatin remodelers can control epigenetic alteration which is one of the cancer hallmarks [2]. CRs are crucial modulators in a variety of biological processes such as energy metabolism and activation of T cells [3, 4]. Dysregulation of CRs affects chromatin structure and expression of cancer-related genes, leading to changes in biological processes and malignant behaviors [2, 5]. Perturbation of CRs is common in various cancers, and mutation or aberrant expression of CRs was associated with outcome of malignancies [6]. For example, *DNMT1* which belongs to the family of DNA methyltransferases not only regulated stemness and tumorigenicity of liver cancer and breast cancer, but also predicted poor prognosis of cervical cancer and breast cancer [7–9]. Another study has revealed that CRs served as a predictor of survival and therapeutic response in bladder cancer [10]. The above studies suggest that CRs possess great potential to be reliable predictors of prognosis.

In this study, we comprehensively analyzed expression of CRs in LUAD and identified the prognostic CRs to characterize molecular subtypes of LUAD. Additionally, we developed a prognostic predictor based on prognostic CRs to predict survival and TME for LUAD individuals. We also investigated the role of *NPAS2* in progression of LUAD via a series of in vitro and in vivo experiments.

## Materials and methods

### Identification of prognostic CRs

Transcriptome, methylation data, and genome files of the LUAD samples were collected from The Cancer Genome Atlas (TCGA) database (https://gdc-portal.nci.nih.gov/). Chromatin regulators (CRs) were collected from previous studies [2] (Additional file 1: Table S1). After removal of genes with low expression and data normalization, the differentially expressed CRs were identified by “edgeR” package based on the screening criteria of adjusted *p* value (FDR) < 0.05 and |log2 fold change (FC)|> 1.0. The differentially expressed CRs which had statistically significant effect on survival in in univariate Cox regression analysis were considered prognostic CRs. Mutation landscape and copy number variations (CNV) of the CRs were assessed based on genome data of the TCGA-LUAD cohort.

### Subtype characterization of LUAD

Expression data of GSE37745, GSE31210, and GSE50081 were collected from the Gene Expression Omnibus (GEO) database (https://www.ncbi.nlm.nih.gov/geo/). After those without complete clinicopathological information or followed up less than a month were removed, LUAD individuals from TCGA, GSE37745, and GSE31210 were merged into a large LUAD cohort which consisted of 800 samples. “SVA” package which used the ComBat function to remove both known batch effects and other potential latent sources of variation was applied in our study to remove the batch effect between the LUAD datasets [11]. We summarized the clinicopathological variables of all the LUAD samples in Additional file 2: Table S2. The consensus clustering algorithm, an unsupervised clustering method, is able to detect potential groups of a dataset based on intrinsic characteristics, making it a popular data mining technique in cancer research [12]. In this study, consensus clustering was executed by “ConsensusClusterPlus” package to characterize the subtypes of LUAD based on expression of prognostic CRs [13]. Principal component analysis (PCA) was carried out to confirm the capability of the prognostic CRs in distinguishing the clusters identified in consensus clustering. Kaplan–Meier survival curves were created to test the prognostic value of the CR subtypes.

### The relationship of CR-related subtypes with methylation and TME

The chromatin regulators control epigenetic alteration including DNA methylation. The CpG sites with significantly different methylation levels between the CR-related subtypes were screened according to *β* value using “limma” package, and the cutoff criteria were FDR < 0.05. TME exerts tremendous influence in tumorigenesis and is associated with prognosis of patients. The TME is mainly composed of stromal cells and immune cells. The abundance of immune cells, stromal cells, and tumor cells was quantified via Estimation of STromal and Immune cells in MAlignant Tumors using Expression data (ESTIMATE) method by “estimate” package. There are various subtypes of immune cells which fulfill different functions. Cell-type Identification By Estimating Relative Subsets Of RNA Transcripts (CIBERSORT) method was utilized to calculate the abundance of the major subtypes of immune cells. Gene set variation analysis (GSVA) was executed to investigate which pathways differed between the two subtypes with FDR < 0.05 as screening criteria.

### Construction of a CR-associated signature

Further selection of prognostic CRs was carried out by Least Absolute Shrinkage and Selection Operator (LASSO) Cox regression analysis. Then, multivariate Cox regression analysis was used to construct a signature based on the CRs selected in LASSO method. CRGI was calculated via mRNA levels of CRs and corresponding coefficients identified in the multivariate Cox regression analysis, and the median value of CRGI was set as the division criterion to classify patients into high- and low-CRGI groups. The relationship between the subtypes and CRGI groups was presented by Sankey diagram.

### Validation of the prognostic signature

PCA was conducted in the TCGA cohort to confirm the capability of the CRs in the signature to discriminate between those with different CRGIs. The survival difference between the high- and low-CRGI groups was displayed by Kaplan–Meier survival curves. We also calculated the survival percentage of the high- and low-CRGI groups and the distribution of CRGI between survivors and nonsurvivors. The exact effect of CRGI on survival was assessed by univariate and multivariate Cox regression analyses. The predictive capability of CRGI was inferred by receiver operating characteristic (ROC) curves. To further validate the prognostic value of CRGI, PCA and Kaplan–Meier survival analysis were carried out in GSE37745 and GSE31210.

### TME patterns of the high- and low-CRGI groups

As has been described above, ESTIMATE and CIBERSORT algorithms were run to investigate the patterns of TME in the high- and low-CRGI groups. Additionally, the correlation between CRGI and immune cells was also evaluated. Expression of immune checkpoint genes is associated with function of immune cells and therapeutic response of immunotherapy. Thus, the relationship between the expression of immune checkpoint genes and CRGI was assessed.

### Construction and assessment of a nomogram

Considering that clinicopathological variables are contributing factors of survival, we developed a nomogram consisting of clinicopathological parameters and CRGI to improve the predictive precision of CRGI. Clinicians can use the points obtained from the nomogram to predict survival probability of patients. Calibration curves and ROC curves were generated, and C-index and area under the curve (AUC) were calculated to evaluate predictive capability of the nomogram, CRGI, and other clinical variables.

### Relationship between CRGI and sensitivity to treatment

The sensitivity of the high- and low-CRGI groups to immunotherapy and chemotherapy which are effective treatments for LUAD individuals was also predicted. Response of the high- and low-CRGI groups to immunotherapy and chemotherapy was compared to figure out whether CRGI could be a tool for stratification of therapeutic benefits. Immunophenoscore (IPS) was developed based on expression of immune-related genes, which can predict response to CTLA-4 and PD-1 antibodies [14]. IPS was positively associated with efficacy to immunotherapy [14]. IPS of 20 solid cancers in the TCGA database is provided in The Cancer Immunome Atlas (TCIA) (https://tcia.at/home). We collected IPS of the TCGA-LUAD cohort and compared IPS between the high- and low-CRGI groups. Chemotherapeutic agents such as cisplatin and paclitaxel can achieve satisfactory efficacy in patients with LUAD. Half-maximal inhibitory concentration (IC50) of chemotherapeutic agents was acquired using “pRRophetic” package to evaluate therapeutic benefits of chemotherapy in the high- and low-CRGI groups.

### Clinical specimens and immunohistochemistry (IHC)

We also collected lung adenocarcinoma tissues and paired normal lung tissues of 11 patients from Wuhan Tongji Hospital for IHC analysis. According to the manufacturer’s instructions, immunohistochemical staining of NPAS2 was conducted. The specific primary antibodies anti-NPAS2 (Invitrogen, PA5-98824) was used for IHC. The IHC scores of each patient were calculated to evaluate the NPAS2 protein expression levels based on the staining intensity and quantity.

### Cell culture and siRNA transfection

The human lung adenocarcinoma cell lines including PC-9, A549, HCC827, H1975 and the normal lung epithelial cell line BEAS-2b were stored in the oncology laboratory of Tongji Hospital, Wuhan, China. All these cells were grown in RPMI-1640 medium containing 10% FBS (Gibco, USA) and maintained in the 37 °C incubator with 5% CO_2_. The siRNA oligonucleotides (negative control siRNA and NPAS2 siRNA) were synthesized by GENERAL BIOL (Anhui, China), and siRNA transfection was carried out with Lipofectamine 3000 (Invitrogen, USA). The siRNA sequences were: si-NC, 5′-UUCUCCGAACGUGUCACGUTT-3′ (Sense) and 5′-ACGUGACACGUUCGGAGAATT-3′ (Antisense); si-NPAS2#1, 5′-CCUCAGCACUAAAGGACAATT-3′ (Sense) and 5′-UUGUCCUUUAGUGCUGAGGTT-3′ (Antisense); si-NPAS2#2, 5′-CCACCAAGCUGAUGGCAGATT-3′ (Sense) and 5′-UCUGCCAUCAGCUUGGUGGTT-3′ (Antisense).

### Quantitative real-time PCR (qRT-PCR)

Total cell RNA was isolated using TRIzol reagent (TaKaRa, Japan) and transcribed to cDNA using the reverse transcription reagents (Vazyme, China). Then, the qRT-PCR was conducted with a Real-Time PCR System (Applied Biosystems 7900HT, USA). The relative expression levels of *NPAS2* were calculated using the 2^−ΔΔCT^ method. The primer sequences were listed as follows: NPAS2-forward, 5′-CACAGAGCACCTCCAATCATAG‑3′ and NPAS2-reverse, 5′-GTAGCAACACGACTTCCCTT‑3′; GAPDH-forward, 5′-GACAGTCAGCCGCATCTTCT‑3′ and GAPDH-reverse, 5′-GCGCCCAATACGACCAAATC‑3′.

### Western blot

Western blot assay was conducted as previously described [15]. Briefly, the extracted protein samples were separated by SDS-PAGE and transferred to PVDF membranes. Next, the membranes were blocked using 5% nonfat milk and incubated with specific primary antibodies (GAPDH, 1:5000, 10494-1-AP, Proteintech and NPAS2, 1:1000, PA5-98824, Invitrogen). After incubation overnight at 4 °C, the membranes were washed and incubated with secondary antibodies (1:5000, SA00001-2, Proteintech). Finally, the protein bands were detected with the ECL reagent by the G: BOX Chemi X system (Syngene, UK).

### CCK8 assay

Two thousand to three thousand cells/well were plated into 96-well plates. After adherent, the cells were incubated with 100 μl medium containing 10% CCK8 (MedChem Express, USA) each well for 1–2 h at 37 °C. The absorbance (450 nm wavelength) was detected with a microplate reader (BioTek, Winooski, VT, USA). After culturing cells for another 24, 48, and 72 h, these operations were repeated.

### EdU assay

Five thousand cells/well were plated into 96-well plates. After overnight attachment, different reagents were added successively according to the instructions of EdU kit (Meilunbio, China). The EdU-positive cells were detected using a fluorescence microscope (Leica).

### Colony formation assay

Cells were seeded in 6-well plates (500 cells/well) and cultured for 10 days. Then, colonies were fixed (4% formaldehyde), stained (0.1% crystal violet), photographed, and counted.

### Wound healing assay

Cells were plated into 12-well plates and cultured to confluence. Then, cells were scratched with 200 μl pipette tips and cultured in medium containing 1% FBS. At 0 h and 48 h, wound closure was observed and micrographs were captured with an optical microscope (Nikon).

### Transwell assays

Transwell assays were performed using the 24-well transwell chambers (8-μm pores, NEST Biotechnology). For invasion assay, the upper chambers were precoated with 50 μl diluted Matrigel (1:8) and maintained in 37 °C incubator overnight. 50,000 cells in 200 μl serum-free medium were placed into the upper chambers, and 600 μl medium containing 20% FBS was placed into the lower chambers. After culture for another 24 h or 48 h, the nonmigrated cells were removed using cotton swabs and the migratory cells across the aperture were fixed (4% paraformaldehyde), stained (0.1% crystal violet), photographed, and counted.

### Animal experiments

According to the target sequences of si-NC and si-NPAS2#1, we constructed the shRNA lentivirus vectors and transfected into A549 cells with polybrene. The male BALB/c nude mice (five weeks old) were used to construct xenograft model by subcutaneously injecting NC and sh-NPAS2 A549 cells (5 × 10^6^) into the right axilla of each mouse. The tumor volumes were measured with the formula: tumor volume (mm^3^) = 1/2 (length × width^2^). At the experimental endpoint, the mice were killed, and the xenograft tumors were separated and fixed for further IHC analyses. The primary antibodies were listed as follows: NPAS2 (Invitrogen, PA5-98824), Ki-67 (Abcam, ab15580).

### Statistical analysis

The analyses performed in our study were executed via R software (version 4.1.0) and GraphPad 8.0. Assessment of differences between two groups was made by the Wilcoxon test or Student’s *t* test. In Kaplan–Meier survival analysis, log-rank test was utilized to compare the survival time of different groups. Correlation between numeric variables was identified by Spearman correlation analysis. *p* value < 0.05 was considered statistically significant.

## Results

### Identification of prognostic CRs

**Fig. 1:**
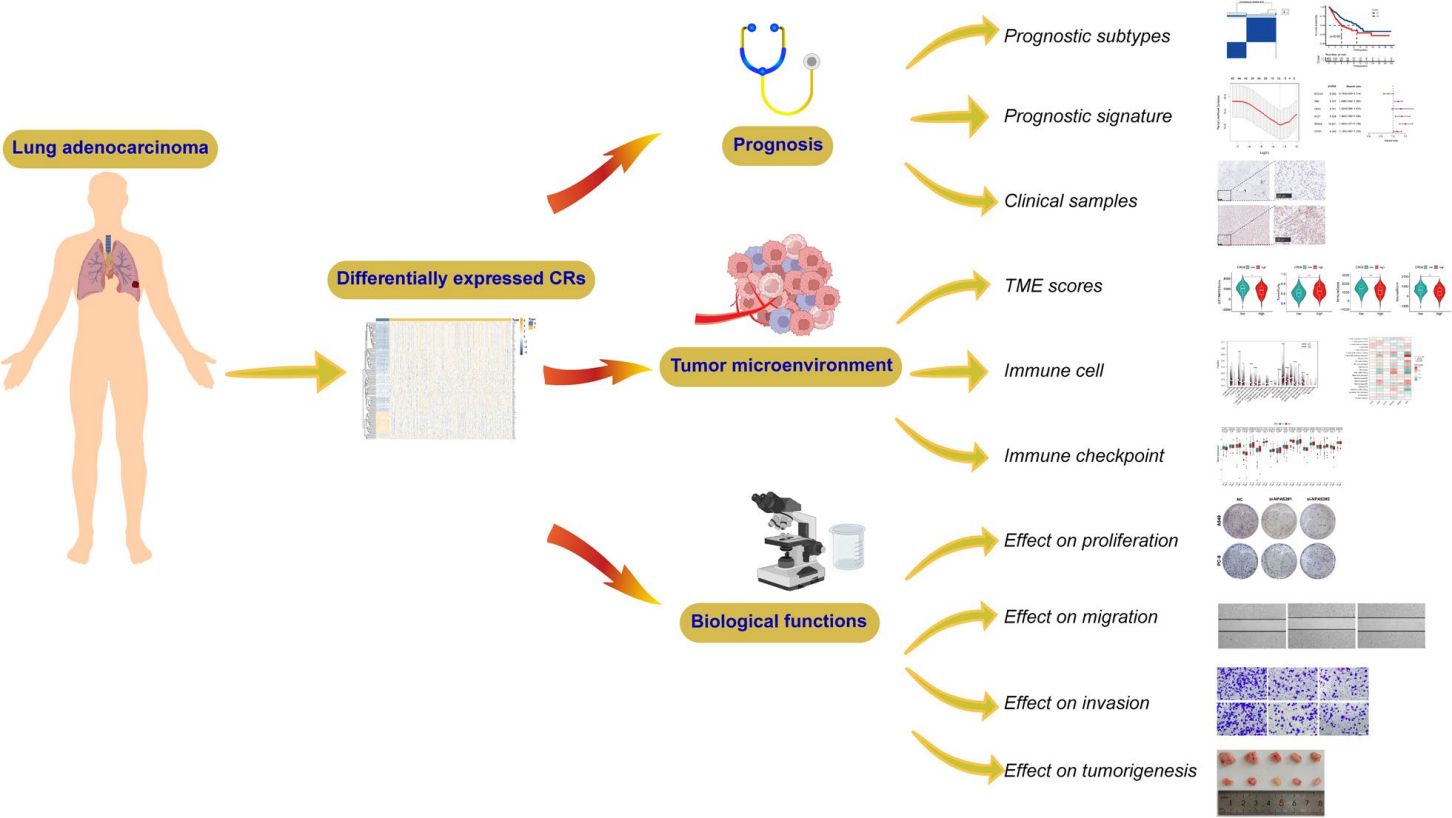
Workflow of this study

**Fig. 2:**
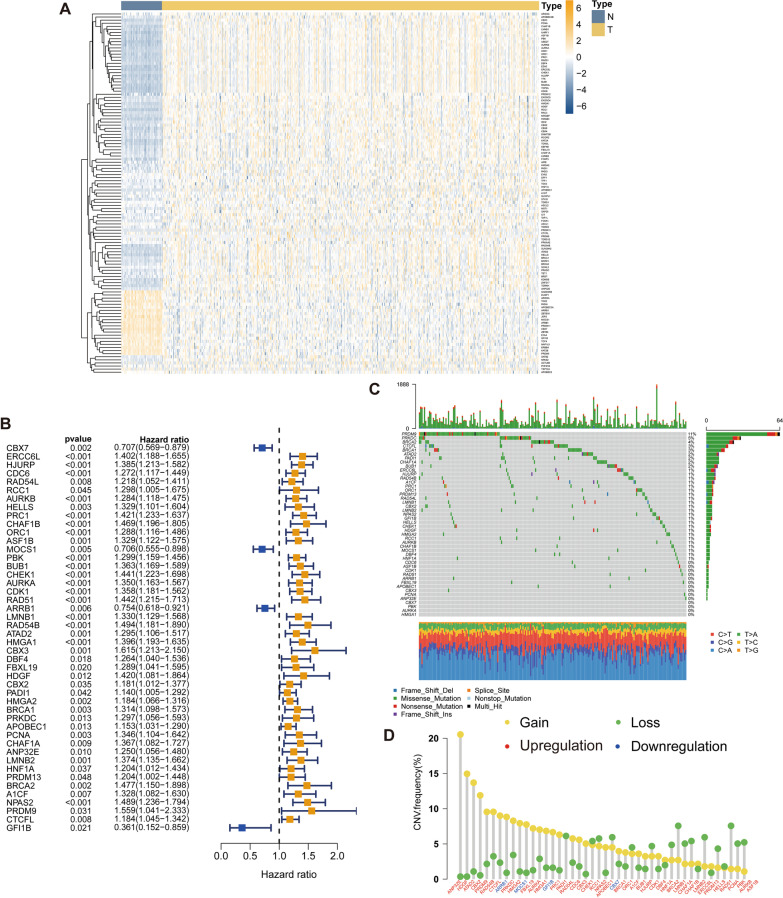
Expression and genome landscape of prognostic CRs. **A** Expression of differentially expressed CRs between LUAD and peritumoral tissues. **B** Forest plot showing effect of 46 prognostic CRs on survival. **C** Somatic mutation landscape of 46 prognostic CRs. **D** CNV frequencies of 46 prognostic CRs

The workflow of this study is presented in Fig. 1. A total of 535 LUAD and 59 para-tumoral lung samples from TCGA were included in the differential expression analysis which identified 117 dysregulated CRs in LUAD, including 21 downregulated CRs and 96 upregulated CRs (Fig. 2A). Among all the dysregulated CRs, 46 CRs were identified as contributing factors of overall survival (Fig. 2B). Somatic mutations of the prognostic CRs occurred in 37.79% of the LUAD samples with *PRDM9* had the highest mutation frequencies (Fig. 2C). Additionally, CNV of the prognostic CRs can be observed in LUAD samples (Fig. 2D).

### Characterization of LUAD subtypes based on prognostic CRs

**Fig. 3:**
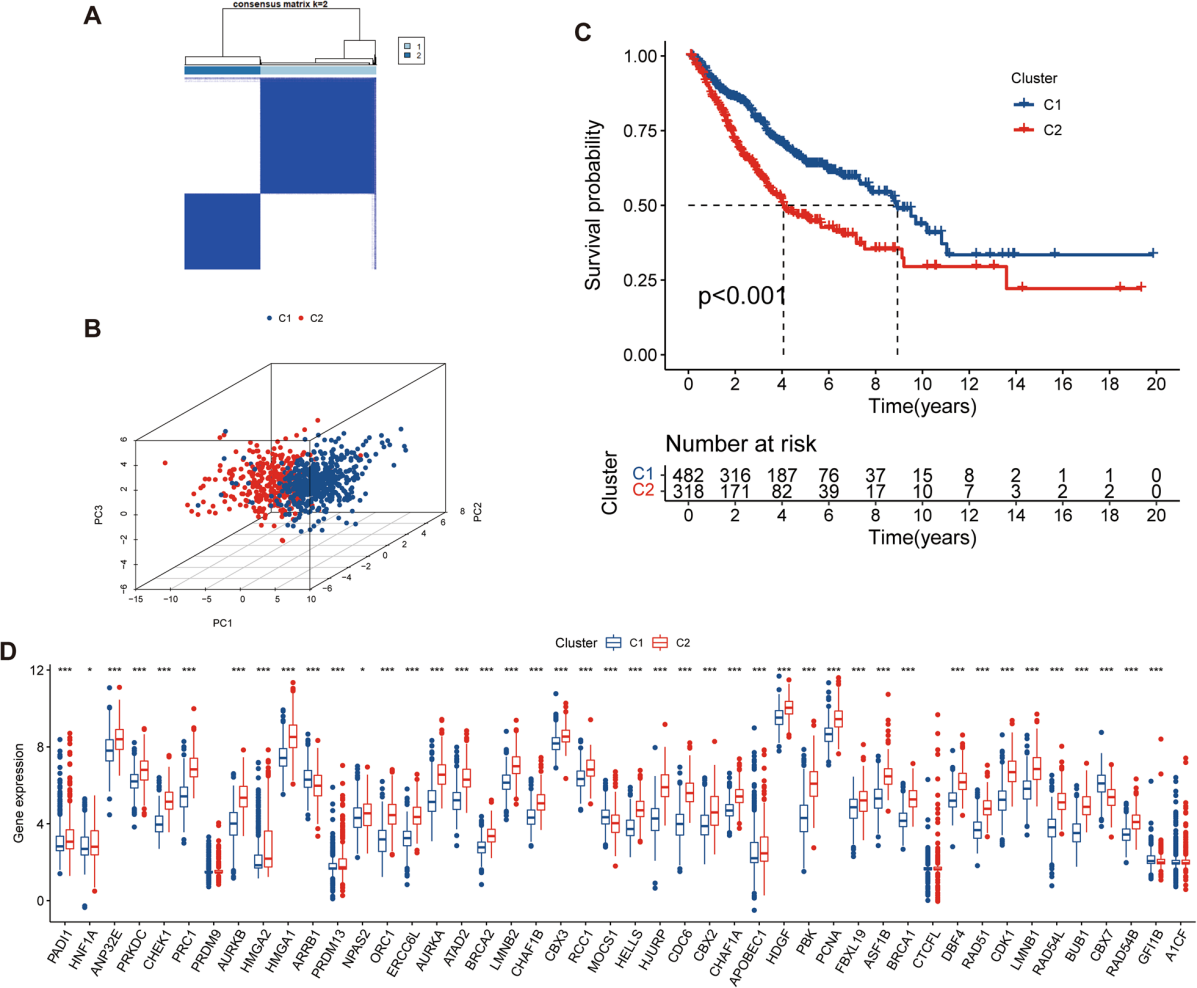
Identification of two subtypes of LUAD. **A** Two subtypes identified by consensus clustering. **B** PCA plot showing distribution of the two subtypes based on 46 prognostic CRs. **C** Survival difference between the two subtypes. **D** Differential expression of 46 prognostic CRs between two subtypes

**Fig. 4:**
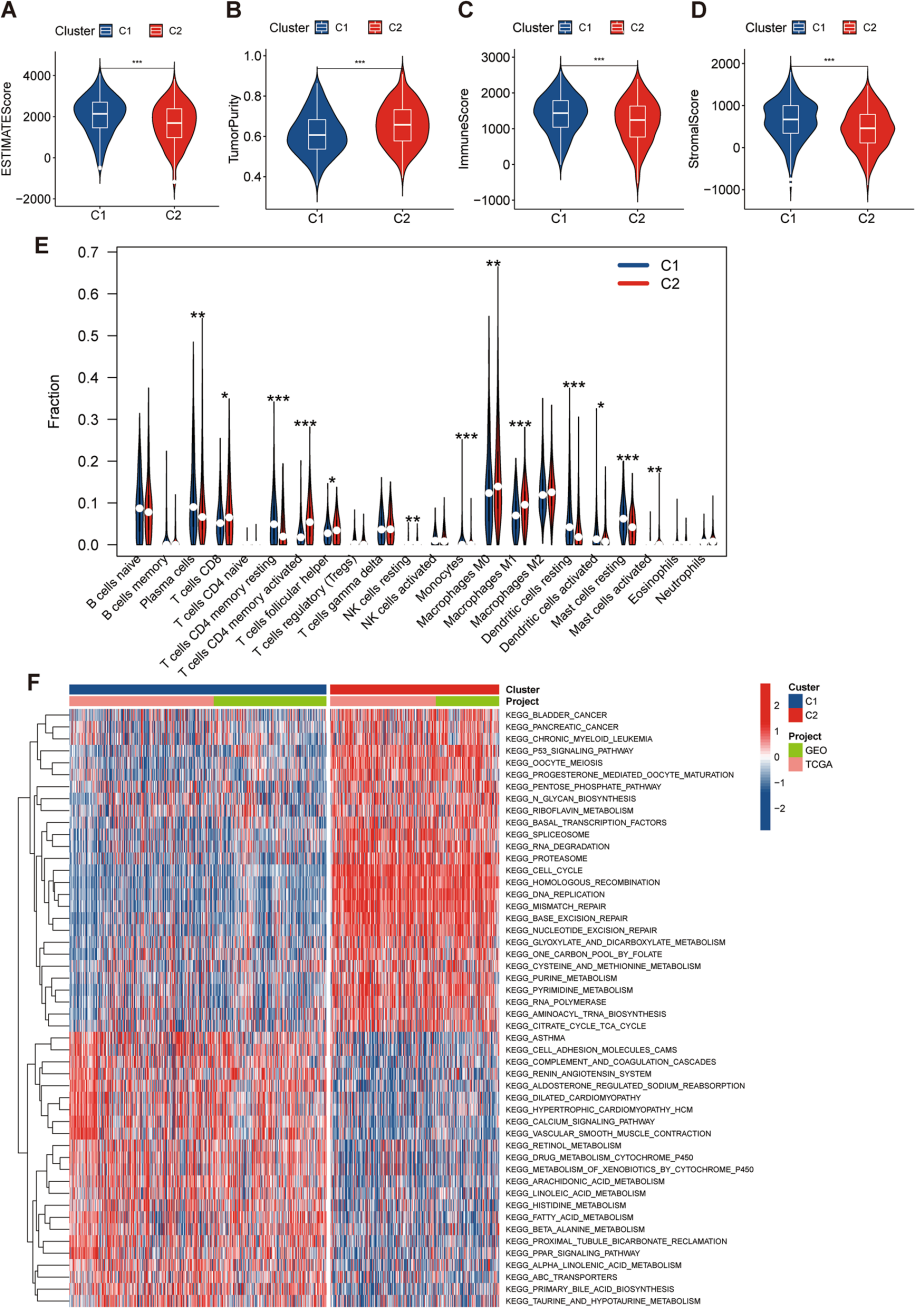
TME patterns and related-pathways of the two subtypes. **A**–**D** Differences in ESTIMATE score, tumor purity, immune score, and stromal score between the two subtypes. **E** Differences in immune cell subtypes between the two subtypes. **F** Differences in pathways between the two subtypes

A total of 800 LUAD samples were subjected to consensus clustering based on expression of 46 CRs, and 2 discrete subtypes were identified in consensus clustering (Fig. 3A). There are 318 cases in C1 and 482 cases in C2. PCA confirmed that prognostic CRs can separate C1 from C2 (Fig. 3B). Cluster 1 showed significant survival advantage over cluster 2 and had lower expression of most of the prognostic CRs than cluster 2 (Fig. 3C,  D). A total of 3827 differentially methylated sites were identified between the high- and low-CRGI groups, suggesting that patients with different CRGI had different DNA methylation levels. The most significant sites in differential methylation analysis were displayed in heatmap (Additional file 3: Fig. S1). Cluster 1 had higher abundance of stromal cells and immune cells and lower tumor purity than cluster 2 (Fig. 4A–D). Immune cell including plasma cells, monocytes, dendritic cells, and mast cells are more abundant in cluster 1 (Fig. 4E). Pathways such as bladder cancer, pancreatic cancer, and p53 signaling pathway are activated in cluster 2, while asthma, cell adhesion molecules cams, complement, and coagulation cascades are activated in cluster 1 (Fig. 4F).

### Construction of a signature based on CRs

Given that the prognostic CRs can distinguish LUAD individuals with favorable survival from those with poor survival, we constructed a signature based on prognostic CRs to predict survival for patients with LUAD. A signature made up of 6 prognostic CRs (*MOCS*, *PBK*, *CBX3*, *A1CF*, *NPAS2*, and *CTCFL*) was developed by stepwise LASSO and multivariate Cox regression analyses (Additional file 4: Fig. S2A–C). Among the CRs in the signature, *MOCS1* was tumor suppressor gene affecting survival positively, while the other genes were oncogenes whose high expression was associated with poor survival (Additional file 4: Fig. S2C). CRGI was computed using expression level of CRs and coefficients in the signature, and LUAD samples were classified into high-CRGI group and low-CRGI group (Additional file 5: Table S3). Most of the patients in the cluster 1 were classified into the low-CRGI group, and the low-CRGI group accounted for a high percentage of the living patients (Additional file 4: Fig. S2D), corresponding to the results that cluster 1 had lower CRGI than cluster 2 (Additional file 4: Fig. S2E).

### Validation of the prognostic signature

**Fig. 5:**
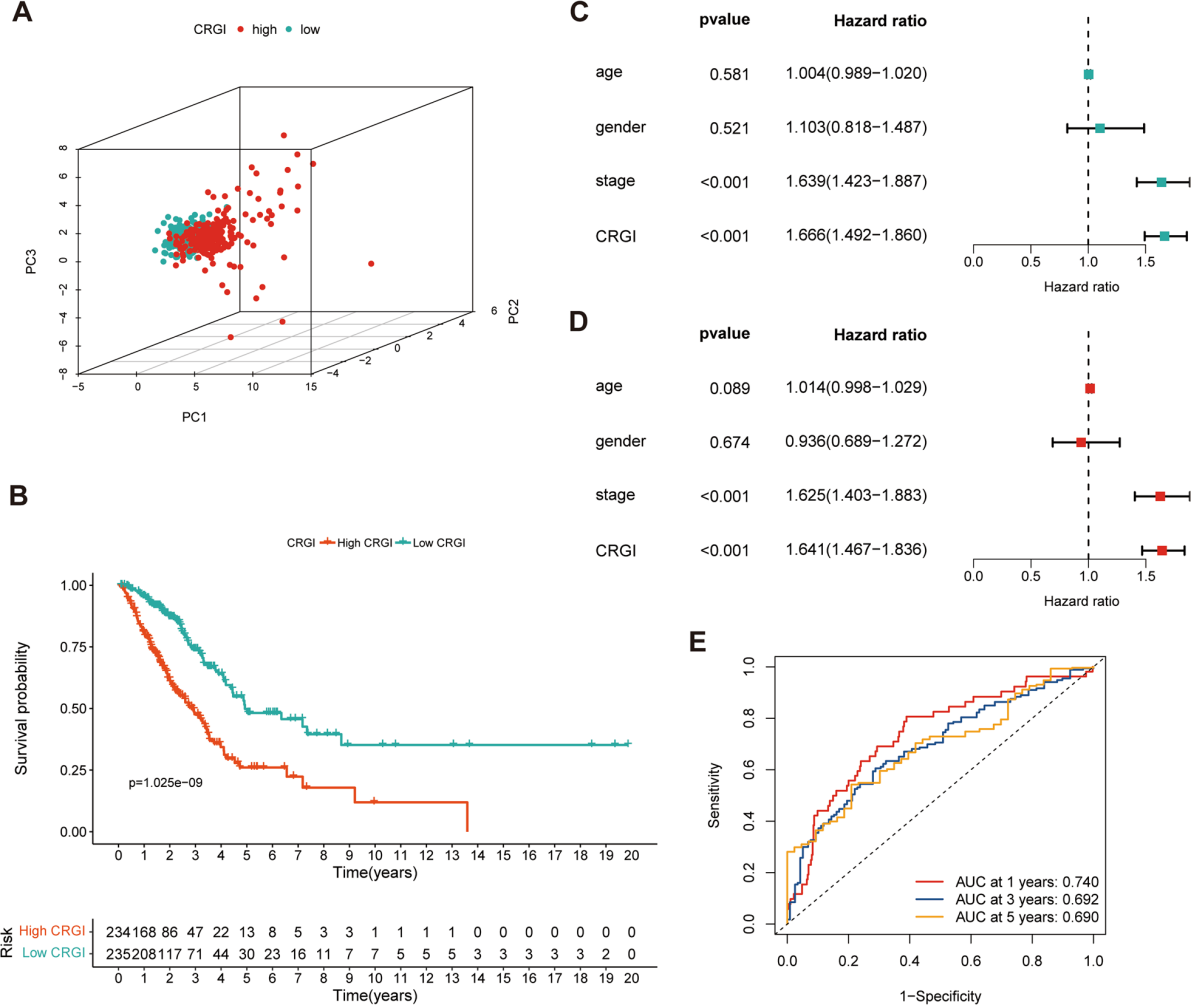
The prognostic value of CRGI in the TCGA dataset. **A** PCA plot showing distribution of the high- and low-CRGI groups. **B** Survival difference between the high- and low-CRGI groups. **C** Univariate Cox regression of CRGI and clinicopathological variables. **D** Multivariate Cox regression of CRGI and clinicopathological variables. **E** ROC curves of CRGI in survival prediction

Multiple analyses were conducted in the TCGA-LUAD dataset to show the prognostic value of CRGI. The high-CRGI group can be separated from the low-CRGI group by the expression of the CRs in the signature (Fig. 5A). Increased CRGI can be observed in nonsurvivors compared with survivors (*p* < 0.001) (Fig. 5B). Furthermore, CRGI is a risk factor of survival whose effect on survival is independent of the clinicopathological variables [hazard ratio (HR) = 1.641, 95% confidence interval (CI): 1.467–1.836, *p* < 0.001] (Fig. 5C, D). The AUCs at 1, 3, and 5 years were 0.740, 0.692, and 0.690, respectively (Fig. 5E). The prognostic value of CRGI was further examined in GSE37745 and GSE31210. Low CRGI was associated with survival advantage in both GSE37745 (*p* < 0.001) and GSE31210 (*p* = 0.0076) (Additional file 6: Fig. S3A, B). Different expression patterns of the CRs in the signature can be observed between the high- and low-CRGI groups in both GSE37745 and GSE31210 (Additional file 6: Fig. S3C, D). Cell cycle is fundamental biological process, and cell cycle-related genes are significant contributing factor of prognosis of patients with cancer [16, 17]. Some CRs in our model have been reported to be involved in cell cycle including *PBK* [18], *CBX3* [19–21]*,* and *NPAS2* [22]*.* To clarify whether the prognostic value of CRGI depended on cell cycle-related genes, we removed these genes and re-validate the prognostic value of CRGI. Analyses of survival and TME suggested that CRGI was still a predictor of outcome and immune infiltration (Additional files 7 and 8: Figs. S4, S5).

### Differences in TME between the high- and low-CRGI groups

**Fig. 6:**
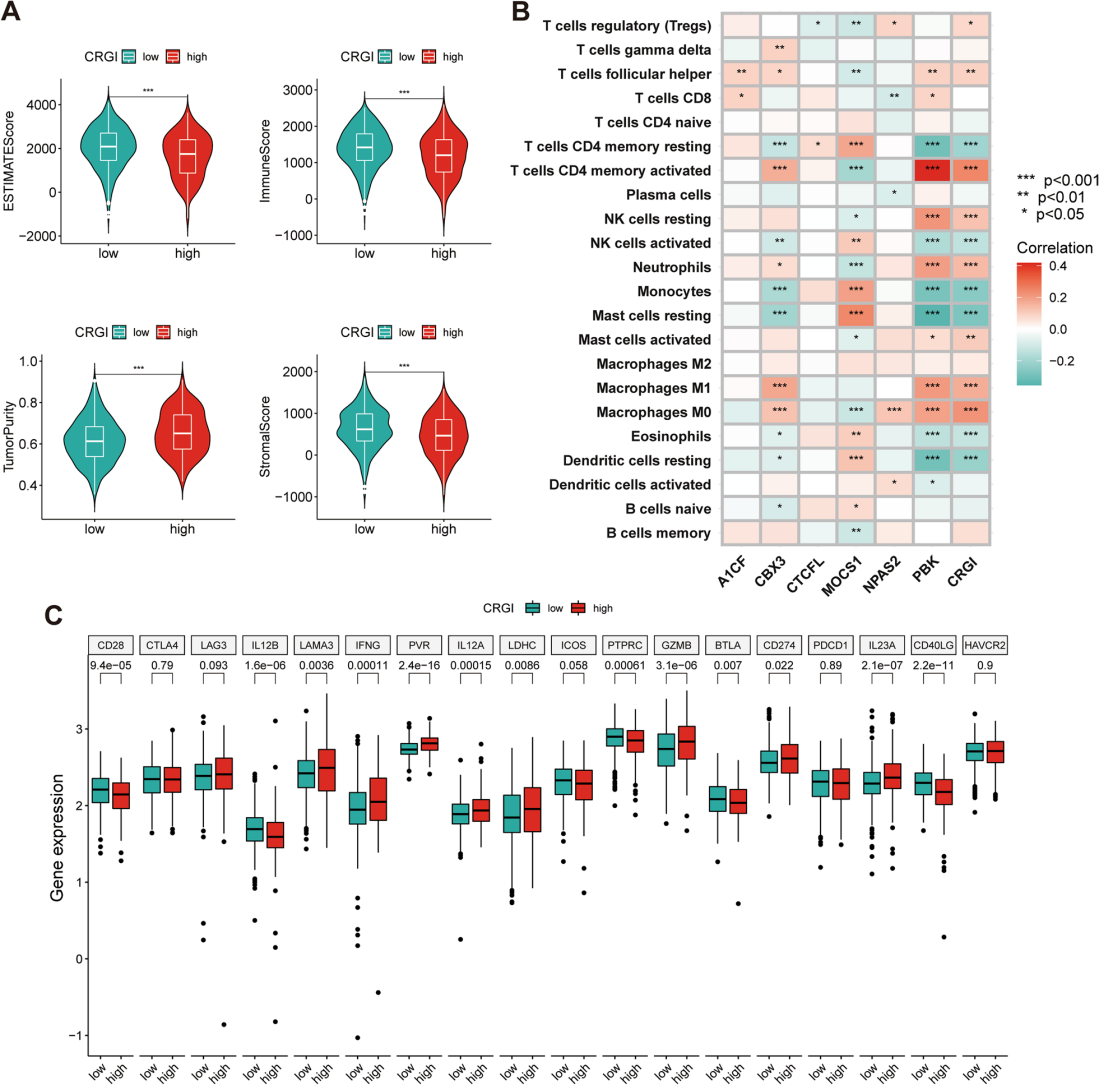
Correlation between CRGI and TME. **A** Differences in ESTIMATE score, tumor purity, immune score, and stromal score between high- and low-CRGI groups. **B** Correlation between CRGI and infiltration of immune cells. **C** Differences in expression of immune checkpoint between high- and low-CRGI groups

Compared those with the high CRGI, LUAD samples with low CRGI had higher abundance of immune cells and stromal cells (Fig. 6A). Additionally, CRGI was negatively associated with immune cells such as the mast cells, eosinophils, activated NK cells, monocytes, and CD4^+^ memory cells and positively associated with neutrophils and macrophages (Fig. 6B). The CRs in the prognostic signature were also correlated with immune infiltration (Fig. 6B). Differential expression of immune checkpoint genes such as *PD-L1*, *LAMA3*, *IFNG*, *GZMB*, and *CD28* can be observed between the high- and low-CRGI groups (Fig. 6C).

### Construction and validation of a nomogram

**Fig. 7:**
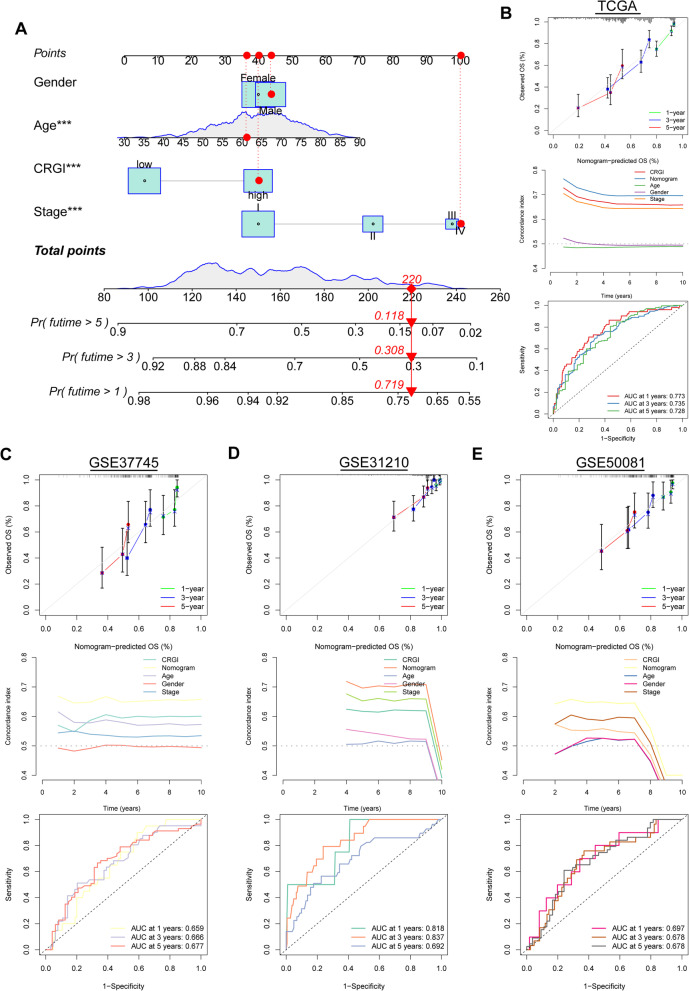
Development and validation of a nomogram. **A** Nomogram based on CRGI and clinicopathological parameters. **B** Calibration curves, C-index, and ROC curves of the nomogram in survival prediction in the TCGA. **C** Calibration curves, C-index, and ROC curves of the nomogram in survival prediction in GSE37745. **D** Calibration curves, C-index, and ROC curves of the nomogram in survival prediction in GSE31210. **E** Calibration curves, C-index, and ROC curves of the nomogram in survival prediction in GSE50081

A nomogram which consisted of CRGI and clinicopathological variables was constructed and validated in TCGA, GSE37745, GSE31210, and GSE50081 (Fig. 7A). The high consistency between predicted survival and actual survival was presented via the calibration curves (Fig. 7B–E). The results of C-index illustrated that the predictive accuracy of CRGI and clinicopathological parameters was enhanced by incorporating them into a nomogram (Fig. 7B–E). High accuracy of the nomogram was also observed from the ROC curves, indicating that the nomogram is a reliable tool for survival prediction (Fig. 7B–E).

### CRGI was associated with response to antitumor treatment

The low-CRGI group showed higher IPS which represents immunogenicity in “CTLA4^−^ PD1^−^” (*p* < 0.001), “CTLA4^−^ PD1^+^” (*p* = 0.025), “CTLA4^+^ PD1^−^” (*p* < 0.001), and “CTLA4^+^ PD1^+^” (*p* = 0.025) groups (Additional file 9: Fig. S6A), indicating that better efficacy of anti-CTLA-4 treatment and anti-PD-1 treatment may be achieved in the low-CRGI group. IC50 values of docetaxel, paclitaxel, and cisplatin are lower in the high-CRGI group compared with the low-CRGI group (*p* < 0.001) (Additional file 9: Fig. S6B), which suggests that the high-CRGI group are more sensitive to these chemotherapeutic agents. CRGI is not only a survival predictor but also a stratification tool for making optimal treatment.

### NPAS2 is highly expressed and predicts poor prognosis in LUAD

**Fig. 8:**
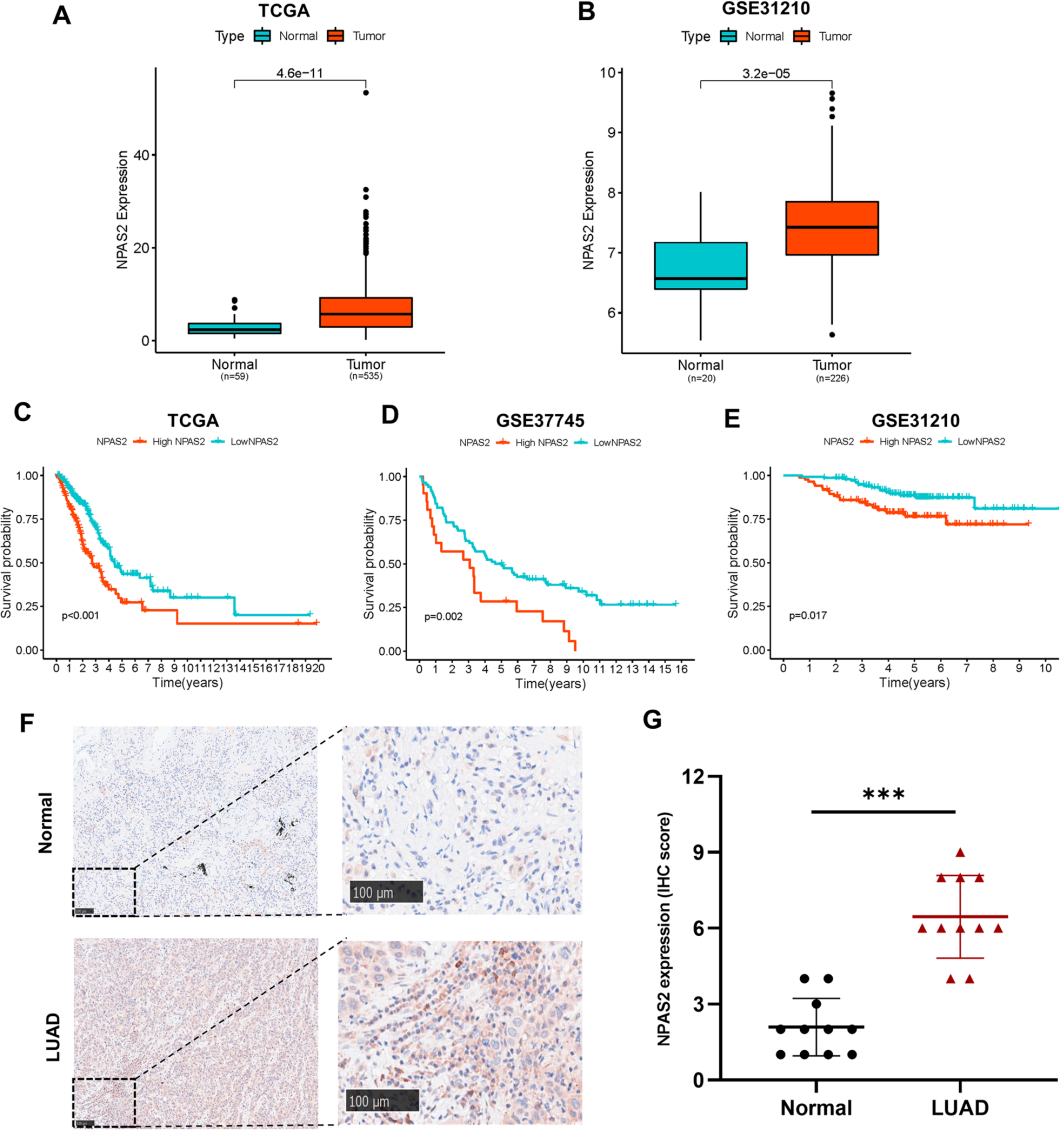
NPAS2 is overexpressed and associated with poor prognosis in LUAD. **A**–**B** The mRNA expression of NPAS2 in TCGA-LUAD and GSE31210 datasets. **C**–**E** Kaplan–Meier curves of LUAD patients based on the optimal cutoff in TCGA-LUAD, GSE37745, and GSE31210 datasets.** F**–**G** Representative images of IHC staining of NPAS2 protein and the comparison of IHC score in normal lung and LUAD tissues

*NPAS2* is the most significant contributing factor of survival in multivariate Cox regression analysis among all the signature genes. Therefore, we further investigated the mRNA expression levels and prognostic values of *NPAS2* and found that *NPAS2* is upregulated in LUAD tissues compared to normal lung tissues and predicts shorter OS (Fig. 8A–E). Furthermore, we also validated the high expression of *NPAS2* in LUAD at protein levels by using own clinical samples based on IHC staining (Fig. 8F, G).

### Knockdown of NPAS2 dampened malignant behaviors of LUAD cells

**Fig. 9:**
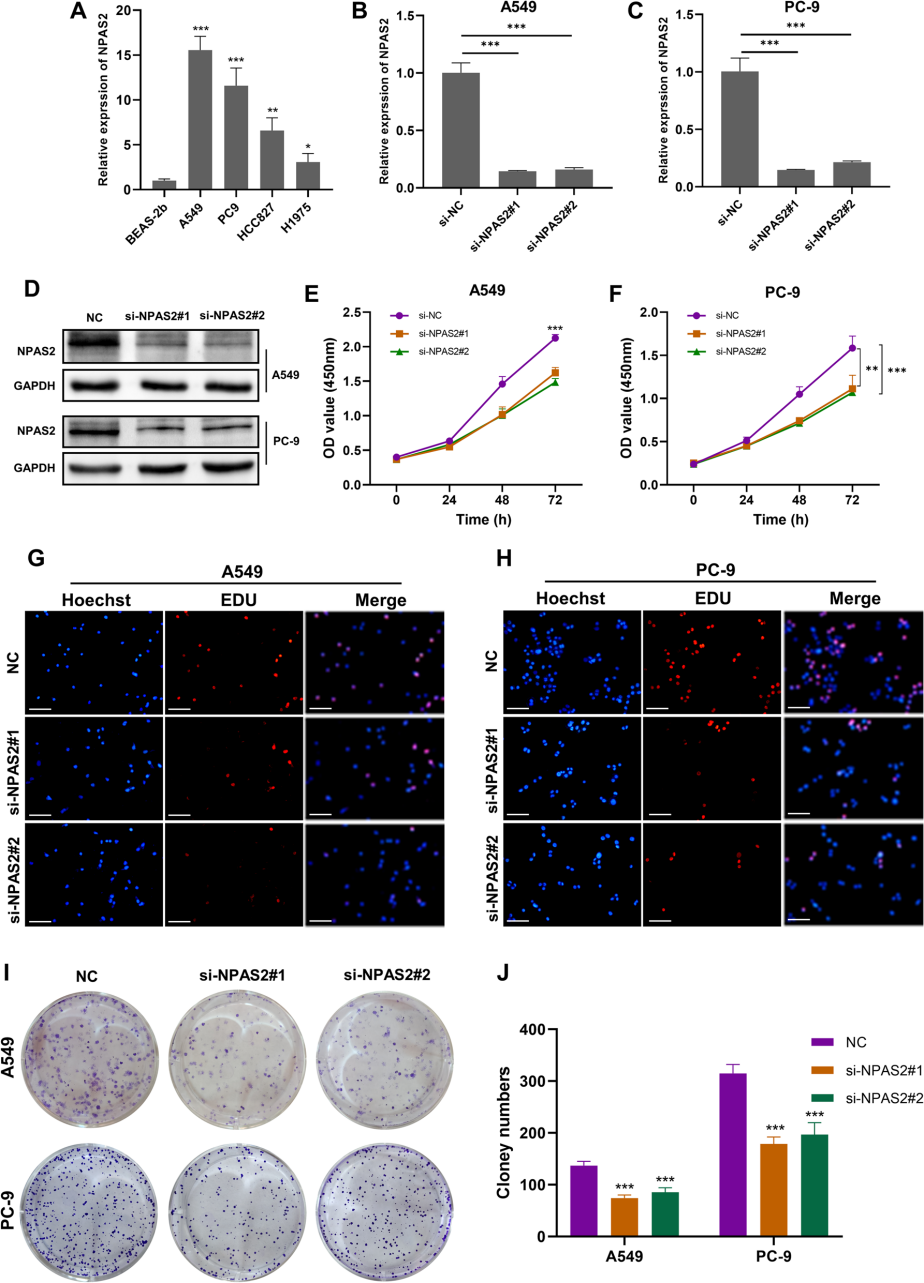
Knockdown of NPAS2 impeded proliferative activity of LUAD cells. **A** Expression of NPAS2 in BEAS-2b, A549, PC-9, HCC827, and H1975 cells. **B**–**D** Efficacy of siRNAs in suppressing expression of NPAS2 in A549 and PC-9 cells. **E**–**F** OD values of A549 and PC-9 cells in CCK8 assay after knockdown of NPAS2. **G**–**H** Proliferative activity of A549 and PC-9 cells in EdU assay after knockdown of NPAS2. **I**–**J** Colony formation assay for A549 and PC-9 cells after knockdown of NPAS2. *p < 0.05, ** *p* < 0.01, *** *p* < 0.001

The oncogenic properties of *NPAS2* have been demonstrated in various cancers like hepatocellular carcinoma and thyroid carcinoma, but the role of *NPAS2* in LUAD remains unexplored. In this study, we first investigated the mRNA expression of *NPAS2* in BEAS-2b, a normal lung epithelial cell line, and four LUAD cell lines (A549, PC-9, HCC827, and H1975) via qRT-PCR, indicating that *NPAS2* expression was significantly elevated in all LUAD cells compared with BEAS-2b (Fig. 9A). Due to the highest expression of *NPAS2* in A549 and PC-9 cells, they were selected for further experiments with siRNA knocking down. The mRNA and protein level of *NPAS2* was remarkably suppressed by si-NPAS2#1 and si-NPAS2#2 in both A549 and PC-9 cells (Fig. 9B–D). The effect of NPAS2 on LUAD cells proliferation was evaluated by CCK8, EdU, and colony formation assays. The CCK8 assays indicated that *NPAS2* knockdown impaired cell viability of A549 and PC-9 cells (Fig. 9E, F), and the results of EdU assay showed that inhibiting *NPAS2* dramatically reduced the proportion of EdU-positive cells (Fig. 9G, H). Additionally, colony formation ability of LUAD cells was remarkably attenuated after *NPAS2* loss (Fig. 9I, J). Collectively, these results suggested that *NPAS2* knockdown dramatically suppressed LUAD cells proliferation in vitro*.*

**Fig. 10:**
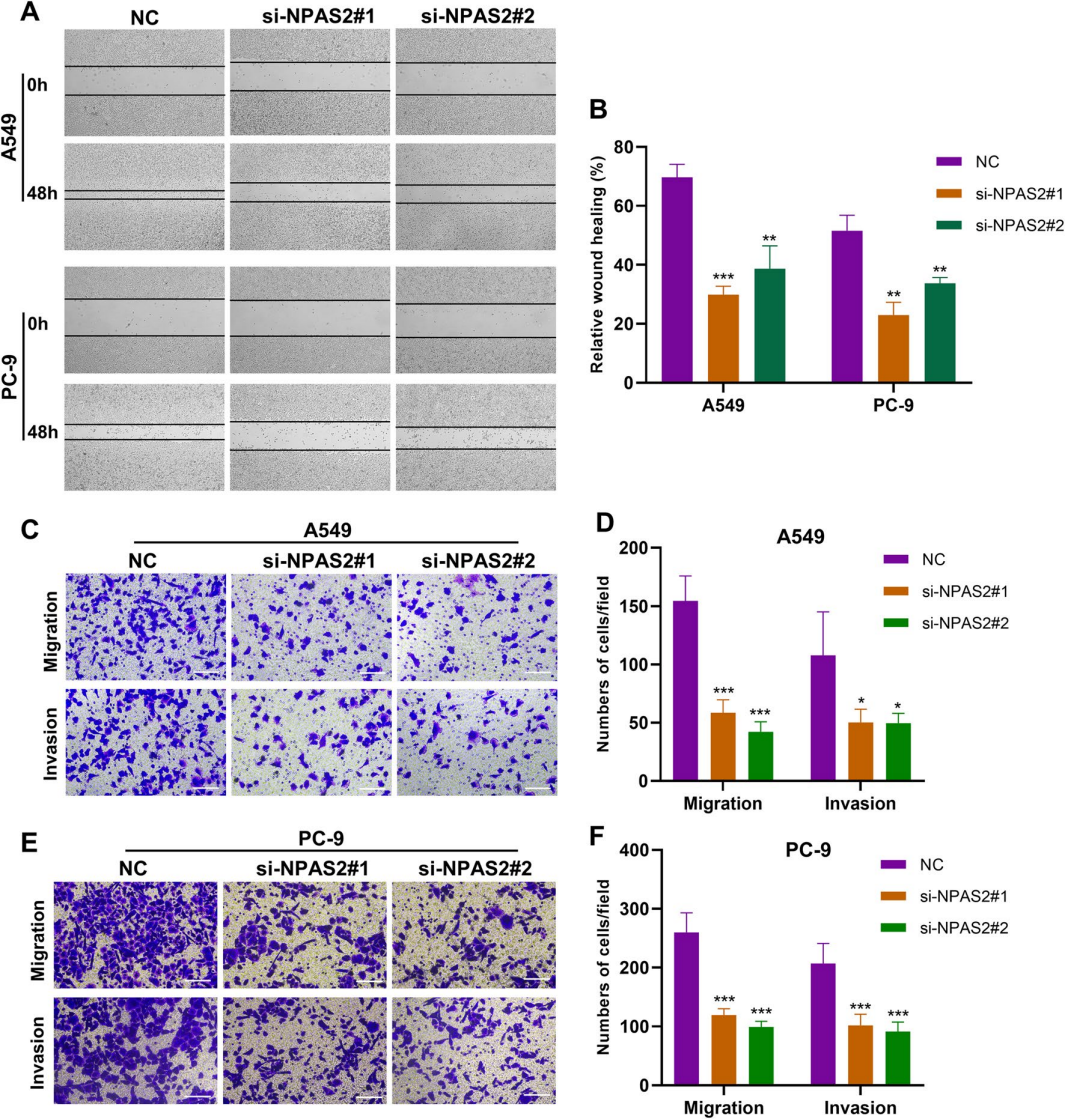
Knockdown of NPAS2 impeded migration and invasion capacities of LUAD cells. **A**–**B** Migration ability of A549 and PC9 cells in wound healing assay after knockdown of NPAS2. **C**–**F** Migration and invasion abilities of A549 and PC-9 cells in transwell assays after knockdown of NPAS2. *p < 0.05, ** *p* < 0.01, *** *p* < 0.001

Next, to assess the effect of inhibiting *NPAS2* on migration and invasion, wound healing and transwell assays were conducted in vitro. In wound healing assays, A549 and PC-9 cells with *NPAS2* suppressed migrated more slowly compared to the control group (Fig. 10A, B). Additionally, the A549 and PC-9 cells with NPAS2 knockdown exhibited weaker migration and invasive abilities in transwell assays (Fig. 10C–F). All the above results suggest that *NPAS2* is a cancer driver gene in LUAD, and that inhibition of *NPAS2* suppresses malignant progression of LUAD cells.

### *NPAS2 knockdown suppresses tumor growth of LUAD *in vivo

**Fig. 11:**
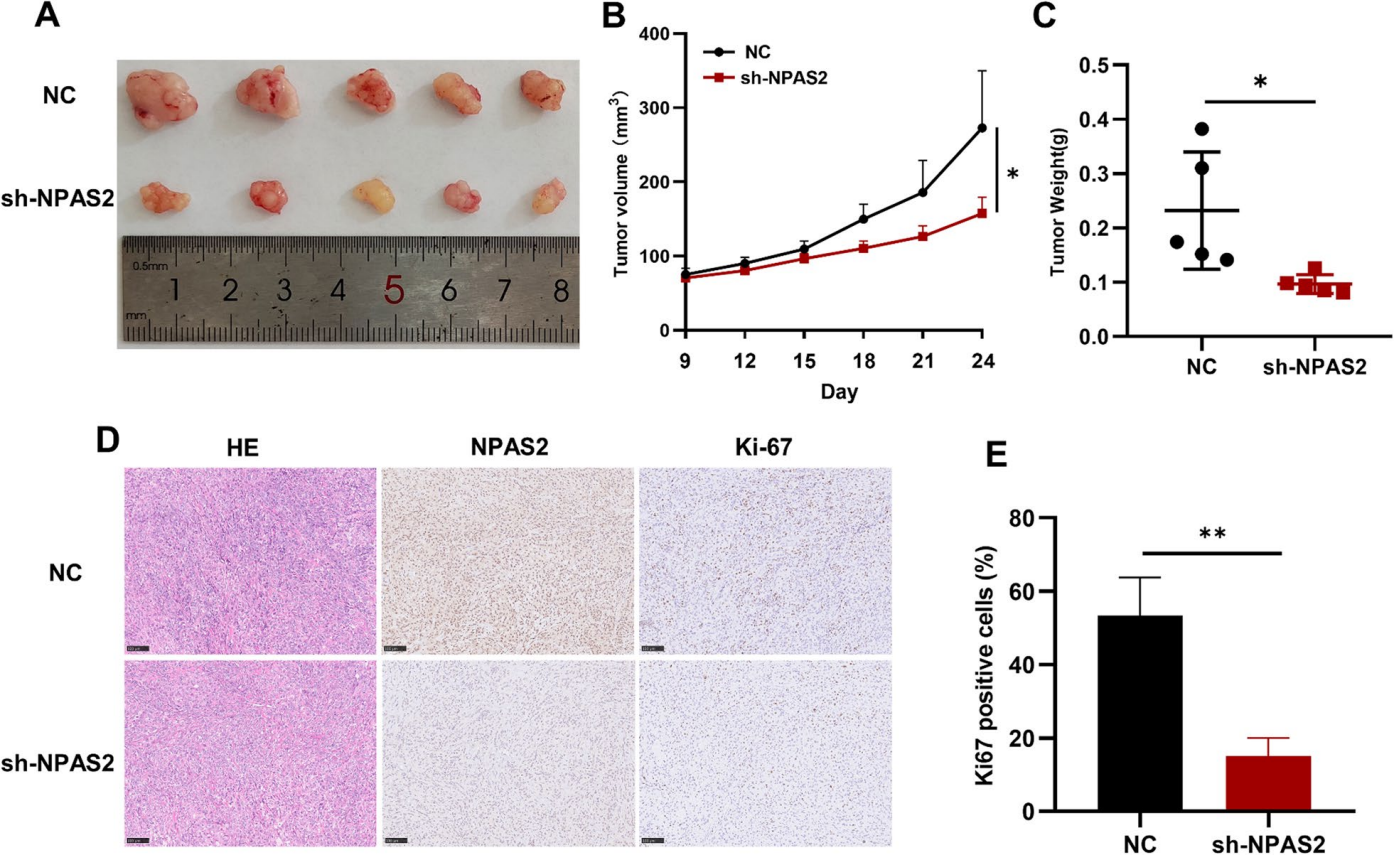
Knockdown of NPAS2 inhibited the growth of A549 cells in vivo*.* **A** Gross appearance of tumor xenografts in NC and sh-NPAS2 groups. *n* = 5. **B** Tumor growth curve of two groups. **C** The tumor weights on day 24 of each group. **D**–**E** HE staining and IHC staining of NPAS2 and Ki-67 in the tumor tissues. *p < 0.05, **p < 0.01, ***p < 0.001

To further determine the oncogenic role of *NPAS2* in LUAD in vivo, we construct xenograft model by subcutaneously injecting NC and sh-NPAS2 A549 cells in BALB/c nude mice. As we expected, we found that *NPAS2* knockdown significantly inhibited tumor growth, and the tumor volume and weight of the *NPAS2* knockdown group were notably smaller than the NC group (Fig. 11A–C). Further IHC staining confirmed that NPAS2 was down-expressing in the tumor tissues of NPAS2 knockdown group. Besides, the Ki-67 expression level was also significantly less than NC group (Fig. 11D, E). Collectively, these results demonstrated that *NPAS2* could promote tumor growth of LUAD in vivo and function as an oncogene of LUAD.

## Discussion

High malignancy and poor outcome of LUAD make it necessary to explore more robust prognostic indicators. Chromatin regulators are key molecules that affect numerous biological processes and malignant progression. In this study, we characterized two subtypes of LUAD based on expression of CRs which had significantly different prognosis and TME. Compared with the cluster 2, the cluster 1 had better survival, lower tumor purity, and higher infiltration degree of immune cells. Considering the prognostic value of CR subtypes, we construct a CR-based indicator to predict survival and TME for LUAD individuals.

Six key CRs were selected to develop a signature which was used to calculate CRGI. The survival probability of LUAD individuals decreased with the increase in CRGI in multiple LUAD datasets, suggesting that CRGI is a remarkable risk factor for patients with LUAD. The negative effect of CRGI on survival is independent of clinicopathological parameters, and the effect of CRGI on survival is more significant than the clinicopathological parameters. Therefore, CRGI had the potential to be alternative or complement to clinical parameters.

All the genes in the signature were oncogenes except *MOCS1* whose increased expression was associated with favorable prognosis. *NPAS2* is a circadian clock gene and has been reported to be a crucial regulator of tumorigenesis and immune infiltration. *NPAS2* was identified as an oncogene in hepatocellular carcinoma and was related to infiltration of immune cells [23]. In accordance with our findings, increased expression of *NPAS2* has been suggested to indicate poor survival in lung cancer [24, 25]. *NPAS2* was overexpressed in anaplastic thyroid carcinoma and facilitated malignant progression [26]. The oncogenic properties of *NPAS2* were associated with its role in promoting the Warburg effect [27]. In our study, *NPAS2* was demonstrated to prompt malignant progression of LUAD via a series of in vitro experiments, which was consistent with the bioinformatic findings and precious studies. *MOCS1* was essential for synthesis of molybdenum cofactor, and gene mutation of *MOCS1* was associated with molybdenum cofactor deficiency [28–30]. Upregulation of *MOCS1* was reported in patients with higher arterial stiffness [31]. However, the role of *MOCS1* in oncogenesis has not been studied. Our study identified the prognostic role of *MOCS1* in lung cancer for the first time. *A1CF*, also known as APOBEC1 complementation factor, regulates various cellular processes and exerts a carcinogenic role in renal cell carcinoma, endometrial cancer, and glioma through RNA editing function [32–34]. Inhibition of *A1CF* dampens the malignant behaviors of cancer cell [32]. Although *CTCFL* is not expressed in normal tissues except testis and embryonic stem cells [35, 36], increased expression of *CTCFL* could be observed in various carcinomas and was correlated with malignant behaviors and drug resistance [37–40]. *CTCFL* can induce expression of oncogenes and promote oncogenic properties of carcinomas such as gastric cancer and ovarian cancer [41–43]. Upregulation of *CTCFL* is required for resistance to *ALK* inhibition in cancer cells via promoting chromatin interactions [37]. *CTCFL* can also serve as a promising target of immunotherapy to improve prognosis of breast cancer patients [44]. *PBK* is upregulated in hepatocellular carcinoma which is a risk factor of survival and associated with antitumor immunity in hepatocellular carcinoma [45, 46]. Additionally, *PBK* promotes metastasis and resistance to oxaliplatin by regulating pathways such as ETV4-uPAR and PTEN in hepatocellular carcinoma [47, 48]. The prognostic value of *PBK* in prediction of survival and antitumor immunity was also proved in colorectal cancer [49–51]. Therefore, the prognostic CRs identified in our study also exert fundamental role in various cancers and have potential to be reliable survival predictor and therapeutic targets.

Clinicopathological variables are frequently used and effective parameters to predict prognosis of cancer in clinical practice. Therefore, CRGI and clinicopathological variables were incorporated to establish a nomogram, and survival probability of patients with LUAD could be estimated accurately according to the points obtained in nomogram. The nomogram had the highest C-index among all the predictors, which demonstrates that combining CRGI and clinicopathological variable can improve predictive ability of single parameter and that the nomogram we established is a reliable and simple tool to predict survival.

CRGI was associated with components of TME, including immune cell and stromal cell. Additionally, expression of immune checkpoint differs between LUAD samples with different CRGIs. The high-CRGI group had higher IPS than the low-CRGI group, suggesting that those with high CRGI are more suitable for immune checkpoint inhibitors. Although the low-CRGI group are more likely to respond to immunotherapy, the high-CRGI group are more sensitive to chemotherapy such as docetaxel, paclitaxel, and cisplatin since the high-CRGI group had higher IC50 values than the low-CRGI group. Thus, CRGI is not only a predictor of survival but also a useful tool for treatment selection for patients with LUAD.

In conclusion, this study characterized two CR-related subtypes of LUAD which had varying prognosis and TME. We also constructed a signature based on CRs which was used to calculate an index termed CRGI to predict survival, TME, and sensitivity to immunotherapy and chemotherapy for LUAD, contributing to making personal treatment plans for LUAD individuals. Additionally, a nomogram with high predictive accuracy was established as a simple tool for survival prediction in LUAD. Our study aids in understanding CRs in LUAD and exploring novel prognostic predictor for patients with LUAD.

## Supplementary Information

**Additional file 1**: **Table S1**. The gene list of chromatin regulators.

**Additional file 2**: **Table S2**. Clinical characteristics of LUAD samples.

**Additional file 3**: **Fig. S1**. Differentially methylated sites between Cluster C1 and C2.

**Additional file 4**: **Fig. S2**. Construction of a prognostic CR-related signature. (A–B) Variable selection in LASSO Cox regression analysis. (C) CRs selected in multivariate Cox regression analysis. (D) Correlation of CRGI with subtypes and survival status. (E) Difference in CRGI between the two subtypes.

**Additional file 5**: **Table S3**. The results of multivariate Cox regression analysis

**Additional file 6**: **Fig. S3**. Prognostic performance of CRGI in GEO datasets. (A) Survival difference between the high- and low-CRGI groups in GSE37745. (B) Survival difference between the high- and low-CRGI groups in GSE31210. (C) PCA plot showing distribution of the high- and low-CRGI groups in GSE37745. (D) PCA plot showing distribution of the high- and low-CRGI groups in GSE31210.

**Additional file 7**: **Fig. S4**. Prognostic performance of CRGI after removal of cell cycle-related CRs. (A) Survival difference between the high- and low-CRGI groups after removal of cell cycle-related CRs. (C) Univariate Cox regression of CRGI and clinicopathological variables after removal of cell cycle-related CRs. (D) Multivariate Cox regression of CRGI and clinicopathological variables after removal of cell cycle-related CRs.

**Additional file 8**: **Fig. S5**. Correlation between CRGI and TME after removal of cell cycle-related CRs. (A) Differences in ESTIMATE score, tumor purity, immune score, and stromal score between high- and low-CRGI groups after removal of cell cycle-related CRs. (B) Correlation between CRGI and infiltration of immune cells after removal of cell cycle-related CRs. (C) Differences in expression of immune checkpoint between high- and low-CRGI groups after removal of cell cycle-related CRs.

**Additional file 9**: **Fig. S6**. Relationship between CRGI and response to treatment. (A) Differences in IPS between the high- and low-CRGI groups. (A) Differences in IC50 values of chemotherapeutic agents between the high- and low-CRGI groups.

## Data Availability

The datasets analyzed in this study are available at TCGA (https://portal.gdc.cancer.gov/) and GEO (http://www.ncbi.nlm.nih.gov/geo).

## Abbreviations

CRs: Chromatin regulators
LUAD: Lung adenocarcinoma
TCGA: The cancer genome atlas
RGI: Chromatin regulator-related gene index
TME: Tumor microenvironment
CNV: Copy number variations
FDR: False discovery rate
FC: Fold change
GEO: Gene expression omnibus
DEGs: Differentially expressed genes
PCA: Principal component analysis
ESTIMATE: Estimation of STromal and Immune cells in MAlignant Tumors using Expression data
CIBERSORT: Cell-type Identification By Estimating Relative Subsets Of RNA Transcripts
GSVA: Gene set variation analysis
LASSO: Least Absolute Shrinkage and Selection Operator
ROC: Receiver operating characteristic
AUC: Area under the curve
OS: Overall survival
IPS: Immunophenoscore
IC50: Half-maximal inhibitory concentration
TCIA: The Cancer Immunome Atlas
qRT-PCR: Quantitative real-time
HR: Hazard ratio
CI: Confidence interval

## References

[1] Ganti AK, Klein AB, Cotarla I, Seal B, Chou E (2021). Update of incidence, prevalence, survival, and initial treatment in patients with non-small cell lung cancer in the US. JAMA Oncol.

[2] Lu J, Xu J, Li J, Pan T, Bai J, Wang L, Jin X, Lin X, Zhang Y, Li Y, Sahni N, Li X (2018). FACER: comprehensive molecular and functional characterization of epigenetic chromatin regulators. Nucleic Acids Res.

[3] Bediaga NG, Coughlan HD, Johanson TM, Garnham AL, Naselli G, Schröder J, Fearnley LG, Bandala-Sanchez E, Allan RS, Smyth GK, Harrison LC (2021). Multi-level remodelling of chromatin underlying activation of human T cells. Sci Rep.

[4] Morrison AJ (2020). Chromatin-remodeling links metabolic signaling to gene expression. Mol Metab.

[5] Damaschke NA, Yang B, Blute ML, Lin CP, Huang W, Jarrard DF (2014). Frequent disruption of chromodomain helicase DNA-binding protein 8 (CHD8) and functionally associated chromatin regulators in prostate cancer. Neoplasia (New York, N.Y).

[6] Yan XJ, Xu J, Gu ZH, Pan CM, Lu G, Shen Y, Shi JY, Zhu YM, Tang L, Zhang XW, Liang WX, Mi JQ, Song HD, Li KQ, Chen Z, Chen SJ (2011). Exome sequencing identifies somatic mutations of DNA methyltransferase gene DNMT3A in acute monocytic leukemia. Nat Genet.

[7] Wang Q, Liang N, Yang T, Li Y, Li J, Huang Q, Wu C, Sun L, Zhou X, Cheng X, Zhao L, Wang G, Chen Z, He X, Liu C (2021). DNMT1-mediated methylation of BEX1 regulates stemness and tumorigenicity in liver cancer. J Hepatol.

[8] Han HY, Mou JT, Jiang WP, Zhai XM, Deng K (2021). Biosci Rep.

[9] Liu H, Song Y, Qiu H, Liu Y, Luo K, Yi Y, Jiang G, Lu M, Zhang Z, Yin J, Zeng S, Chen X, Deng M, Jia X, Gu Y, Chen D, Zheng G, He Z (2020). Downregulation of FOXO3a by DNMT1 promotes breast cancer stem cell properties and tumorigenesis. Cell Death Differ.

[10] Zhu K, Liu X, Deng W, Wang G, Fu B (2022). Identification of a chromatin regulator signature and potential candidate drugs for bladder cancer. Hereditas.

[11] Johnson WE, Li C, Rabinovic A (2007). Adjusting batch effects in microarray expression data using empirical Bayes methods. Biostatistics (Oxford, England).

[12] Monti S, Tamayo P, Mesirov J, Golub T (2003). Consensus clustering: a resampling-based method for class discovery and visualization of gene expression microarray data. Mach Learn.

[13] Wilkerson MD, Hayes DN (2010). ConsensusClusterPlus: a class discovery tool with confidence assessments and item tracking. Bioinformatics (Oxford, England).

[14] Charoentong P, Finotello F, Angelova M, Mayer C, Efremova M, Rieder D, Hackl H, Trajanoski Z (2017). Pan-cancer immunogenomic analyses reveal genotype-immunophenotype relationships and predictors of response to checkpoint blockade. Cell Rep.

[15] Huang Y, Huang S, Ma L, Wang Y, Wang X, Xiao L, Qin W, Li L, Yuan X (2021). Exploring the prognostic value, immune implication and biological function of H2AFY gene in hepatocellular carcinoma. Front immunol.

[16] Bakr MN, Takahashi H, Kikuchi Y (2023). CHRNA1 and its correlated-myogenesis/cell cycle genes are prognosis-related markers of metastatic melanoma. Biochem Biophy Rep.

[17] Wu H, Liu S, Wu D, Zhou H, Sui G, Wu G (2023). Cell division cycle-associated 8 is a prognostic biomarker related to immune invasion in hepatocellular carcinoma. Cancer Med.

[18] Liu Y, Liu H, Cao H, Song B, Zhang W, Zhang W (2015). PBK/TOPK mediates promyelocyte proliferation via Nrf2-regulated cell cycle progression and apoptosis. Oncol Rep.

[19] Zhang P, Yang X, Zha Z, Zhu Y, Zhang G, Li G (2022). CBX3 regulated by miR-139 promotes the development of HCC by regulating cell cycle progression. Cell cycle (Georgetown, Tex.).

[20] Zhang H, Chen W, Fu X, Su X, Yang A (2018). CBX3 promotes tumor proliferation by regulating G1/S phase via p21 downregulation and associates with poor prognosis in tongue squamous cell carcinoma. Gene.

[21] Fan Y, Li H, Liang X, Xiang Z (2017). CBX3 promotes colon cancer cell proliferation by CDK6 kinase-independent function during cell cycle. Oncotarget.

[22] Song B, Chen Y, Liu Y, Wan C, Zhang L, Zhang W (2019). NPAS2 regulates proliferation of acute myeloid leukemia cells via CDC25A-mediated cell cycle progression and apoptosis. J Cell Biochem.

[23] Zhang Z, Liang Z, Gao W, Yu S, Hou Z, Li K, Zeng P (2022). Identification of circadian clock genes as regulators of immune infiltration in hepatocellular carcinoma. J Cancer.

[24] Tang X, Qi C, Zhou H, Liu Y (2022). A novel metabolic-immune related signature predicts prognosis and immunotherapy response in lung adenocarcinoma. Heliyon.

[25] Yao R, Zhou L, Guo Z, Zhang D, Zhang T (2021). Integrative molecular analyses of an individual transcription factor-based genomic model for lung cancer prognosis. Dis Markers.

[26] Xu T, Jin T, Lu X, Pan Z, Tan Z, Zheng C, Liu Y, Hu X, Ba L, Ren H, Chen J, Zhu C, Ge M, Huang P (2022). A signature of circadian rhythm genes in driving anaplastic thyroid carcinoma malignant progression. Cell Signal.

[27] Yuan P, Yang T, Mu J, Zhao J, Yang Y, Yan Z, Hou Y, Chen C, Xing J, Zhang H, Li J (2020). Circadian clock gene NPAS2 promotes reprogramming of glucose metabolism in hepatocellular carcinoma cells. Cancer Lett.

[28] Mayr SJ, Röper J, Schwarz G (2020). Alternative splicing of the bicistronic gene molybdenum cofactor synthesis 1 (MOCS1) uncovers a novel mitochondrial protein maturation mechanism. J Biol Chem.

[29] Spiegel R, Schwahn BC, Squires L, Confer N (2022). Molybdenum cofactor deficiency: a natural history. J Inherit Metab Dis.

[30] Reiss J, Johnson JL (2003). Mutations in the molybdenum cofactor biosynthetic genes MOCS1, MOCS2, and GEPH. Hum Mutat.

[31] Logan JG, Yun S, Bao Y, Farber E, Farber CR (2020). RNA-sequencing analysis of differential gene expression associated with arterial stiffness. Vascular.

[32] Song Y, Shao L, Xue Y, Ruan X, Liu X, Yang C, Zheng J, Shen S, Chen J, Li Z, Liu Y (2019). Inhibition of the aberrant A1CF-FAM224A-miR-590-3p-ZNF143 positive feedback loop attenuated malignant biological behaviors of glioma cells. J Exp Clin Cancer Res CR.

[33] Ni D, Yi Q, Liu J, Hu Y, Lv T, Tan G, Liu Y, Xu L, Xia H, Zhou Q, Xie Y (2020). A1CF-promoted colony formation and proliferation of RCC depends on DKK1-MEK/ERK signal axis. Gene.

[34] Liu Q, Chen CY, Chen GL (2020). High APOBEC1 complementation factor expression positively modulates the proliferation, invasion, and migration of endometrial cancer cells through regulating P53/P21 signaling pathway. Cancer Biother Radiopharm.

[35] Monk M, Hitchins M, Hawes S (2008). Differential expression of the embryo/cancer gene ECSA(DPPA2), the cancer/testis gene BORIS and the pluripotency structural gene OCT4, in human preimplantation development. Mol Hum Reprod.

[36] Loukinov DI, Pugacheva E, Vatolin S, Pack SD, Moon H, Chernukhin I, Mannan P, Larsson E, Kanduri C, Vostrov AA, Cui H, Niemitz EL, Rasko JE, Docquier FM, Kistler M, Breen JJ, Zhuang Z, Quitschke WW, Renkawitz R, Klenova EM, Feinberg AP, Ohlsson R, Morse HC, Lobanenkov VV (2002). BORIS, a novel male germ-line-specific protein associated with epigenetic reprogramming events, shares the same 11-zinc-finger domain with CTCF, the insulator protein involved in reading imprinting marks in the soma. Proc Natl Acad Sci USA.

[37] Debruyne DN, Dries R, Sengupta S, Seruggia D, Gao Y, Sharma B, Huang H, Moreau L, McLane M, Day DS, Marco E, Chen T, Gray NS, Wong KK, Orkin SH, Yuan GC, Young RA, George RE (2019). BORIS promotes chromatin regulatory interactions in treatment-resistant cancer cells. Nature.

[38] Gong M, Yan C, Jiang Y, Meng H, Feng M, Cheng W (2019). Genome-wide bioinformatics analysis reveals CTCFL is upregulated in high-grade epithelial ovarian cancer. Oncol Lett.

[39] Janssen SM, Moscona R, Elchebly M, Papadakis AI, Redpath M, Wang H, Rubin E, van Kempen LC, Spatz A (2020). BORIS/CTCFL promotes a switch from a proliferative towards an invasive phenotype in melanoma cells. Cell Death Discov.

[40] Li X, Ning L, Zhang Q, Ge Y, Liu C, Bi S, Zeng X, Nong W, Wu S, Guo G, Xiao S, Luo B, Xie X (2020). Expression profile of ACTL8, CTCFL, OIP5 and XAGE3 in glioma and their prognostic significance: a retrospective clinical study. Am J Transl Res.

[41] Soltanian S, Dehghani H (2018). BORIS: a key regulator of cancer stemness. Cancer Cell Int.

[42] Yao H, Shao Q, Shao Y (2021). Transcription factor CTCFL promotes cell proliferation, migration, and invasion in gastric cancer via activating DPPA2. Comput Math Methods Med.

[43] Hillman JC, Pugacheva EM, Barger CJ, Sribenja S, Rosario S, Albahrani M, Truskinovsky AM, Stablewski A, Liu S, Loukinov DI, Zentner GE, Lobanenkov VV, Karpf AR, Higgins MJ (2019). BORIS expression in ovarian cancer precursor cells alters the ctcf cistrome and enhances invasiveness through GALNT14. Mol Cancer Res MCR.

[44] Loukinov D (2018). Targeting CTCFL/BORIS for the immunotherapy of cancer. Cancer Immunol Immunother CII.

[45] Zhou Z, Li Y, Hao H, Wang Y, Zhou Z, Wang Z, Chu X (2019). Screening hub genes as prognostic biomarkers of hepatocellular carcinoma by bioinformatics analysis. Cell Transplant.

[46] Huang R, Liu J, Li H, Zheng L, Jin H, Zhang Y, Ma W, Su J, Wang M, Yang K (2021). Identification of hub genes and their correlation with immune infiltration cells in hepatocellular carcinoma based on GEO and TCGA databases. Front Genetics.

[47] Cao H, Yang M, Yang Y, Fang J, Cui Y (2021). PBK/TOPK promotes chemoresistance to oxaliplatin in hepatocellular carcinoma cells by regulating PTEN. Acta Biochim Biophys Sin.

[48] Yang QX, Zhong S, He L, Jia XJ, Tang H, Cheng ST, Ren JH, Yu HB, Zhou L, Zhou HZ, Ren F, Hu ZW, Gong R, Huang AL, Chen J (2019). PBK overexpression promotes metastasis of hepatocellular carcinoma via activating ETV4-uPAR signaling pathway. Cancer Lett.

[49] Lee DH, Jeong YJ, Won JY, Sim HI, Park Y, Jin HS (2022). PBK/TOPK is a favorable prognostic biomarker correlated with antitumor immunity in colon cancers. Biomedicines.

[50] Lu J, Chen Q (2021). Transcriptome-based identification of molecular markers related to the development and prognosis of Colon cancer. Nucleo Nucle Nucleic Acids.

[51] Nagano-Matsuo A, Inoue S, Koshino A, Ota A, Nakao K, Komura M, Kato H, Naiki-Ito A, Watanabe K, Nagayasu Y, Hosokawa Y, Takiguchi S, Kasugai K, Kasai K, Inaguma S, Takahashi S (2021). PBK expression predicts favorable survival in colorectal cancer patients. Virchows Arch Int J Pathol.

