# Supplementary material for: Characterization of molecular subtypes based on chromatin regulators and identification of the role of NPAS2 in lung adenocarcinoma
Source: Clin Epigenetics. 2023 Apr 29;15:72. doi: 10.1186/s13148-023-01486-w (PMC10149025; doi:10.1186/s13148-023-01486-w)
Supplement: Supplementary file 1 — Additional file 1: Table S1. The gene list of chromatin regulators. [file 13148_2023_1486_MOESM1_ESM.docx]

**Supplementary table1.** The gene list of chromatin regulators.

| **Gene symbol** | **Function** | **PMID** | **Type** |
| --- | --- | --- | --- |
| A1CF | RNA modification(RNA deamination) | 26153137 |  |
| ACTB | Chromatin remodelling cofactor | 26153137 | Chromatin Remodeler |
| ACTL6A | Chromatin remodelling cofactor;Required for maximal SMARCA4 activity and for the association of the SWI/SNF complex with chromatin. | 24063517;26153137;24240475 | Chromatin Remodeler |
| ACTL6B | Chromatin remodelling cofactor | 24063517;26153137;24240475 | Chromatin Remodeler |
| ACTR2 |  | 24240475 |  |
| ACTR3 |  | 24240475 |  |
| ACTR3B | Chromatin remodelling | 26153137 | Chromatin Remodeler |
| ACTR5 | Chromatin remodelling | 26153137;24240475 | Chromatin Remodeler |
| ACTR6 | Chromatin remodelling cofactor | 26153137;24240475 | Chromatin Remodeler |
| ACTR8 | Histone modification read | 26153137;24240475 | Histone Modifier |
| ADNP | Chromatin remodelling cofactor;Histone Modification(Acetylation, methylation and phosphorylation Reader) | 26153137;26169266 | Histone Modifier,Chromatin Remodeler |
| AEBP2 | Histone modification write cofactor(Histone methylation) | 26153137;24063517 | Histone Modifier |
| AHCTF1 |  | 24240475 |  |
| AICDA | DNA modification(DNA demethylation);DNA modification(DNA methylation editor/eraser);May play a role in DNA demethylation. | 24063517;26153137;26169266 | DNA Methylator |
| AIRE | Histone modification read, TF | 26153137;24240475 | Histone Modifier |
| AKAP1 |  | 24240475 |  |
| ALKBH1 | Histone modification;DNA modification(DNA methylation editor/eraser) | 26153137;26169266 | DNA Methylator,Histone Modifier |
| ALKBH3 | DNA modification(DNA methylation editor/eraser) | 26169266 | DNA Methylator |
| ANP32A | Chromatin remodelling cofactor | 26153137 | Chromatin Remodeler |
| ANP32B | Histone chaperone | 26153137 | Histone Modifier |
| ANP32E | Histone chaperone, Histone modification read | 26153137 | Histone Modifier |
| APBB1 | Histone modification | 26153137 | Histone Modifier |
| APEX1 | DNA modification cofactor(DNA demethylation) | 26153137 | DNA Methylator |
| APOBEC1 | DNA modification, RNA modification(DNA demethylation, mRNA editing);DNA modification(DNA methylation editor/eraser) | 26153137;26169266 | DNA Methylator |
| APOBEC2 | DNA modification, RNA modification(DNA demethylation, mRNA editing) | 26153137 | DNA Methylator |
| APOBEC3A | DNA modification, RNA modification(DNA demethylation, mRNA editing) | 26153137 | DNA Methylator |
| APOBEC3B | DNA modification, RNA modification(DNA demethylation, mRNA editing) | 26153137 | DNA Methylator |
| APOBEC3C | DNA modification, RNA modification(DNA demethylation, mRNA editing) | 26153137 | DNA Methylator |
| APOBEC3D | DNA modification, RNA modification(DNA demethylation, mRNA editing) | 26153137 | DNA Methylator |
| APOBEC3F | DNA modification, RNA modification(DNA demethylation, mRNA editing) | 26153137 | DNA Methylator |
| APOBEC3G | DNA modification, RNA modification(DNA demethylation, mRNA editing) | 26153137 | DNA Methylator |
| APOBEC3H | DNA modification, RNA modification(DNA demethylation, mRNA editing) | 26153137 | DNA Methylator |
| ARID1A | Chromatin remodelling cofactor | 26153137;24063517;24240475 | Chromatin Remodeler |
| ARID1B | Histone modification write(Histone ubiquitination) | 24063517;26153137;24240475 | Histone Modifier |
| ARID2 | Chromatin remodelling cofactor;Required for the stability of the SWI/SNF chromatin remodelling complex SWI/SNF-B. | 24063517;26153137;24240475 | Chromatin Remodeler |
| ARID3A |  | 24240475 |  |
| ARID3B |  | 24240475 |  |
| ARID3C |  | 24240475 |  |
| ARID4A | Histone modification write cofactor(Histone acetylation);Bridging molecule to recruit HDACs. | 26153137;24063517;24240475 | Histone Modifier |
| ARID4B | Histone modification write cofactor(Histone acetylation) | 26153137;24240475 | Histone Modifier |
| ARID5A |  | 24240475 |  |
| ARID5B |  | 24240475 |  |
| ARNTL | Histone modification write cofactor, TF( TF activator) | 26153137 | Histone Modifier |
| ARRB1 | Histone modification | 26153137 | Histone Modifier |
| ASCL1 |  | 22196736 |  |
| ASCL2 |  | 22196736;22196736 |  |
| ASF1A | Histone chaperone | 26153137;24240475 | Histone Modifier |
| ASF1B | Histone chaperone | 26153137;24240475 | Histone Modifier |
| ASH1L | Histone modification write(Histone methylation);Histone Modification(Histone methyltransferases(HMT));H3K36 HMT. | 24063517;26153137;26169266;24240475;22196736 | Histone Modifier |
| ASH2L | Histone modification write cofactor(Histone methylation);H3K4 HMT. Complex with MLL | 26153137;24063517;24240475 | Histone Modifier |
| ASXL1 | Histone modification erase, Polycomb group (PcG) protein(Histone deubiquitination);Associates with PRC2 to promote gene repression. | 26153137;24063517;24240475;22196736;22196736 | Histone Modifier |
| ASXL2 | Histone modification read | 26153137;24240475 | Histone Modifier |
| ASXL3 | Scaffold protein, Polycomb group (PcG) protein | 26153137;24240475 |  |
| ATAD2 | Chromatin remodelling | 26153137;24240475 | Chromatin Remodeler |
| ATAD2B | Histone modification read | 26153137;24240475 | Histone Modifier |
| ATF2 | Histone modification write, TF(Histone acetylation, TF activator);Specifically acetylates H2B and H4 in vitro. | 26153137;24063517;22196736 | Histone Modifier |
| ATF7IP | Histone modification write cofactor(Histone methylation);Mediates MBD1 transcriptional repression, couples H3K9me3 with DNA methylation. | 26153137;24063517;24063517 | DNA Methylator,Histone Modifier |
| ATM | Histone modification write(Histone phosphorylation) | 26153137 | Histone Modifier |
| ATN1 | Histone modification erase cofactor | 26153137 | Histone Modifier |
| ATR | Histone modification write(Histone phosphorylation) | 26153137 | Histone Modifier |
| ATRX | Chromatin remodelling;Nucleosome Positioning and Remodeling(Chromatin remodelling helicase);Thought to regulate deposition of H3.3 at heterochromatic regions of the genome, including telomeres | 24240475;26153137;26169266;24063517;24240475 | Histone Modifier,Chromatin Remodeler |
| ATXN7 | Histone modification write cofactor(Histone acetylation);Histone Modification(Acetylation, methylation and phosphorylation Reader) | 26153137;26169266 | Histone Modifier |
| ATXN7L3 | Histone modification erase cofactor(Histone deubiquitination) | 26153137 | Histone Modifier |
| AURKA | Histone modification write(Histone phosphorylation) | 26153137 | Histone Modifier |
| AURKB | Histone modification write(Histone phosphorylation) | 26153137 | Histone Modifier |
| AURKC | Histone modification write(Histone phosphorylation) | 26153137 | Histone Modifier |
| BABAM1 | Histone modification erase cofactor(Histone deubiquitination) | 26153137 | Histone Modifier |
| BAG6 | p300-mediated p53 acetylation upon DNA damage. May mediate H3K4me2 | 24063517 |  |
| BAHD1 | Chromatin remodelling | 26153137;24240475 | Chromatin Remodeler |
| BANF1 |  | 24240475 |  |
| BANF2 |  | 24240475 |  |
| BANP | Histone modification write(Histone acetylation) | 26153137 | Histone Modifier |
| BAP1 | Histone modification erase, Polycomb group (PcG) protein(Histone deubiquitination);Catalytic component of the PR-DUB complex, that specifically deubiquitinates H2AK119ub1. | 26153137;24063517;22196736;22196736 | Histone Modifier |
| BARD1 | Histone modification write(Histone ubiquitination) | 26153137;24240475 | Histone Modifier |
| BAZ1A | Histone chaperone | 24240475;24063517;26153137 | Histone Modifier |
| BAZ1B | Histone modification write(Histone phosphorylation);Acts as a mark that distinguishes between apoptotic and repair responses to genotoxic stress. Maintenance of chromatin structures during DNA replication processes. | 24240475;24063517;26153137 | Histone Modifier |
| BAZ2A | Chromatin remodelling, Histone modification erase( Histone deacetylation) | 24240475;24063517;26153137 | Histone Modifier,Chromatin Remodeler |
| BAZ2B | Histone modification read | 26153137;24240475 | Histone Modifier |
| BBX |  | 24240475 |  |
| BCL10 |  | 22196736 |  |
| BCOR | Polycomb group (PcG) protein | 26153137 |  |
| BCORL1 | Histone modification erase cofactor(Histone deacetylation) | 26153137 | Histone Modifier |
| BMI1 | Polycomb group (PcG) protein;Maintenance of transcriptional repression of key genes during development. H2AK119ub. | 26153137;24063517;24240475;22196736;22196736 |  |
| BOD1 |  | 24240475 |  |
| BPTF | Chromatin remodelling;Histone Modification(K4 reader);Binds H3K4me3. | 26153137;26169266;24063517;24240475 | Histone Modifier,Chromatin Remodeler |
| BRCA1 | Histone modification write cofactor, Histone modification write cofactor, Histone modification write cofactor, TF, TF(Histone acetylation, Histone methylation, Histone ubiquitination, TF activator, TF repressor) | 26153137 | Histone Modifier |
| BRCA2 | Histone modification write(Histone acetylation) | 26153137 | Histone Modifier |
| BRCC3 | Histone modification erase(Histone deubiquitination) | 26153137 | Histone Modifier |
| BRD1 | Histone modification read | 26153137;24240475 | Histone Modifier |
| BRD2 | Histone modification read | 26153137;24240475 | Histone Modifier |
| BRD3 | Histone modification read | 26153137;24240475 | Histone Modifier |
| BRD4 | Histone modification read;Histone Modification(K27 reader) | 26153137;26169266;24240475 | Histone Modifier |
| BRD7 | Histone modification read | 26153137;24240475 | Histone Modifier |
| BRD8 | Histone modification read;Histone Modification(K27 reader) | 26153137;26169266;24240475 | Histone Modifier |
| BRD9 | Histone modification read | 26153137;24240475 | Histone Modifier |
| BRDT | Histone modification read | 24240475;26153137;24240475 | Histone Modifier |
| BRE | Histone modification write cofactor(Histone ubiquitination) | 26153137 | Histone Modifier |
| BRMS1 | Chromatin remodelling | 26153137 | Chromatin Remodeler |
| BRMS1L | Histone modification erase(Histone deacetylation) | 26153137 | Histone Modifier |
| BRPF1 | Histone modification read;Histone Modification(K36 reader) | 24063517;26153137;26169266;24240475 | Histone Modifier |
| BRPF3 | Histone modification write cofactor(Histone acetylation) | 26153137;24240475 | Histone Modifier |
| BRWD1 | Histone modification read | 26153137;24240475 | Histone Modifier |
| BRWD3 | Histone modification read | 26153137;24240475 | Histone Modifier |
| BTAF1 | Nucleosome Positioning and Remodeling(Chromatin remodelling helicase) | 26169266;24240475 | Chromatin Remodeler |
| BUB1 | Histone modification write(Histone phosphorylation) | 26153137 | Histone Modifier |
| C14orf169 | Histone modification erase(Histone methylation) | 26153137 | Histone Modifier |
| C17orf49 | Histone modification read | 26153137 | Histone Modifier |
| CARM1 | Histone modification write(Histone methylation);Histone Modification(Acetylation, methylation and phosphorylation Reader) | 26153137;26169266;24240475;22196736 | Histone Modifier |
| CBX1 | Histone modification read | 26153137;24240475 | Histone Modifier |
| CBX2 | Histone modification read | 26153137;24063517;24240475 | Histone Modifier |
| CBX3 | Histone modification read;Part of PRC1-like complex 4. Binds the nuclear lamina through lamin B receptor. | 26153137;24063517;24240475 | Histone Modifier |
| CBX4 | Histone modification read | 26153137;24063517;24240475 | Histone Modifier |
| CBX5 | Histone modification read;Histone Modification(K9 reader) | 26153137;26169266;24240475 | Histone Modifier |
| CBX6 | Histone modification read | 26153137;24063517;24240475 | Histone Modifier |
| CBX7 | Histone modification read;Histone Modification(K36 reader) | 26153137;26169266;24063517;24240475 | Histone Modifier |
| CBX8 | Histone modification read;Promotes H3K9me3. Regulates cellular lifespan by repressing CDKN2A. | 26153137;24063517;24240475 | Histone Modifier |
| CDC6 | Chromatin remodelling | 26153137 | Chromatin Remodeler |
| CDC73 | Histone modification write cofactor(Histone methylation) | 26153137;22196736 | Histone Modifier |
| CDK1 | Histone modification write(Histone phosphorylation) | 26153137 | Histone Modifier |
| CDK17 | Histone modification write(Histone phosphorylation) | 26153137 | Histone Modifier |
| CDK2 | Histone modification write(Histone phosphorylation) | 26153137 | Histone Modifier |
| CDK3 | Histone modification write(Histone phosphorylation) | 26153137 | Histone Modifier |
| CDK5 | Histone modification write(Histone phosphorylation) | 26153137 | Histone Modifier |
| CDK7 | Histone modification write(Histone phosphorylation) | 26153137 | Histone Modifier |
| CDK9 | Histone modification cofactor | 26153137 | Histone Modifier |
| CDY1 | Histone modification write(Histone acetylation) | 26153137;24240475 | Histone Modifier |
| CDY2A | Histone modification write(Histone acetylation) | 26153137;24240475 | Histone Modifier |
| CDYL | Histone modification write(Histone acetylation) | 26153137;24240475 | Histone Modifier |
| CDYL2 | Histone modification read | 26153137;24240475 | Histone Modifier |
| CECR2 | Histone modification read | 26153137;24240475 | Histone Modifier |
| CENPC | DNA modification(DNA methylation) | 26153137 | DNA Methylator |
| CHAF1A | Chromatin remodelling | 26153137;24240475 | Chromatin Remodeler |
| CHAF1B | Chromatin remodelling | 26153137;24240475 | Chromatin Remodeler |
| CHD1 | Chromatin remodelling;Histone Modification(Acetylation, methylation and phosphorylation Reader);Required for the maintenance of open chromatin and pluripotency in ESC;Remodeler | 26153137;26169266;24063517;24240475;24253304;24253304;22196736;22196736;22196736 | Histone Modifier,Chromatin Remodeler |
| CHD1L | Chromatin remodelling | 26153137;24240475 | Chromatin Remodeler |
| CHD2 | Chromatin remodelling;Histone Modification(Acetylation, methylation and phosphorylation Reader);SNF2-related helicase/ATPase domains.;Remodeler | 26153137;26169266;24063517;24240475;24253304;22196736 | Histone Modifier,Chromatin Remodeler |
| CHD3 | Chromatin remodelling;Histone Modification(Acetylation, methylation and phosphorylation Reader) | 24063517;26153137;26169266;24240475;22196736 | Histone Modifier,Chromatin Remodeler |
| CHD4 | Chromatin remodelling;Histone Modification(Acetylation, methylation and phosphorylation Reader);Main component of the NuRD/Mi-2 complex;Remodeler | 24063517;26153137;26169266;24240475;24253304 | Histone Modifier,Chromatin Remodeler |
| CHD5 | Chromatin remodelling;Histone Modification(Acetylation, methylation and phosphorylation Reader) | 26153137;26169266;24240475 | Histone Modifier,Chromatin Remodeler |
| CHD6 | Chromatin remodelling;Histone Modification(Acetylation, methylation and phosphorylation Reader) | 26153137;26169266;24240475;22196736 | Histone Modifier,Chromatin Remodeler |
| CHD7 | Chromatin remodelling;Histone Modification(Acetylation, methylation and phosphorylation Reader);Remodeler | 26153137;26169266;24240475;24253304;22196736 | Histone Modifier,Chromatin Remodeler |
| CHD8 | Chromatin remodelling;Histone Modification(Acetylation, methylation and phosphorylation Reader) | 26153137;26169266;24240475 | Histone Modifier,Chromatin Remodeler |
| CHD9 | Chromatin remodelling;Histone Modification(Acetylation, methylation and phosphorylation Reader) | 26153137;26169266;24240475 | Histone Modifier,Chromatin Remodeler |
| CHEK1 | Histone modification write(Histone phosphorylation) | 26153137 | Histone Modifier |
| CHMP1A |  | 24240475 |  |
| CHMP1B |  | 24240475 |  |
| CHRAC1 | Histone chaperone | 26153137;24063517;24240475 | Histone Modifier |
| CHTOP |  | 26153137 |  |
| CHUK | Histone modification write(Histone phosphorylation) | 26153137 | Histone Modifier |
| CIC |  | 24240475 |  |
| CIR1 | Histone modification read | 26153137 | Histone Modifier |
| CIT | Histone modification write cofactor, Histone modification write cofactor(Histone methylation, Histone phosphorylation) | 26153137 | Histone Modifier |
| CLNS1A | Histone modification write cofactor(Histone methylation) | 26153137 | Histone Modifier |
| CLOCK | Histone modification write(Histone acetylation);Histone Modification(Histone acetyltransferases) | 26153137;26169266 | Histone Modifier |
| CRB2 | Histone modification read | 26153137 | Histone Modifier |
| CREBBP | Histone modification write(Histone acetylation);Histone Modification(Histone acetyltransferases);Critical role in embryonic development, acetylates both histone and non-histone proteins.;Writer | 24063517;26153137;26169266;24240475;24253304 | Histone Modifier |
| CSNK2A1 | Histone modification | 26153137 | Histone Modifier |
| CSRP2BP | Histone modification write(Histone acetylation) | 26153137 | Histone Modifier |
| CTBP1 | Chromatin remodelling | 26153137 | Chromatin Remodeler |
| CTBP2 | Histone modification write cofactor(Histone methylation) | 26153137 | Histone Modifier |
| CTCF | Chromatin remodelling, TF( TF activator);Histone Modification(histone acetyltransferase or deacetylase) | 26153137;26169266;24240475;22196736;22196736;22196736 | Chromatin Remodeler |
| CTCFL | Chromatin remodelling | 26153137 | Chromatin Remodeler |
| CTR9 | Histone modification cofactor | 26153137 | Histone Modifier |
| CUL1 | Chromatin remodelling cofactor | 26153137 | Chromatin Remodeler |
| CUL2 | Chromatin remodelling cofactor | 26153137 | Chromatin Remodeler |
| CUL3 | Histone modification write(Histone ubiquitination) | 26153137 | Histone Modifier |
| CUL4A | Histone modification write(Histone ubiquitination) | 26153137 | Histone Modifier |
| CUL4B | Histone modification write(Histone ubiquitination) | 26153137 | Histone Modifier |
| CUL5 | DNA modification cofactor(DNA methylation) | 26153137 | DNA Methylator |
| CXXC1 | Chromatin remodelling, TF | 24240475;26153137;24240475 | Chromatin Remodeler |
| DAPK3 | Histone modification write(Histone phosphorylation) | 26153137;24240475 | Histone Modifier |
| DAXX | Thought to regulate deposition of H3.3 at heterochromatic regions of the genome, including telomeres | 26153137;24063517 |  |
| DBF4 |  | 24240475 |  |
| DBF4B |  | 24240475 |  |
| DDB1 | Histone modification write(Histone ubiquitination) | 26153137 | Histone Modifier |
| DDB2 | Histone modification write cofactor(Histone ubiquitination) | 26153137 | Histone Modifier |
| DDX21 | RNA modification | 26153137 |  |
| DDX50 | RNA modification | 26153137 |  |
| DEK | Chromatin remodelling | 26153137 | Chromatin Remodeler |
| DHX30 | Histone Modification(Acetylation, methylation and phosphorylation Reader) | 26169266 | Histone Modifier |
| DIDO1 |  | 24240475 |  |
| DMAP1 | Chromatin remodelling | 26153137;24240475 | Chromatin Remodeler |
| DNAJC1 | Histone modification write cofactor, Histone modification erase cofactor(Histone acetylation, Histone deacetylation) | 26153137 | Histone Modifier |
| DNAJC2 | Histone modification read | 24240475;26153137;24240475 | Histone Modifier |
| DND1 | RNA modification | 26153137 |  |
| DNMT1 | DNA modification(DNA methylation);DNA modification(DNA methylation maintain);Maintainins methylation patterns established in development. | 26153137;26169266;24063517;24240475;22196736 | DNA Methylator |
| DNMT3A | DNA modification(DNA methylation);DNA modification(De novo methylation);Genome-wide de novomethylation, essential for the establishment of DNA methylation patterns during development. | 24240475;26153137;26169266;24063517 | DNA Methylator |
| DNMT3B | DNA modification(DNA methylation);DNA modification(De novo methylation);Genome-wide de novomethylation, essential for the establishment of DNA methylation patterns during development. | 24240475;26153137;26169266;24063517 | DNA Methylator |
| DNMT3L | Histone modification read;DNA modification(De novo methylation);Catalytically inactive, but essential for DNMT3A and DNMT3B function. | 26153137;26169266;24063517 | DNA Methylator,Histone Modifier |
| DNTT |  | 24240475 |  |
| DNTTIP2 | Chromatin remodelling | 26153137 | Chromatin Remodeler |
| DOT1L | Histone modification write(Histone methylation);Histone Modification(K79 writer);H3K79 HMT | 22196736;22196736;24063517;26153137;26169266;24240475 | Histone Modifier |
| DPF1 | Chromatin remodelling cofactor | 24063517;26153137 | Chromatin Remodeler |
| DPF2 | Chromatin remodelling | 24063517;26153137 | Chromatin Remodeler |
| DPF3 | Chromatin remodelling | 24063517;26153137 | Chromatin Remodeler |
| DPPA3 | Histone modification read | 26153137 | Histone Modifier |
| DPY30 | Histone modification write cofactor(Histone methylation) | 26153137 | Histone Modifier |
| DR1 | Histone chaperone | 26153137 | Histone Modifier |
| DTX3L | Histone modification write(Histone ubiquitination) | 26153137 | Histone Modifier |
| DUSP1 | Histone Modification(Phosphorylation editor) | 22196736;26169266 | Histone Modifier |
| DZIP3 | Histone modification write(Histone ubiquitination) | 26153137 | Histone Modifier |
| E2F6 | TF(TF repressor) | 26153137 |  |
| EED | Polycomb group (PcG) protein;Histone Modification(histone deacetylation);Different isoforms determine PRC3 or PRC4 PRC2 variants. | 26153137;26169266;24063517;24240475 | Histone Modifier |
| EHMT1 | Histone modification write(Histone methylation);Histone Modification(Histone methyltransferases(HMT));H3K9me1/me2 HMT. | 26153137;26169266;24063517;24240475;22196736 | Histone Modifier |
| EHMT2 | Histone modification write(Histone methylation);Histone Modification(Histone methyltransferases(HMT));H3K9me1/me2, H3K27me HMT. | 24063517;26153137;26169266;24240475 | Histone Modifier |
| EID1 | Histone modification write cofactor(Histone acetylation) | 26153137 | Histone Modifier |
| EID2 | Histone modification write cofactor(Histone acetylation) | 26153137 | Histone Modifier |
| EID2B | Histone modification erase cofactor(Histone acetylation) | 26153137 | Histone Modifier |
| ELP2 | Histone modification write cofactor(Histone acetylation) | 26153137 | Histone Modifier |
| ELP3 | Histone modification write(Histone acetylation) | 26153137;24240475 | Histone Modifier |
| ELP4 | Histone modification write cofactor(Histone acetylation) | 26153137 | Histone Modifier |
| ELP5 | Histone modification write cofactor(Histone acetylation) | 26153137 | Histone Modifier |
| ELP6 | Histone modification write cofactor(Histone acetylation) | 26153137 | Histone Modifier |
| EMSY | Histone modification write cofactor(Histone methylation) | 26153137 | Histone Modifier |
| ENY2 | Histone modification erase cofactor(Histone ubiquitination) | 26153137 | Histone Modifier |
| EP300 | Histone modification write(Histone acetylation);Histone Modification(Histone acetyltransferases);Acetylates all four core histones, and non-histone proteins like p53 and MyoD;Writer | 22196736;24253304;26153137;26169266;24063517;24240475 | Histone Modifier |
| EP400 | Chromatin remodelling, Histone modification write( Histone acetylation);Histone Modification(Acetylation, methylation and phosphorylation Reader);Regulates nucleosome stability during DNA repair | 26153137;26169266;24063517;24240475 | Histone Modifier,Chromatin Remodeler |
| EPC1 | Polycomb group (PcG) protein | 26153137;24240475 |  |
| EPC2 | Chromatin remodelling | 26153137;24240475 | Chromatin Remodeler |
| ERBB4 | Histone modification cofactor | 26153137 | Histone Modifier |
| ERCC6 | Chromatin remodelling;Nucleosome Positioning and Remodeling(Chromatin remodelling helicase) | 26153137;26169266;24240475 | Chromatin Remodeler |
| ERCC6L |  | 24240475 |  |
| ERCC6L2 |  | 24240475 |  |
| EXOSC1 | Scaffold protein, RNA modification( RNA degradation) | 26153137 |  |
| EXOSC2 | Scaffold protein, RNA modification( RNA degradation) | 26153137 |  |
| EXOSC3 | Scaffold protein, RNA modification( RNA degradation) | 26153137 |  |
| EXOSC4 | Scaffold protein, RNA modification( RNA degradation) | 26153137 |  |
| EXOSC5 | Scaffold protein, RNA modification( RNA degradation) | 26153137 |  |
| EXOSC6 | Scaffold protein, RNA modification( RNA degradation) | 26153137 |  |
| EXOSC7 | Scaffold protein, RNA modification( RNA degradation) | 26153137 |  |
| EXOSC8 | Scaffold protein, RNA modification( RNA degradation) | 26153137 |  |
| EXOSC9 | Scaffold protein, RNA modification( RNA degradation) | 26153137 |  |
| EYA1 | Histone modification erase(Histone phosphorylation);Histone Modification(Phosphorylation editor) | 26153137;26169266 | Histone Modifier |
| EYA2 | Histone modification erase(Histone phosphorylation);Histone Modification(Phosphorylation editor) | 26153137;26169266 | Histone Modifier |
| EYA3 | Histone modification erase(Histone phosphorylation);Histone Modification(Phosphorylation editor) | 26153137;26169266 | Histone Modifier |
| EYA4 | Histone modification erase(Histone phosphorylation);Histone Modification(Phosphorylation editor) | 26153137;26169266 | Histone Modifier |
| EZH1 | Histone modification write, Polycomb group (PcG) protein(Histone methylation);Histone Modification(Histone methyltransferases(HMT));H3K27me1/me2/me3 HMT. Less critical for H3K27me3 formation than EZH2.;Writer | 26153137;26169266;24063517;24240475;24253304;22196736;22196736 | Histone Modifier |
| EZH2 | Histone modification write, Polycomb group (PcG) protein(Histone methylation);Histone Modification(Histone methyltransferases(HMT));H3K27me1/me2/me3 HMT. Major role in stem cell identity maintenance. Also methylates GATA4. Catalytic subunit of PRC2 complex.;Writer | 26153137;26169266;24063517;24063517;24240475;24253304;22196736;22196736;22196736;22196736;22196736 | Histone Modifier |
| FAM175A | Scaffold protein | 26153137 |  |
| FAM175B | Histone modification erase cofactor(Histone ubiquitination) | 26153137 | Histone Modifier |
| FBL | Histone modification write(Histone methylation) | 26153137 | Histone Modifier |
| FBRS | Histone modification | 26153137 | Histone Modifier |
| FBRSL1 | Histone modification | 26153137 | Histone Modifier |
| FBXL19 |  | 24240475 |  |
| FMR1 |  | 24240475 |  |
| FOXA1 | Chromatin remodelling, TF | 26153137 | Chromatin Remodeler |
| FOXO1 | TF | 26153137 |  |
| FOXP1 | TF | 26153137 |  |
| FOXP2 | TF | 26153137 |  |
| FOXP3 | TF | 26153137 |  |
| FOXP4 | TF | 26153137 |  |
| FTO | DNA modification(DNA methylation editor/eraser) | 26169266 | DNA Methylator |
| FXR1 |  | 24240475 |  |
| FXR2 |  | 24240475 |  |
| GABRG1 | Histone Modification(Acetylation, methylation and phosphorylation Reader) | 26169266 | Histone Modifier |
| GADD45A | Chromatin remodelling | 26153137 | Chromatin Remodeler |
| GADD45B | Chromatin remodelling | 26153137 | Chromatin Remodeler |
| GADD45G | Chromatin remodelling | 26153137 | Chromatin Remodeler |
| GATAD1 | Histone modification read | 26153137 | Histone Modifier |
| GATAD2A | Histone modification read;Histone Modification(Acetylation, methylation and phosphorylation Reader) | 26153137;26169266;24063517 | Histone Modifier |
| GATAD2B | Histone modification read;Histone Modification(Acetylation, methylation and phosphorylation Reader) | 26153137;26169266;24063517 | Histone Modifier |
| GFI1 | Chromatin remodelling | 26153137 | Chromatin Remodeler |
| GFI1B | Histone modification cofactor | 26153137 | Histone Modifier |
| GLYATL1 | Histone Modification(K27 writer) | 26169266 | Histone Modifier |
| GLYR1 | Histone modification read | 26153137;24240475 | Histone Modifier |
| GSE1 | Histone modification erase(Histone acetylation) | 26153137 | Histone Modifier |
| GSG2 | Histone modification write(Histone phosphorylation) | 26153137 | Histone Modifier |
| GTF2I | TF | 26153137 |  |
| GTF3C1 | Histone Modification(Histone acetyltransferases) | 26169266 | Histone Modifier |
| GTF3C4 | Histone modification write(Histone acetylation) | 26153137 | Histone Modifier |
| HAT1 | Histone modification write(Histone acetylation) | 26153137;24063517;24240475 | Histone Modifier |
| HBP1 |  | 24240475 |  |
| HCFC1 | Chromatin remodelling;Histone Modification(Acetylation, methylation and phosphorylation Reader) | 26153137;26169266 | Histone Modifier,Chromatin Remodeler |
| HCFC2 | Histone modification write cofactor, Histone modification write cofactor(Histone methylation, Histone acetylation);Histone Modification(Acetylation, methylation and phosphorylation Reader) | 26153137;26169266 | Histone Modifier |
| HDAC1 | Histone modification erase(Histone acetylation);Histone Modification(K27 eraser);Eraser | 26153137;26169266;24063517;24063517;24240475;24253304;22196736;22196736;22196736;22196736 | Histone Modifier |
| HDAC10 | Histone modification erase(Histone acetylation);Histone Modification(K27 eraser) | 26153137;26169266;24063517;24240475 | Histone Modifier |
| HDAC11 | Histone modification erase(Histone acetylation);Histone Modification(K27 eraser) | 26153137;26169266;24063517;24240475 | Histone Modifier |
| HDAC2 | Histone modification erase(Histone acetylation);Histone Modification(K27 eraser);Eraser | 26153137;26169266;24063517;24063517;24240475;24253304;22196736;22196736 | Histone Modifier |
| HDAC3 | Histone modification erase(Histone acetylation);Histone Modification(K27 eraser);Modulation of cell growth and apoptosis by down-regulation of p53.;Eraser | 26153137;26169266;24063517;24240475;24253304;22196736 | Histone Modifier |
| HDAC4 | Histone modification erase(Histone acetylation);Histone Modification(K27 eraser) | 26153137;26169266;24063517;24240475 | Histone Modifier |
| HDAC5 | Histone modification erase(Histone acetylation);Histone Modification(K27 eraser) | 26153137;26169266;24063517;24240475 | Histone Modifier |
| HDAC6 | Histone modification erase(Histone acetylation);Histone Modification(K27 eraser);Eraser | 26153137;26169266;24063517;24240475;24253304;22196736 | Histone Modifier |
| HDAC7 | Histone modification erase(Histone acetylation);Histone Modification(K27 eraser) | 26153137;26169266;24063517;24240475 | Histone Modifier |
| HDAC8 | Histone modification erase(Histone acetylation);Histone Modification(K27 eraser) | 26153137;26169266;24063517;24240475 | Histone Modifier |
| HDAC9 | Histone modification erase(Histone acetylation);Histone Modification(K27 eraser);Protects neurons from apoptosis. | 26153137;26169266;24063517;24240475 | Histone Modifier |
| HDGF | Chromatin remodelling, TF( TF repressor) | 26153137 | Chromatin Remodeler |
| HELLS | Chromatin remodelling;Nucleosome Positioning and Remodeling(Chromatin remodelling helicase) | 24240475;26153137;26169266;24240475 | Chromatin Remodeler |
| HIF1AN | Histone modification erase cofactor(Histone acetylation) | 26153137 | Histone Modifier |
| HINFP | Histone modification read, TF, TF( TF activator, TF repressor) | 26153137 | Histone Modifier |
| HIRA | Histone modification read;general repressor | 21119629;26153137;24240475 | Histone Modifier |
| HIRIP3 | Histone modification read | 26153137 | Histone Modifier |
| HJURP | Histone chaperone | 26153137 | Histone Modifier |
| HLCS | Histone modification write | 26153137 | Histone Modifier |
| HLTF | Chromatin remodelling cofactor | 24240475;26153137;24240475 | Chromatin Remodeler |
| HMG20A | Chromatin remodelling cofactor | 26153137 | Chromatin Remodeler |
| HMG20B | Chromatin remodelling | 26153137 | Chromatin Remodeler |
| HMGA1 |  | 24240475 |  |
| HMGA2 |  | 24240475 |  |
| HMGB1 | Chromatin remodelling | 26153137;24240475 | Chromatin Remodeler |
| HMGB2 |  | 24240475 |  |
| HMGB3 |  | 24240475 |  |
| HMGN1 | Chromatin remodelling | 26153137;24240475 | Chromatin Remodeler |
| HMGN2 | Chromatin remodelling | 26153137;24240475 | Chromatin Remodeler |
| HMGN3 | Chromatin remodelling | 26153137;24240475 | Chromatin Remodeler |
| HMGN4 | Chromatin remodelling | 26153137;24240475 | Chromatin Remodeler |
| HMGN5 | Chromatin remodelling | 26153137;24240475 | Chromatin Remodeler |
| HN1 |  | 24240475 |  |
| HN1L |  | 24240475 |  |
| HNF1A | Possible regulation of transcription through chromatin remodelling | 24063517 | Chromatin Remodeler |
| HNRNPA1 |  | 22196736 |  |
| HP1BP3 | Chromatin remodelling | 26153137 | Chromatin Remodeler |
| HR | Histone modification erase(Histone methylation) | 26153137 | Histone Modifier |
| HSPA1A | Histone modification write cofactor, Histone modification write cofactor(Histone methylation, Histone acetylation) | 26153137 | Histone Modifier |
| HUWE1 | Histone modification write(Histone ubiquitination) | 26153137 | Histone Modifier |
| IDH1 | DNA modification(DNA methylation editor/eraser) | 26169266 | DNA Methylator |
| IDH2 | DNA modification(DNA methylation editor/eraser) | 26169266 | DNA Methylator |
| IFIT3 |  | 22196736 |  |
| IGFBP7 |  | 22196736 |  |
| IKBKAP | Scaffold protein | 26153137 |  |
| IKZF1 | Chromatin remodelling, TF;Targets NuRD/Mi-2 and SWI/SNF complexes in a single complex | 26153137;24063517 | Chromatin Remodeler |
| IKZF3 | TF | 26153137 |  |
| ING1 | Histone modification read | 26153137;24240475 | Histone Modifier |
| ING2 | Histone modification read | 26153137;24240475 | Histone Modifier |
| ING3 | Chromatin remodelling, Histone modification write cofactor( Histone acetylation) | 26153137;24240475 | Histone Modifier,Chromatin Remodeler |
| ING4 | Histone modification read;Facilitates targeting of HBO1-mediated acetylation to H3K4me3 sites. | 26153137;24063517;24240475 | Histone Modifier |
| ING5 | Histone modification read | 26153137;24240475 | Histone Modifier |
| INO80 | Chromatin remodelling;Nucleosome Positioning and Remodeling(Chromatin remodelling helicase) | 26153137;26169266;24063517;24240475 | Chromatin Remodeler |
| INO80B | Chromatin remodelling cofactor | 26153137 | Chromatin Remodeler |
| INO80C | Chromatin remodelling cofactor | 26153137 | Chromatin Remodeler |
| INO80D | Chromatin remodelling cofactor | 26153137 | Chromatin Remodeler |
| INO80E | Chromatin remodelling cofactor | 26153137 | Chromatin Remodeler |
| JADE1 | Histone modification write(Histone acetylation) | 24240475;26153137 | Histone Modifier |
| JADE2 | Histone modification write(Histone acetylation) | 24240475;26153137 | Histone Modifier |
| JADE3 | Histone modification write(Histone acetylation) | 24240475;26153137 | Histone Modifier |
| JAK2 | Histone modification write(Histone phosphorylation) | 26153137 | Histone Modifier |
| JARID2 | Histone modification write cofactor(Histone methylation);Essential role in embryonic development, inhibits PRC2 trimethylation of H3K27. | 24063517;24063517;26153137;24240475 | Histone Modifier |
| JDP2 | Chromatin remodelling, Histone modification erase cofactor( Histone acetylation) | 26153137 | Histone Modifier,Chromatin Remodeler |
| JMJD1C | Histone modification erase(Histone methylation);Histone Modification(protein hydroxylases or histone demethylases);H3K9 HDM. | 24063517;26153137;26169266;24240475;22196736 | Histone Modifier |
| JMJD4 |  | 24240475 |  |
| JMJD6 | Histone modification erase(Histone methylation);Histone Modification(protein hydroxylases or histone demethylases);H3R2, H4R3 HDM. Key regulator of haematopoietic differentiation. | 26153137;26169266;24063517;24240475 | Histone Modifier |
| JMJD7 |  | 24240475 |  |
| JMJD8 |  | 24240475 |  |
| KANSL1 | Histone modification write cofactor, Histone modification write cofactor(Histone methylation, Histone acetylation) | 26153137 | Histone Modifier |
| KANSL2 | Histone modification write cofactor(Histone acetylation) | 26153137 | Histone Modifier |
| KANSL3 | Histone modification write cofactor(Histone acetylation) | 26153137 | Histone Modifier |
| KAT2A | Histone modification write(Histone acetylation);Histone Modification(acetylation writer);histone modifiers(acetylation);Writer | 21119629;24063517;26153137;26169266;24240475;24253304 | Histone Modifier |
| KAT2B | Histone modification write(Histone acetylation);Histone Modification(acetylation writer);Writer | 24240475;24253304;24063517;26153137;26169266;24240475 | Histone Modifier |
| KAT5 | Histone modification write(Histone acetylation);Histone Modification(acetylation writer) | 24063517;26153137;26169266;24240475;22196736;22196736 | Histone Modifier |
| KAT6A | Histone modification write(Histone acetylation);Histone Modification(acetylation writer);Component of the MOZ/MORF complex, which has a histone H3 acetyltransferase activity. | 24240475;24063517;26169266;24240475;26153137 | Histone Modifier |
| KAT6B | Histone modification write(Histone acetylation);Histone Modification(acetylation writer) | 24063517;26169266;24240475;26153137 | Histone Modifier |
| KAT7 | Histone modification write(Histone acetylation);Histone Modification(acetylation writer);Responsible for the bulk of histone H4 acetylation in vivo.;Writer | 24063517;26169266;24240475;26153137;24253304 | Histone Modifier |
| KAT8 | Histone modification write(Histone acetylation);Histone Modification(acetylation writer) | 24063517;26169266;24240475;26153137 | Histone Modifier |
| KDM1A | Histone modification erase(Histone methylation);Histone Modification(Histone demethylase (HDM));HDM, also demethylates and stabilizes DNMT1.;Eraser | 24240475;24240475;21915889;22196736;22196736;22196736;24063517;24063517;26153137;26169266;24253304 | Histone Modifier |
| KDM1B | Histone modification erase(Histone methylation);Histone Modification(Histone demethylase (HDM));HDM, required for de novo DNA methylation of a subset of imprinted genes during oogenesis. | 24063517;24063517;26153137;26169266 | Histone Modifier |
| KDM2A | Histone modification erase(Histone methylation);Histone Modification(Histone demethylase (HDM));H3K36me2 HDM. Required to maintain heterochromatic state at centromeres.;Eraser | 24240475;26153137;26169266;24063517;24253304 | Histone Modifier |
| KDM2B | Histone modification erase(Histone methylation);Histone Modification(Histone demethylase (HDM));H3K4me3, H3K36me2 HDM. Represses rRNA genes. | 24240475;24240475;22196736;26153137;26169266;24063517 | Histone Modifier |
| KDM3A | Histone modification erase(Histone methylation);Histone Modification(Histone demethylase (HDM));H3K9me2/me1 HDM. | 24240475;22196736;26153137;26169266;24063517 | Histone Modifier |
| KDM3B | Histone modification erase(Histone methylation);Histone Modification(Histone demethylase (HDM));H3K9 HDM. | 26153137;26169266;24063517 | Histone Modifier |
| KDM4A | Histone modification erase(Histone methylation);Histone Modification(Histone demethylase (HDM));H3K9me3, H3K36me3 HDM.;Eraser | 24240475;22196736;26153137;26169266;24063517;24253304 | Histone Modifier |
| KDM4B | Histone modification erase(Histone methylation);Histone Modification(Histone demethylase (HDM));H3K9me3 HDM. | 24240475;22196736;26153137;26169266;24063517 | Histone Modifier |
| KDM4C | Histone modification erase(Histone methylation);Histone Modification(Histone demethylase (HDM));H3K9me3, H3K36me3 HDM. | 24240475;26153137;26169266;24063517 | Histone Modifier |
| KDM4D | Histone modification erase(Histone methylation);Histone Modification(Histone demethylase (HDM));H3K9me3/me2 HDM. | 24240475;26153137;26169266;24063517 | Histone Modifier |
| KDM4E | Histone modification erase(Histone methylation) | 26153137 | Histone Modifier |
| KDM5A | Histone modification erase(Histone methylation);Histone Modification(Histone demethylase (HDM));H3K4me2/me3 HDM. Prominent role in cell differentiation and senescence (17).;Eraser | 24063517;26153137;26169266;24253304 | Histone Modifier |
| KDM5B | Histone modification erase(Histone methylation);Histone Modification(Histone demethylase (HDM));H3K4me3/me2/me1 HDM.;Eraser | 22196736;24240475;22196736;24063517;26153137;26169266;24253304 | Histone Modifier |
| KDM5C | Histone modification erase(Histone methylation);Histone Modification(Histone demethylase (HDM));H3K4me3/me2 HDM. Participates in the repression of neuronal genes.;Eraser | 24240475;22196736;22196736;24063517;26153137;26169266;24253304 | Histone Modifier |
| KDM5D | Histone modification erase(Histone methylation);Histone Modification(Histone demethylase (HDM));H3K4me3/me2 HDM. | 24063517;26153137;26169266 | Histone Modifier |
| KDM6A | Histone modification erase(Histone methylation);Histone Modification(Histone demethylase (HDM));H3K27me2/me3 HDM. Regulation of HOX gene expression. | 24240475;24063517;26153137;26169266 | Histone Modifier |
| KDM6B | Histone modification erase(Histone methylation);Histone Modification(Histone demethylase (HDM));H3K27me2/me3 HDM. Regulation of HOX gene expression.;Eraser | 24240475;24063517;26153137;26169266;24253304 | Histone Modifier |
| KDM7A | Histone modification erase(Histone methylation) | 26153137;24240475 | Histone Modifier |
| KDM8 | Histone modification erase(Histone methylation);H3K36me2 HDM. Required for G2/M cell cycle progression. | 26153137;24240475;24063517 | Histone Modifier |
| KEAP1 | Chromatin remodelling | 26153137 | Chromatin Remodeler |
| KMT2A | Histone modification write(Histone methylation);Histone Modification(Histone methyltransferases(HMT));H3K4 HMT. Key regulator of development and haematopoiesis. | 22196736;26169266;24063517;24240475;22196736;22196736;26153137 | Histone Modifier |
| KMT2B | Histone modification write(Histone methylation);Histone Modification(Histone methyltransferases(HMT));H3K4 HMT. Required to control the bulk of H3K4me3 during oocyte growth and preimplantation. | 26169266;24063517;24240475;22196736;26153137 | Histone Modifier |
| KMT2C | Histone modification write(Histone methylation);Histone Modification(Histone methyltransferases(HMT));H3K4 HMT. | 26169266;24063517;24240475;26153137 | Histone Modifier |
| KMT2D | Histone modification write(Histone methylation);Histone Modification(Histone methyltransferases(HMT));H3K4 HMT. | 26169266;24063517;24240475;22196736;26153137 | Histone Modifier |
| KMT2E | Histone modification write(Histone methylation);Histone Modification(Histone methyltransferases(HMT));H3K4me1/me2 HMT. Key regulator of haematopoiesis. | 26153137;26169266;24063517;24240475 | Histone Modifier |
| KMT5A | Histone modification write(Histone methylation);Histone Modification(Histone methyltransferases(HMT));Trimethylates H4K20 | 24063517;26153137;26169266;24240475 | Histone Modifier |
| KMT5B | Histone modification write(Histone methylation);Histone Modification(Histone methyltransferases(HMT));H4K20me3 HMT. Key in constitutive heterochromatin formation at pericentormeric regions. | 24063517;26153137;26169266;24240475 | Histone Modifier |
| KMT5C | Histone modification write(Histone methylation);Histone Modification(Histone methyltransferases(HMT));H4K20me3 HMT. Key in constitutive heterochromatin formation at pericentormeric regions. | 24063517;26153137;26169266;24240475 | Histone Modifier |
| L3MBTL1 | Histone modification read | 26153137;24063517;24240475 | Histone Modifier |
| L3MBTL2 | Histone modification read | 26153137;24240475 | Histone Modifier |
| L3MBTL3 | Histone modification read | 26153137;24240475 | Histone Modifier |
| L3MBTL4 | Histone modification read | 26153137;24240475 | Histone Modifier |
| LAS1L | Histone modification write cofactor, Histone modification write cofactor(Histone methylation, Histone acetylation) | 26153137 | Histone Modifier |
| LBR |  | 26153137 |  |
| LEO1 | Histone modification write cofactor(Histone ubiquitination) | 26153137 | Histone Modifier |
| LMNA |  | 24063517 |  |
| LMNB1 | Global heterochromatic changes induced by lamin perturbation are often mirrored by altered levels of chromatin-associated epigenetic histone marks | 24063517 |  |
| LMNB2 |  | 24063517 |  |
| LRWD1 | Chromatin remodelling | 26153137 | Chromatin Remodeler |
| MAP3K7 | Histone modification write | 26153137 | Histone Modifier |
| MAPKAPK3 | Chromatin remodelling | 26153137 | Chromatin Remodeler |
| MASTL | Histone modification write(Histone phosphorylation) | 26153137 | Histone Modifier |
| MAX | Histone modification write cofactor, Histone modification write cofactor, TF, TF(Histone methylation, Histone acetylation, TF activator, TF repressor) | 26153137 | Histone Modifier |
| MAZ | Chromatin remodelling | 26153137 | Chromatin Remodeler |
| MBD1 | Histone modification write cofactor, TF(Histone methylation, TF repressor);DNA modification(DNA methylation reader);Essential for embryonic development. Specifically bind methylated DNA and repress transcription at methylated promoters. | 26153137;26169266;24063517;24240475 | DNA Methylator,Histone Modifier |
| MBD2 | Histone modification write cofactor, Histone modification erase cofactor, TF(Histone methylation, Histone acetylation, TF repressor);DNA modification(DNA methylation reader);Essential for embryonic development. Also bind methylated DNA. | 26153137;26169266;24063517;24063517;24240475;22196736 | DNA Methylator,Histone Modifier |
| MBD3 | Histone modification erase cofactor(Histone acetylation);DNA modification(DNA methylation reader) | 26153137;26169266;24240475 | DNA Methylator,Histone Modifier |
| MBD4 | DNA modification;DNA modification(DNA methylation reader) | 26153137;26169266;24063517;24240475 | DNA Methylator |
| MBD5 | Chromatin remodelling | 26153137;24240475 | Chromatin Remodeler |
| MBD6 | Chromatin remodelling | 26153137;24240475 | Chromatin Remodeler |
| MBIP | Histone modification write cofactor(Histone acetylation) | 26153137 | Histone Modifier |
| MBTD1 | Polycomb group (PcG) protein | 26153137;24240475 |  |
| MCRS1 | Histone modification write(Histone acetylation) | 26153137 | Histone Modifier |
| MDC1 | Histone modification read | 26153137 | Histone Modifier |
| MDM2 |  | 24240475 |  |
| MDM4 |  | 24240475 |  |
| MEAF6 | Histone modification write cofactor(Histone acetylation) | 26153137 | Histone Modifier |
| MECOM | Histone Modification(K9 writer) | 24240475;26169266 | Histone Modifier |
| MECP2 | Histone modification write cofactor, Histone modification write cofactor, TF(Histone methylation, Histone acetylation, TF repressor);DNA modification(DNA methylation reader) | 26153137;26169266;24063517;24240475 | DNA Methylator,Histone Modifier |
| MEN1 | Histone modification write cofactor(Histone methylation);H3K4 HMT. Essential component of a MLL/SET1 HMT complex. Represses telomerase expression. Role in TGFB1-mediated inhibition of cell-proliferation. | 26153137;24063517 | Histone Modifier |
| MGA | Histone modification write cofactor, Histone modification write cofactor, TF, TF(Histone methylation, Histone acetylation, TF activator, TF repressor);Histone Modification(Acetylation, methylation and phosphorylation Reader) | 26153137;26169266 | Histone Modifier |
| MGEA5 | Histone modification write(Histone acetylation) | 26153137;24240475 | Histone Modifier |
| MGMT | DNA modification(DNA methylation editor/eraser) | 26169266 | DNA Methylator |
| MIER1 |  | 24240475 |  |
| MIER2 |  | 24240475 |  |
| MIER3 |  | 24240475 |  |
| MINA | Histone modification erase(Histone methylation) | 26153137 | Histone Modifier |
| MIS18BP1 |  | 24240475 |  |
| MLLT1 | Chromatin remodelling cofactor | 26153137;24240475 | Chromatin Remodeler |
| MLLT10 | Histone modification write cofactor(Histone methylation) | 26153137;24240475 | Histone Modifier |
| MLLT3 |  | 24240475 |  |
| MLLT6 | Histone modification write cofactor(Histone methylation) | 26153137;24240475 | Histone Modifier |
| MOCS1 |  | 24240475 |  |
| MORF4 |  | 24240475 |  |
| MORF4L1 | Histone modification read;Histone Modification(K36 reader) | 26153137;26169266;24240475 | Histone Modifier |
| MORF4L2 | Histone modification erase cofactor(Histone acetylation) | 26153137;24240475 | Histone Modifier |
| MOV10 |  | 26153137 |  |
| MPHOSPH8 | Histone modification read | 26153137 | Histone Modifier |
| MRGBP | Histone modification write cofactor(Histone acetylation) | 24240475;26153137;24240475 | Histone Modifier |
| MSH6 | Histone modification read | 26153137 | Histone Modifier |
| MSL1 | Histone modification write(Histone ubiquitination) | 26153137 | Histone Modifier |
| MSL2 | Histone modification write(Histone ubiquitination) | 26153137 | Histone Modifier |
| MSL3 | Histone modification read | 26153137;24240475 | Histone Modifier |
| MST1 | Histone modification | 26153137;24240475 | Histone Modifier |
| MTA1 | Chromatin remodelling cofactor | 26153137;24063517;24240475 | Chromatin Remodeler |
| MTA2 | Histone modification erase cofactor(Histone acetylation) | 26153137;24063517;24240475 | Histone Modifier |
| MTA3 | Chromatin remodelling cofactor;Maintenance of the normal epithelial architecture through the repression of SNAI1 transcription in a HDAC-dependent manner.;Remodeler | 26153137;24063517;24240475;24253304 | Chromatin Remodeler |
| MTF2 | Polycomb group (PcG) protein;Required for PRC2-mediated Hox repression. | 24063517;26153137;24240475 |  |
| MUM1 | Histone modification read;Opens chromatin to facilitate DNA damage repair | 26153137;24063517 | Histone Modifier |
| MYBBP1A | Chromatin remodelling cofactor | 26153137;22196736 | Chromatin Remodeler |
| MYO1C | Chromatin remodelling cofactor | 26153137 | Chromatin Remodeler |
| MYSM1 | Histone modification erase(Histone ubiquitination) | 26153137 | Histone Modifier |
| NAA60 | Histone modification write(Histone acetylation) | 26153137 | Histone Modifier |
| NAP1L1 | Histone chaperone | 26153137;24240475 | Histone Modifier |
| NAP1L2 | Histone modification cofactor | 26153137;24240475 | Histone Modifier |
| NAP1L3 |  | 24240475 |  |
| NAP1L4 | Histone modification cofactor | 26153137;24240475 | Histone Modifier |
| NAP1L5 |  | 24240475 |  |
| NASP | Chromatin remodelling | 26153137 | Chromatin Remodeler |
| NAT10 | Histone modification write(Histone acetylation) | 26153137;24240475 | Histone Modifier |
| NBN | Chromatin remodelling | 26153137;24240475 | Chromatin Remodeler |
| NCL | Histone chaperone | 26153137 | Histone Modifier |
| NCOA1 | Histone modification write(Histone acetylation);Histone Modification(Histone acetyltransferases) | 26153137;26169266;24240475 | Histone Modifier |
| NCOA2 | Chromatin remodelling cofactor;Histone Modification(Histone acetyltransferases) | 26153137;26169266;24240475 | Histone Modifier,Chromatin Remodeler |
| NCOA3 | Histone modification write(Histone acetylation);Histone Modification(Histone acetyltransferases);HAT activity not studied in detail. | 26153137;26169266;24063517;24240475 | Histone Modifier |
| NCOA4 | Histone Modification(Histone acetyltransferases) | 26169266 | Histone Modifier |
| NCOA5 | Histone Modification(Histone acetyltransferases) | 26169266 | Histone Modifier |
| NCOA6 | Histone modification write cofactor(Histone methylation);Histone Modification(Histone acetyltransferases) | 26153137;26169266 | Histone Modifier |
| NCOA7 | Histone Modification(Histone acetyltransferases) | 26169266 | Histone Modifier |
| NCOR1 | Histone modification erase cofactor(Histone acetylation);Forms complex with HDAC1. | 26153137;24063517 | Histone Modifier |
| NCOR2 | Histone modification erase cofactor(Histone acetylation) | 26153137 | Histone Modifier |
| NEK6 | Histone modification write(Histone phosphorylation) | 26153137 | Histone Modifier |
| NEK9 | Histone modification write(Histone phosphorylation) | 26153137 | Histone Modifier |
| NFRKB | Chromatin remodelling cofactor, TF | 26153137 | Chromatin Remodeler |
| NFYB | Chromatin remodelling, TF( TF activator) | 26153137 | Chromatin Remodeler |
| NFYC | Histone modification | 26153137 | Histone Modifier |
| NIPBL | Histone modification erase cofactor(Histone acetylation);Histone Modification(Acetylation, methylation and phosphorylation Reader) | 26153137;26169266 | Histone Modifier |
| NOC2L | Chromatin remodelling, TF( TF repressor) | 26153137 | Chromatin Remodeler |
| NPAS2 | Chromatin remodelling, TF( TF activator) | 26153137 | Chromatin Remodeler |
| NPM1 | Histone chaperone | 26153137 | Histone Modifier |
| NPM2 | Histone chaperone | 26153137 | Histone Modifier |
| NSD1 | Histone modification write(Histone methylation);Histone Modification(Histone methyltransferases(HMT));H3K36, H4K20 HMT. May influence transcription positively or negatively. | 24063517;26153137;26169266;24240475 | Histone Modifier |
| NSL1 | Histone modification write cofactor(Histone acetylation) | 26153137 | Histone Modifier |
| OGT | Histone modification write(Histone GlcNAcylation) | 26153137 | Histone Modifier |
| ORC1 |  | 24240475 |  |
| ORC2 |  | 24240475 |  |
| PADI1 | Histone modification(Histone citrullination) | 26153137 | Histone Modifier |
| PADI2 | Histone modification(Histone citrullination) | 26153137 | Histone Modifier |
| PADI3 | Histone modification(Histone citrullination) | 26153137 | Histone Modifier |
| PADI4 | Histone modification(Histone citrullination) | 26153137;24240475 | Histone Modifier |
| PAF1 | Histone modification write cofactor(Histone ubiquitination) | 26153137 | Histone Modifier |
| PAGR1 | Histone modification write cofactor, Histone modification write cofactor(Histone methylation, Histone acetylation) | 26153137 | Histone Modifier |
| PAK2 | Histone modification write(Histone phosphorylation) | 26153137 | Histone Modifier |
| PARG | Chromatin remodelling | 26153137 | Chromatin Remodeler |
| PARP1 | Chromatin remodelling | 26153137;24240475 | Chromatin Remodeler |
| PARP2 | Chromatin remodelling cofactor | 26153137;24240475 | Chromatin Remodeler |
| PARP3 | Polycomb group (PcG) protein | 26153137;24240475 |  |
| PARP4 |  | 24240475 |  |
| PATZ1 |  | 24240475 |  |
| PAXIP1 | Histone modification write cofactor(Histone methylation) | 26153137;24240475 | Histone Modifier |
| PBK | Histone modification write(Histone phosphorylation) | 26153137 | Histone Modifier |
| PBRM1 | Histone modification read;Regulator of cell proliferation | 26153137;24063517;24240475 | Histone Modifier |
| PCGF1 | Polycomb group (PcG) protein;Represses CDKN1A expression in a RARE-dependent manner. | 24063517;26153137 |  |
| PCGF2 | Polycomb group (PcG) protein | 24063517;26153137 |  |
| PCGF3 | Polycomb group (PcG) protein | 26153137 |  |
| PCGF5 | Polycomb group (PcG) protein | 26153137 |  |
| PCGF6 | Polycomb group (PcG) protein | 24063517;26153137 |  |
| PCNA | Chromatin remodelling;DNA modification(DNA methylation reader) | 26153137;26169266 | DNA Methylator,Chromatin Remodeler |
| PDP1 | Histone modification read | 26153137 | Histone Modifier |
| PDS5A |  | 24240475 |  |
| PDS5B |  | 24240475 |  |
| PELP1 | Histone modification read, Histone modification write cofactor(Histone methylation, Histone acetylation) | 26153137 | Histone Modifier |
| PES1 |  | 24240475 |  |
| PHC1 | Polycomb group (PcG) protein | 26153137;24063517 |  |
| PHC2 | Polycomb group (PcG) protein | 26153137;24063517 |  |
| PHC3 | Polycomb group (PcG) protein | 26153137;24063517 |  |
| PHF1 | Polycomb group (PcG) protein;Mediates PRC2 intrusion into active H3K36 chromatin regions. | 24240475;24063517;26153137;24240475 | Histone Modifier |
| PHF10 | Chromatin remodelling;Required for the proliferation of neural progenitors | 24063517;26153137;24240475 | Chromatin Remodeler |
| PHF12 | Histone modification erase cofactor(Histone acetylation) | 26153137;24240475 | Histone Modifier |
| PHF13 | Histone modification read | 24240475;26153137;24240475 | Histone Modifier |
| PHF14 | Histone modification read | 26153137;24240475 | Histone Modifier |
| PHF19 | Chromatin remodelling, Histone modification write cofactor( Histone acetylation);Mediates interaction of PRC2 with H3K36me3, essential for full PRC2 activity. | 24240475;24063517;26153137;24240475 | Histone Modifier,Chromatin Remodeler |
| PHF2 | Histone modification erase(Histone methylation);Histone Modification(K4 reader);H3K9me2 HDM. | 24240475;26153137;26169266;24063517;24240475 | Histone Modifier |
| PHF20 | Histone modification write(Histone acetylation) | 26153137;24240475 | Histone Modifier |
| PHF20L1 | Histone modification read | 26153137 | Histone Modifier |
| PHF21A | Histone modification erase cofactor(Histone methylation) | 26153137;24240475 | Histone Modifier |
| PHF21B |  | 24240475 |  |
| PHF23 |  | 24240475 |  |
| PHF3 |  | 24240475 |  |
| PHF6 | Histone Modification(K4 reader) | 26169266;24240475 | Histone Modifier |
| PHF7 |  | 24240475 |  |
| PHF8 | Histone modification erase(Histone methylation);Histone Modification(K4 reader);H3K9me1/me2, H3K27me2, H4K20me1 HDM. Key role in cell cycle progression.;Eraser | 24240475;26153137;26169266;24063517;24240475;24253304;24253304;22196736 | Histone Modifier |
| PHIP | Histone modification read | 26153137;24240475 | Histone Modifier |
| PIWIL4 | Chromatin remodelling, Histone modification erase cofactor( Histone methylation) | 26153137 | Histone Modifier,Chromatin Remodeler |
| PKM | Histone modification write cofactor(Histone phosphorylation) | 26153137 | Histone Modifier |
| PKN1 | Histone modification write(Histone phosphorylation) | 26153137 | Histone Modifier |
| POGZ | Histone modification read(Histone methylation);Histone Modification(Acetylation, methylation and phosphorylation Reader) | 26153137;26169266 | Histone Modifier |
| POLE3 | Histone chaperone | 26153137;24063517 | Histone Modifier |
| PPARGC1A | Histone modification cofactor | 26153137 | Histone Modifier |
| PPM1G | Chromatin remodelling | 26153137 | Chromatin Remodeler |
| PPP2CA | Histone modification write(Histone phosphorylation) | 26153137 | Histone Modifier |
| PPP4C | Histone modification erase(Histone phosphorylation) | 26153137 | Histone Modifier |
| PPP4R2 | Histone modification cofactor | 26153137 | Histone Modifier |
| PPP4R3A | Histone modification erase cofactor(Histone phosphorylation);Histone Modification(Phosphorylation editor) | 26153137;26169266 | Histone Modifier |
| PPP4R3B | Histone modification erase cofactor(Histone phosphorylation);Histone Modification(Phosphorylation editor) | 26153137;26169266 | Histone Modifier |
| PPP4R3CP | Histone Modification(Phosphorylation editor) | 26169266 | Histone Modifier |
| PRC1 |  | 21915889 |  |
| PRDM1 | Histone modification write cofactor(Histone methylation);Histone Modification(Histone methyltransferases) | 26153137;26169266;24240475 | Histone Modifier |
| PRDM10 | Histone Modification(Histone methyltransferases) | 26169266 | Histone Modifier |
| PRDM11 | Histone modification write(Histone methylation);Histone Modification(Histone methyltransferases) | 26153137;26169266;24240475 | Histone Modifier |
| PRDM12 | Histone modification write cofactor(Histone methylation);Histone Modification(Histone methyltransferases) | 26153137;26169266;24240475 | Histone Modifier |
| PRDM13 | Histone modification write(Histone methylation);Histone Modification(Histone methyltransferases) | 26153137;26169266;24240475 | Histone Modifier |
| PRDM14 | DNA modification(DNA demethylation);Histone Modification(Histone methyltransferases) | 26153137;26169266;24240475 | DNA Methylator,Histone Modifier |
| PRDM15 | Histone Modification(Histone methyltransferases) | 26169266;24240475 | Histone Modifier |
| PRDM16 | Histone modification write cofactor, TF(Histone methylation, TF repressor);Histone Modification(Histone methyltransferases) | 26153137;26169266;24240475 | Histone Modifier |
| PRDM2 | Histone modification write(Histone methylation);Histone Modification(Histone methyltransferases);H3K9 HMT | 24063517;26153137;26169266;24240475 | Histone Modifier |
| PRDM4 | Histone modification write(Histone methylation);Histone Modification(Histone methyltransferases) | 26153137;26169266;24240475 | Histone Modifier |
| PRDM5 | Histone modification write(Histone methylation);Histone Modification(Histone methyltransferases) | 26153137;26169266;24240475 | Histone Modifier |
| PRDM6 | Histone modification write(Histone methylation);Histone Modification(Histone methyltransferases) | 26153137;26169266;24240475 | Histone Modifier |
| PRDM7 | Histone modification write(Histone methylation);Histone Modification(Histone methyltransferases) | 26153137;26169266;24240475 | Histone Modifier |
| PRDM8 | Histone modification write(Histone methylation);Histone Modification(Histone methyltransferases) | 26153137;26169266;24240475 | Histone Modifier |
| PRDM9 | Histone modification write(Histone methylation);Histone Modification(Histone methyltransferases);H3K4me3 HMT. Essential for meiotic progression | 26153137;26169266;24063517;24240475 | Histone Modifier |
| PRKAA1 | Histone modification write(Histone phosphorylation) | 26153137 | Histone Modifier |
| PRKAA2 | Histone modification write(Histone phosphorylation) | 26153137 | Histone Modifier |
| PRKAB1 | Histone modification write cofactor(Histone phosphorylation) | 26153137 | Histone Modifier |
| PRKAB2 | Histone modification write cofactor(Histone phosphorylation) | 26153137 | Histone Modifier |
| PRKAG1 | Histone modification write cofactor(Histone phosphorylation) | 26153137 | Histone Modifier |
| PRKAG2 | Histone modification write cofactor(Histone phosphorylation) | 26153137 | Histone Modifier |
| PRKAG3 | Histone modification write cofactor(Histone phosphorylation) | 26153137 | Histone Modifier |
| PRKCA | Histone modification write cofactor(Histone phosphorylation) | 26153137 | Histone Modifier |
| PRKCB | Histone modification write(Histone methylation) | 26153137 | Histone Modifier |
| PRKCD | Histone modification | 26153137 | Histone Modifier |
| PRKDC | Histone modification write(Histone phosphorylation) | 26153137 | Histone Modifier |
| PRMT1 | Histone modification write(Histone methylation);Histone Modification(Histone methyltransferases) | 26153137;26169266;24240475 | Histone Modifier |
| PRMT2 | Histone modification write(Histone methylation);Histone Modification(Histone methyltransferases) | 26153137;26169266;24240475 | Histone Modifier |
| PRMT3 | Histone Modification(Histone methyltransferases) | 26169266 | Histone Modifier |
| PRMT5 | Histone modification write(Histone methylation);Histone Modification(Histone methyltransferases) | 26153137;26169266;24240475 | Histone Modifier |
| PRMT6 | Histone modification write(Histone methylation);Histone Modification(Histone methyltransferases) | 26153137;26169266;24240475 | Histone Modifier |
| PRMT7 | Histone modification write(Histone methylation);Histone Modification(Histone methyltransferases) | 26153137;26169266;24240475 | Histone Modifier |
| PRMT8 | Histone modification write(Histone methylation);Histone Modification(Histone methyltransferases) | 26153137;26169266;24240475 | Histone Modifier |
| PRMT9 | Histone modification write(Histone methylation);Histone Modification(Histone methyltransferases) | 26153137;24240475;26169266;24240475 | Histone Modifier |
| PRPF31 | Histone modification write cofactor, Histone modification write cofactor(Histone methylation, Histone acetylation) | 26153137 | Histone Modifier |
| PRR12 |  | 24240475 |  |
| PRR14 | Histone modification write(Histone phosphorylation) | 26153137 | Histone Modifier |
| PSIP1 | Chromatin remodelling | 24240475;26153137;24240475 | Chromatin Remodeler |
| PWWP2B |  | 24240475 |  |
| PYGO1 |  | 24240475 |  |
| PYGO2 |  | 24240475 |  |
| RAC3 | Writer | 24253304 |  |
| RAD51 | Histone modification erase(Histone ubiquitination) | 26153137 | Histone Modifier |
| RAD54B | Chromatin remodelling | 26153137 | Chromatin Remodeler |
| RAD54L | Chromatin remodelling;Nucleosome Positioning and Remodeling(Chromatin remodelling helicase) | 26153137;26169266;24240475 | Chromatin Remodeler |
| RAD54L2 | Chromatin remodelling | 26153137 | Chromatin Remodeler |
| RAG1 | Histone modification write(Histone ubiquitination) | 26153137 | Histone Modifier |
| RAG2 | Histone modification read;Histone Modification(K4 reader);Reader | 26153137;26169266;24253304 | Histone Modifier |
| RAI1 | Chromatin remodelling | 26153137 | Chromatin Remodeler |
| RARA | Histone modification write cofactor, TF, TF(Histone methylation, TF activator, TF repressor) | 26153137 | Histone Modifier |
| RB1 | Chromatin remodelling, Histone modification write( Histone ubiquitination) | 22196736;26153137 | Histone Modifier,Chromatin Remodeler |
| RBBP4 | Histone chaperone;Also part of PRC2 complex. | 24063517;24063517;24063517;26153137;24240475 | Histone Modifier |
| RBBP5 | Histone modification write cofactor(Histone methylation);Complex with MLL | 22196736;22196736;22196736;26153137;24063517;24240475 | Histone Modifier |
| RBBP7 | Histone chaperone;Also part of PRC2 complex. | 24063517;24063517;24063517;26153137;24240475 | Histone Modifier |
| RBP1 |  | 22196736 |  |
| RBX1 | Histone modification write cofactor(Histone ubiquitination) | 22196736;26153137;24240475 | Histone Modifier |
| RCC1 | Chromatin remodelling | 26153137 | Chromatin Remodeler |
| RCOR1 | Histone modification erase cofactor, Histone modification erase cofactor(Histone acetylation, Histone methylation) | 26153137;24240475 | Histone Modifier |
| RCOR2 |  | 24240475 |  |
| RCOR3 | Histone modification erase cofactor(Histone acetylation) | 26153137;24240475 | Histone Modifier |
| REST | Histone modification erase cofactor, TF, TF(Histone acetylation, TF activator, TF repressor) | 26153137 | Histone Modifier |
| REV1 |  | 24240475 |  |
| RING1 | Histone modification write, Polycomb group (PcG) protein(Histone ubiquitination);H2AK119ub. | 26153137;24063517 | Histone Modifier |
| RIT1 |  | 22196736 |  |
| RLIM | Histone modification erase cofactor(Histone acetylation) | 26153137 | Histone Modifier |
| RMI1 | DNA modification | 26153137 | DNA Methylator |
| RNF168 | Histone modification write(Histone ubiquitination) | 26153137 | Histone Modifier |
| RNF17 |  | 24240475 |  |
| RNF2 | Histone modification write(Histone ubiquitination);H2AK119ub. Acts as the main ub ligase in PRC1. | 24240475;22196736;22196736;24063517;26153137 | Histone Modifier |
| RNF20 | Histone modification write(Histone ubiquitination);ubiquitination | 21119629;24240475;26153137;24240475;22196736 | Histone Modifier |
| RNF40 | Histone modification write cofactor(Histone ubiquitination) | 24240475;26153137;24240475;22196736 | Histone Modifier |
| RNF8 | Histone modification write(Histone ubiquitination) | 26153137 | Histone Modifier |
| RPS6KA3 | Histone modification write cofactor(Histone phosphorylation) | 26153137;24240475 | Histone Modifier |
| RPS6KA4 | Histone modification write(Histone phosphorylation) | 26153137;24240475 | Histone Modifier |
| RPS6KA5 | Histone modification write(Histone phosphorylation) | 26153137;24240475 | Histone Modifier |
| RRP8 | Histone modification cofactor | 26153137 | Histone Modifier |
| RSAD1 |  | 24240475 |  |
| RSF1 | Histone modification read | 26153137;24063517;24240475 | Histone Modifier |
| RTF1 | Required for H3K4me3 HMT on stem cell pluripotency genes. | 24063517 | Histone Modifier |
| RUVBL1 | Chromatin remodelling, Histone modification write( Histone phosphorylation) | 26153137;24240475 | Histone Modifier,Chromatin Remodeler |
| RUVBL2 | Chromatin remodelling cofactor | 26153137;24240475 | Chromatin Remodeler |
| RYBP | Polycomb group (PcG) protein | 26153137 |  |
| SAFB | Chromatin remodelling | 26153137 | Chromatin Remodeler |
| SAP130 | Histone modification erase cofactor(Histone acetylation) | 26153137 | Histone Modifier |
| SAP18 | Histone modification erase cofactor(Histone acetylation) | 26153137 | Histone Modifier |
| SAP25 | Histone modification erase cofactor(Histone acetylation) | 26153137 | Histone Modifier |
| SAP30 | Histone modification erase cofactor(Histone acetylation) | 26153137;22196736;22196736 | Histone Modifier |
| SAP30L | Histone modification erase cofactor(Histone acetylation) | 26153137 | Histone Modifier |
| SATB1 | Chromatin remodelling cofactor | 26153137;24240475 | Chromatin Remodeler |
| SATB2 | Chromatin remodelling cofactor | 26153137;24240475 | Chromatin Remodeler |
| SCMH1 | Polycomb group (PcG) protein | 24240475;26153137 |  |
| SCML1 |  | 24240475 |  |
| SCML2 | Polycomb group (PcG) protein | 26153137;24240475 |  |
| SCML4 | Polycomb group (PcG) protein | 26153137;24240475 |  |
| SENP1 | Histone modification erase cofactor(Histone sumoylation) | 26153137 | Histone Modifier |
| SENP3 | Histone modification erase, Histone modification write cofactor(Histone sumoylation, Histone acetylation) | 26153137 | Histone Modifier |
| SET | Histone modification;Histone Modification(histone acetylases (HAT));Promotes apoptosis. Inhibits p300/CBP and PCAF-mediated acetyltransferase. | 26153137;26169266;24063517;24240475 | Histone Modifier |
| SETBP1 | Histone Modification(Histone methyltransferases(HMT)) | 26169266;24240475 | Histone Modifier |
| SETD1A | Histone modification write(Histone methylation);Histone Modification(Histone methyltransferases(HMT));methylation;H3K4 HMT | 21119629;24063517;26153137;26169266;24240475 | Histone Modifier |
| SETD1B | Histone modification write(Histone methylation);Histone Modification(Histone methyltransferases(HMT));H3K4 HMT. | 22196736;24063517;26153137;26169266;24240475 | Histone Modifier |
| SETD2 | Histone modification write(Histone methylation);Histone Modification(Histone methyltransferases(HMT));H3K36 HMT. | 24063517;26153137;26169266;24240475 | Histone Modifier |
| SETD3 | Histone modification write(Histone methylation);Histone Modification(Histone methyltransferases(HMT)) | 26153137;26169266;24240475 | Histone Modifier |
| SETD4 | Histone Modification(Histone methyltransferases(HMT)) | 26169266;24240475 | Histone Modifier |
| SETD5 | Histone modification write(Histone methylation);Histone Modification(Histone methyltransferases(HMT)) | 26153137;26169266;24240475 | Histone Modifier |
| SETD6 | Chromatin remodelling, Histone modification write( Histone methylation);Histone Modification(Histone methyltransferases(HMT)) | 26153137;26169266;24240475 | Histone Modifier,Chromatin Remodeler |
| SETD7 | Histone modification write(Histone methylation);Histone Modification(Histone methyltransferases(HMT));H3K4 HMT | 22196736;24063517;26153137;26169266;24240475 | Histone Modifier |
| SETD8P1 |  | 24240475 |  |
| SETD9 | Histone Modification(Histone methyltransferases(HMT)) | 26169266 | Histone Modifier |
| SETDB1 | Histone modification write(Histone methylation);Histone Modification(Histone methyltransferases(HMT));H3K9 HMT.;Writer | 22196736;24063517;26153137;26169266;24240475;24253304;22196736 | Histone Modifier |
| SETDB2 | Histone modification write(Histone methylation);Histone Modification(Histone methyltransferases(HMT));H3K9 HMT. | 26153137;26169266;24063517;24240475 | Histone Modifier |
| SETMAR | Histone modification write(Histone methylation);Histone Modification(Histone methyltransferases(HMT)) | 26153137;26169266;24240475 | Histone Modifier |
| SF3B1 | RNA modification | 26153137;22196736 |  |
| SF3B3 | RNA modification | 26153137 |  |
| SFMBT1 | Polycomb group (PcG) protein | 26153137;24240475 |  |
| SFMBT2 | Histone modification read, Polycomb group (PcG) protein, TF( TF repressor) | 26153137;24240475 | Histone Modifier |
| SFPQ | Chromatin remodelling cofactor, RNA modification, TF( TF repressor) | 22196736;26153137 | Chromatin Remodeler |
| SGF29 | Histone modification read | 26153137 | Histone Modifier |
| SHPRH | Histone modification write cofactor(Histone ubiquitination) | 26153137;24240475 | Histone Modifier |
| SIN3A | Histone modification erase cofactor, TF, TF(Histone acetylation, TF activator, TF repressor) | 26153137;24240475 | Histone Modifier |
| SIN3B | Histone modification erase cofactor, TF(Histone acetylation, TF repressor) | 26153137;24240475 | Histone Modifier |
| SIRT1 | Histone modification erase, Histone modification write cofactor(Histone acetylation, Histone methylation);Histone Modification(acetylation editor);Interacts with PRC2, non-histone deacetylase activity. Involved in normal ageing through resistance to cellular stress. Deacetylates p53. Located in nucleus and cytoplasm;Eraser | 26153137;26169266;24063517;24063517;24240475;24253304;22196736 | Histone Modifier |
| SIRT2 | Histone modification erase, Histone modification write cofactor(Histone acetylation, Histone methylation);Histone Modification(acetylation editor);Deacetylates alpha-tubulin. Located in the cytoplasm | 26153137;26169266;24063517;24240475 | Histone Modifier |
| SIRT3 | Histone Modification(acetylation editor);Located in the mitochondria | 26169266;24063517 | Histone Modifier |
| SIRT4 | Histone Modification(acetylation editor) | 26169266;24063517 | Histone Modifier |
| SIRT5 | Histone Modification(acetylation editor) | 26169266;24063517 | Histone Modifier |
| SIRT6 | Histone modification erase(Histone acetylation);Histone Modification(acetylation editor);Located in the nucleus. H3K9 and H3K56 deacetylase activity.;Eraser | 26153137;26169266;24063517;24240475;24253304;22196736;22196736;22196736 | Histone Modifier |
| SIRT7 | Histone modification erase(Histone acetylation);Histone Modification(acetylation editor);Located in the nucleus. | 26153137;26169266;24063517;24240475 | Histone Modifier |
| SKP1 | Histone modification write cofactor(Histone ubiquitination) | 26153137 | Histone Modifier |
| SLF1 | Histone modification read | 26153137 | Histone Modifier |
| SMARCA1 | Chromatin remodelling, Histone modification erase( Histone acetylation);Nucleosome Positioning and Remodeling(Chromatin remodelling helicase) | 24063517;26153137;26169266;24240475 | Histone Modifier,Chromatin Remodeler |
| SMARCA2 | Histone modification read, TF( TF activator);Catalytic component of SWI/SNIF complex | 24240475;24063517;26153137;24240475 | Histone Modifier |
| SMARCA4 | Histone modification read, TF( TF activator);Nucleosome Positioning and Remodeling(Chromatin remodelling helicase);Essential for the maintenance of multipotent neural stem cells;Remodeler | 24240475;22196736;24063517;26153137;26169266;24253304 | Histone Modifier,Chromatin Remodeler |
| SMARCA5 | Chromatin remodelling;Nucleosome Positioning and Remodeling(Chromatin remodelling helicase);Required for replication of pericentric heterochromatin in S-phase specifically in conjunction with BAZ1A | 24063517;26153137;26169266;24240475 | Chromatin Remodeler |
| SMARCAD1 | Chromatin remodelling | 26153137;24240475 | Chromatin Remodeler |
| SMARCAL1 | Chromatin remodelling | 26153137 | Chromatin Remodeler |
| SMARCB1 | Histone modification read;Nucleosome Positioning and Remodeling(Chromatin remodelling helicase);Remodeler | 24240475;24063517;26153137;26169266;24240475;24253304 | Histone Modifier,Chromatin Remodeler |
| SMARCC1 | Chromatin remodelling cofactor;Remodeler | 26153137;24063517;24240475;24253304 | Chromatin Remodeler |
| SMARCC2 | Chromatin remodelling cofactor;Remodeler | 24240475;26153137;24063517;24240475;24253304 | Chromatin Remodeler |
| SMARCD1 | Chromatin remodelling | 26153137;24063517;24240475 | Chromatin Remodeler |
| SMARCD2 | Chromatin remodelling cofactor | 24240475;26153137;24063517;24240475 | Chromatin Remodeler |
| SMARCD3 | Chromatin remodelling cofactor | 26153137;24063517;24240475 | Chromatin Remodeler |
| SMARCE1 | Chromatin remodelling cofactor | 24240475;24063517;26153137;24240475 | Chromatin Remodeler |
| SMC1A | Histone Modification(Acetylation, methylation and phosphorylation Reader) | 26169266 | Histone Modifier |
| SMCHD1 | Histone Modification(Acetylation, methylation and phosphorylation Reader) | 26169266 | Histone Modifier |
| SMYD1 | Histone modification write(Histone methylation);Histone Modification(Histone methyltransferases(HMT));H3K4 HMT | 26153137;26169266;24063517;24240475 | Histone Modifier |
| SMYD2 | Histone modification write(Histone methylation);Histone Modification(Histone methyltransferases(HMT));H3K4me, H3K36me2 HMT. Also methylates TP53 and RB1 | 24063517;26153137;26169266;24240475 | Histone Modifier |
| SMYD3 | Histone modification write(Histone methylation);Histone Modification(Histone methyltransferases(HMT));H3K4me2/me3 HMT | 26153137;26169266;24063517;24240475 | Histone Modifier |
| SMYD4 | Histone modification erase cofactor(Histone acetylation);Histone Modification(Histone methyltransferases(HMT)) | 26153137;26169266;24240475 | Histone Modifier |
| SMYD5 | Histone Modification(Histone methyltransferases(HMT)) | 26169266;24240475 | Histone Modifier |
| SNAI2 | Histone modification erase cofactor | 26153137 | Histone Modifier |
| SND1 |  | 24240475 |  |
| SP1 | Chromatin remodelling, TF, TF( TF activator, TF repressor) | 26153137;22196736 | Chromatin Remodeler |
| SP100 | Chromatin remodelling cofactor | 26153137;24240475 | Chromatin Remodeler |
| SP110 |  | 24240475 |  |
| SP140 | Histone modification read, TF | 26153137;24240475 | Histone Modifier |
| SPEN | Histone modification erase cofactor, TF, TF(Histone acetylation, TF activator, TF repressor) | 26153137 | Histone Modifier |
| SPOP | Histone modification write(Histone ubiquitination) | 26153137 | Histone Modifier |
| SRCAP | Chromatin remodelling, Histone modification erase( Histone acetylation);Nucleosome Positioning and Remodeling(Chromatin remodelling helicase) | 26153137;26169266 | Histone Modifier,Chromatin Remodeler |
| SRRM2 |  | 24240475 |  |
| SRSF1 | RNA modification | 26153137 |  |
| SRSF3 | RNA modification | 26153137 |  |
| SS18L1 | Chromatin remodelling | 26153137 | Chromatin Remodeler |
| SS18L2 | Chromatin remodelling | 26153137 | Chromatin Remodeler |
| SSRP1 | Chromatin remodelling | 26153137;24240475 | Chromatin Remodeler |
| STK31 |  | 24240475 |  |
| STK4 | Histone modification write(Histone phosphorylation) | 26153137 | Histone Modifier |
| SUDS3 | Histone modification erase cofactor(Histone acetylation) | 26153137 | Histone Modifier |
| SUPT16H | Histone modification read | 26153137 | Histone Modifier |
| SUPT3H | Histone modification write cofactor(Histone acetylation) | 26153137 | Histone Modifier |
| SUPT6H | Histone modification erase cofactor(Histone methylation) | 26153137 | Histone Modifier |
| SUPT7L | Histone chaperone | 26153137 | Histone Modifier |
| SUV39H1 | Histone modification write, Histone modification write(Histone methylation, Histone phosphorylation);Histone Modification(Histone methyltransferases(HMT));H3K9me3 HMT, uses H3K9me1 as substrate | 24063517;26153137;26169266;24240475;22196736;22196736 | Histone Modifier |
| SUV39H2 | Histone modification write(Histone methylation);Histone Modification(Histone methyltransferases(HMT));H3K9me3 HMT, uses H3K9me1 as substrate | 24063517;26153137;26169266 | Histone Modifier |
| SUZ12 | Histone modification write cofactor, Histone modification write cofactor, Polycomb group (PcG) protein, TF(Histone methylation, Histone ubiquitination, TF repressor);Histone Modification(chromatin silencing);Required for PRC2 H3K27 HMT activity. Interacts with SIRT1. | 26153137;26169266;24063517;24240475;22196736;22196736;22196736 | Histone Modifier |
| SYNCRIP | RNA modification(mRNA editing) | 26153137 |  |
| TADA1 | Histone chaperone | 26153137 | Histone Modifier |
| TADA2A | Histone modification read, TF( TF activator) | 26153137 | Histone Modifier |
| TADA2B | Histone modification write cofactor(Histone acetylation) | 26153137 | Histone Modifier |
| TADA3 | Histone modification write cofactor(Histone acetylation) | 26153137 | Histone Modifier |
| TAF1 | Histone modification write(Histone acetylation);Histone Modification(Acetylation, methylation and phosphorylation) | 26153137;26169266;24240475 | Histone Modifier |
| TAF10 | Histone chaperone, Histone modification write( Histone acetylation) | 26153137 | Histone Modifier |
| TAF12 | Histone chaperone, Histone modification write( Histone acetylation) | 26153137 | Histone Modifier |
| TAF1L | Histone modification read | 26153137;24240475 | Histone Modifier |
| TAF2 | TF | 26153137 |  |
| TAF3 | Histone modification read;Histone Modification(K4 reader);Reader | 26153137;26169266;24253304 | Histone Modifier |
| TAF4 | Histone chaperone | 26153137 | Histone Modifier |
| TAF5 | Histone modification write cofactor(Histone acetylation) | 26153137 | Histone Modifier |
| TAF5L | Histone modification write cofactor(Histone acetylation) | 26153137 | Histone Modifier |
| TAF6 | Histone chaperone | 26153137 | Histone Modifier |
| TAF6L | Histone chaperone | 26153137 | Histone Modifier |
| TAF7 | Histone modification write cofactor, Histone modification write cofactor(Histone methylation, Histone acetylation) | 26153137 | Histone Modifier |
| TAF8 | Histone chaperone | 26153137 | Histone Modifier |
| TAF9 | Histone chaperone | 26153137 | Histone Modifier |
| TAF9B | Histone chaperone | 26153137 | Histone Modifier |
| TBL1XR1 | Associates with HDAC3 | 26153137;24063517 |  |
| TCF4 |  | 22196736 |  |
| TDG | DNA modification(DNA hydroxymethylation);DNA modification(DNA methylation editor/eraser);Essential for DNA demethylation | 26153137;26169266;24063517 | DNA Methylator |
| TDRD1 |  | 24240475 |  |
| TDRD12 |  | 24240475 |  |
| TDRD3 | Histone modification read | 26153137;24240475 | Histone Modifier |
| TDRD5 |  | 24240475 |  |
| TDRD7 | Histone modification read | 26153137;24240475 | Histone Modifier |
| TDRD9 |  | 24240475 |  |
| TDRKH | RNA modification | 26153137;24240475 |  |
| TERF1 |  | 24240475 |  |
| TERF2 |  | 24240475 |  |
| TET1 | DNA modification(DNA hydroxymethylation);DNA modification(DNA methylation editor/eraser);Putative role in DNA demethylation | 26153137;26169266;24063517;22196736 | DNA Methylator |
| TET2 | DNA modification(DNA hydroxymethylation);DNA modification(DNA methylation editor/eraser);Putative role in DNA demethylation | 26153137;26169266;24063517;24240475 | DNA Methylator |
| TET3 | DNA modification(DNA hydroxymethylation);DNA modification(DNA methylation editor/eraser) | 26153137;26169266 | DNA Methylator |
| TEX10 | Histone modification write cofactor, Histone modification write cofactor(Histone methylation, Histone acetylation) | 26153137 | Histone Modifier |
| TFDP1 | Histone modification | 26153137 | Histone Modifier |
| TFF1 |  | 22196736 |  |
| TFPT | Chromatin remodelling cofactor, DNA modification( DNA hydroxymethylation);Putative regulatory component of the INO80 complex | 26153137;24063517 | DNA Methylator,Chromatin Remodeler |
| TLE1 | Chromatin remodelling, Histone modification cofactor | 26153137 | Histone Modifier,Chromatin Remodeler |
| TLE2 | Histone modification cofactor | 26153137 | Histone Modifier |
| TLE4 | Histone modification erase cofactor, TF | 26153137 | Histone Modifier |
| TLK1 | Histone modification write(Histone phosphorylation) | 26153137 | Histone Modifier |
| TLK2 | Histone modification write(Histone phosphorylation) | 26153137 | Histone Modifier |
| TNP1 | Chromatin remodelling | 26153137 | Chromatin Remodeler |
| TNP2 | Chromatin remodelling | 26153137 | Chromatin Remodeler |
| TONSL | Chromatin remodelling | 26153137 | Chromatin Remodeler |
| TOP2A | Chromatin remodelling | 26153137 | Chromatin Remodeler |
| TOP2B | Chromatin remodelling | 26153137 | Chromatin Remodeler |
| TOPBP1 |  | 24240475 |  |
| TOX |  | 24240475 |  |
| TOX2 |  | 24240475 |  |
| TOX3 |  | 24240475 |  |
| TOX4 |  | 24240475 |  |
| TP53 | Histone modification write cofactor, TF, TF(Histone acetylation, TF activator, TF repressor) | 26153137 | Histone Modifier |
| TP53BP1 | Histone modification read;Histone Modification(K79 reader) | 26153137;26169266;24240475 | Histone Modifier |
| TRDMT1 |  | 24240475 |  |
| TRIM16 | Histone modification write(Histone acetylation) | 26153137 | Histone Modifier |
| TRIM24 | Histone modification read | 26153137;24240475 | Histone Modifier |
| TRIM27 | Histone modification erase cofactor(Histone acetylation) | 26153137 | Histone Modifier |
| TRIM28 | Histone modification read;Mediates silencing by recruiting SET1 H3K9me3 HMT and HDAC NuRD complex. Mediates apoptosis through degradation of p53 | 26153137;24063517;24063517 | Histone Modifier |
| TRIM33 | Histone modification read | 26153137 | Histone Modifier |
| TRRAP | Histone modification write cofactor(Histone acetylation);Histone Modification(Acetylation, methylation and phosphorylation Reader) | 26153137;26169266;24240475;22196736 | Histone Modifier |
| TSPY1 |  | 24240475 |  |
| TSPYL1 |  | 24240475 |  |
| TSPYL2 |  | 24240475 |  |
| TSPYL4 |  | 24240475 |  |
| TSPYL5 |  | 24240475 |  |
| TSPYL6 |  | 24240475 |  |
| TSSK6 | Histone modification write(Histone phosphorylation) | 26153137 | Histone Modifier |
| TTF2 | Nucleosome Positioning and Remodeling(Chromatin remodelling helicase) | 26169266 | Chromatin Remodeler |
| TTK | Histone modification write cofactor(Histone phosphorylation) | 26153137 | Histone Modifier |
| TXN2 |  | 22196736 |  |
| TYW5 | RNA modification | 26153137 |  |
| UBE2A | Histone modification write(Histone ubiquitination) | 26153137;24240475 | Histone Modifier |
| UBE2B | Histone modification write(Histone ubiquitination) | 26153137;24240475 | Histone Modifier |
| UBE2D1 | Histone modification write(Histone ubiquitination) | 26153137 | Histone Modifier |
| UBE2D3 | Histone modification write(Histone ubiquitination) | 26153137 | Histone Modifier |
| UBE2E1 | Histone modification write(Histone ubiquitination) | 26153137 | Histone Modifier |
| UBE2H | Histone modification write(Histone ubiquitination) | 26153137 | Histone Modifier |
| UBE2N | Histone modification write(Histone ubiquitination) | 26153137 | Histone Modifier |
| UBE2T | Histone modification write(Histone ubiquitination) | 26153137 | Histone Modifier |
| UBN1 | Histone modification write cofactor(Histone methylation) | 26153137 | Histone Modifier |
| UBR2 | Histone modification write(Histone ubiquitination) | 26153137 | Histone Modifier |
| UBR5 | Chromatin remodelling, Histone modification write cofactor( Histone ubiquitination) | 26153137 | Histone Modifier,Chromatin Remodeler |
| UBR7 | DNA modification cofactor(DNA methylation) | 26153137 | DNA Methylator |
| UBTF |  | 24240475 |  |
| UCHL5 | Histone modification erase cofactor(Histone ubiquitination) | 26153137 | Histone Modifier |
| UHRF1 | Histone modification read, Histone modification write cofactor(Histone ubiquitination);DNA modification(DNA methylation reader) | 26153137;26169266;24240475 | DNA Methylator,Histone Modifier |
| UHRF2 | Histone modification read | 26153137;24240475 | Histone Modifier |
| UIMC1 | Histone modification read | 26153137 | Histone Modifier |
| UNK |  | 24240475 |  |
| USP11 | Histone modification erase cofactor(Histone ubiquitination) | 26153137 | Histone Modifier |
| USP12 | Histone modification erase(Histone ubiquitination) | 26153137 | Histone Modifier |
| USP15 | Histone modification erase(Histone ubiquitination) | 26153137 | Histone Modifier |
| USP16 | Histone modification erase(Histone ubiquitination) | 26153137 | Histone Modifier |
| USP17L2 | Histone modification erase cofactor(Histone ubiquitination) | 26153137 | Histone Modifier |
| USP21 | Histone modification erase(Histone ubiquitination) | 26153137 | Histone Modifier |
| USP22 | Histone modification write cofactor(Histone ubiquitination) | 26153137 | Histone Modifier |
| USP3 | Histone modification write(Histone ubiquitination) | 26153137 | Histone Modifier |
| USP36 | Histone modification write cofactor(Histone ubiquitination) | 26153137 | Histone Modifier |
| USP44 | Histone modification erase(Histone ubiquitination) | 26153137 | Histone Modifier |
| USP46 | Histone modification erase(Histone ubiquitination) | 26153137 | Histone Modifier |
| USP49 | Histone modification erase(Histone ubiquitination) | 26153137 | Histone Modifier |
| USP7 | Histone modification erase, DNA modification cofactor(Histone ubiquitination, DNA methylation) | 26153137 | DNA Methylator,Histone Modifier |
| UTY | Histone modification erase(Histone ubiquitination);Histone Modification(Histone demethylase (HDM));H3K27me3/me2/me1 HDM. | 26153137;26169266;24063517 | Histone Modifier |
| VDR | Chromatin remodelling cofactor, TF | 26153137 | Chromatin Remodeler |
| VEZF1 |  | 24240475 |  |
| VPS72 | Histone modification write cofactor(Histone acetylation) | 26153137 | Histone Modifier |
| VRK1 | Histone modification write(Histone phosphorylation) | 26153137 | Histone Modifier |
| WAC | Histone modification write cofactor(Histone ubiquitination) | 26153137 | Histone Modifier |
| WDR5 | Histone modification read;Complex with MLL.;Writer | 26153137;24063517;24240475;24253304;24253304;22196736;22196736 | Histone Modifier |
| WDR77 | Histone modification write(Histone methylation) | 26153137;22196736 | Histone Modifier |
| WDR82 | Histone modification write cofactor(Histone methylation) | 26153137;24240475 | Histone Modifier |
| WHSC1 | Histone modification write(Histone methylation);Histone Modification(K36 writer);Writer | 22196736;26153137;26169266;24240475;24253304 | Histone Modifier |
| WHSC1L1 | Chromatin remodelling cofactor, TF | 26153137;24240475 | Chromatin Remodeler |
| WSB2 | Histone modification write(Histone ubiquitination) | 26153137 | Histone Modifier |
| XRCC1 |  | 24240475 |  |
| YAF2 | Chromatin remodelling cofactor | 26153137 | Chromatin Remodeler |
| YEATS2 | Histone chaperone | 26153137;24240475 | Histone Modifier |
| YEATS4 | Histone modification write cofactor(Histone acetylation) | 26153137;24240475 | Histone Modifier |
| YWHAB | Histone modification erase cofactor(Histone acetylation) | 26153137 | Histone Modifier |
| YWHAE | Histone modification erase cofactor(Histone acetylation) | 26153137 | Histone Modifier |
| YWHAZ | Histone modification read | 26153137 | Histone Modifier |
| YY1 | Chromatin remodelling cofactor, TF(TF repressor);Also interacts with PRC2 and is required for EZH2-mediated H3K27me3 | 26153137;24063517;24063517;22196736;22196736;22196736 | Chromatin Remodeler |
| ZBTB16 | Histone modification erase cofactor(Histone acetylation) | 26153137 | Histone Modifier |
| ZBTB24 |  | 24240475 |  |
| ZBTB33 | Histone modification write cofactor, Histone modification erase cofactor, TF(Histone acetylation, Histone methylation, TF repressor);DNA modification(DNA methylation reader) | 26153137;26169266 | DNA Methylator,Histone Modifier |
| ZBTB38 | DNA modification(DNA methylation reader) | 26169266 | DNA Methylator |
| ZBTB4 | DNA modification(DNA methylation reader) | 26169266 | DNA Methylator |
| ZBTB7C | Histone modification cofactor | 26153137 | Histone Modifier |
| ZCWPW1 | Histone modification read | 26153137;24240475 | Histone Modifier |
| ZCWPW2 |  | 24240475 |  |
| ZFAT |  | 24240475 |  |
| ZFP57 | TF(TF repressor) | 26153137 |  |
| ZGPAT | TF(TF repressor) | 26153137 |  |
| ZHX1 | Chromatin remodelling, Histone modification write cofactor, Histone modification write cofactor(Histone acetylation, Histone methylation) | 26153137;24240475 | Histone Modifier,Chromatin Remodeler |
| ZHX2 |  | 24240475 |  |
| ZHX3 |  | 24240475 |  |
| ZMYM1 | Histone Modification(Acetylation, methylation and phosphorylation Reader) | 26169266 | Histone Modifier |
| ZMYM2 | Histone modification erase cofactor, TF(Histone acetylation);Histone Modification(Acetylation, methylation and phosphorylation Reader) | 26153137;26169266 | Histone Modifier |
| ZMYM3 | Histone modification erase cofactor(Histone acetylation);Histone Modification(Acetylation, methylation and phosphorylation Reader) | 26153137;26169266 | Histone Modifier |
| ZMYM4 | Histone Modification(Acetylation, methylation and phosphorylation Reader) | 26169266 | Histone Modifier |
| ZMYM5 | Histone Modification(Acetylation, methylation and phosphorylation Reader) | 26169266 | Histone Modifier |
| ZMYM6 | Histone Modification(Acetylation, methylation and phosphorylation Reader) | 26169266 | Histone Modifier |
| ZMYND11 | Histone modification read(Histone methylation) | 26153137;24240475 | Histone Modifier |
| ZMYND8 | Histone modification erase cofactor(Histone acetylation);Histone Modification(Acetylation, methylation and phosphorylation Reader) | 24240475;26153137;26169266;24240475 | Histone Modifier |
| ZNF217 | Histone modification erase cofactor, TF(Histone acetylation, TF repressor) | 26153137 | Histone Modifier |
| ZNF516 | Histone modification erase cofactor, TF(Histone acetylation, TF repressor) | 26153137 | Histone Modifier |
| ZNF532 | Histone modification erase cofactor(Histone acetylation) | 26153137 | Histone Modifier |
| ZNF541 | Chromatin remodelling | 26153137 | Chromatin Remodeler |
| ZNF592 | Histone modification erase cofactor(Histone acetylation) | 26153137 | Histone Modifier |
| ZNF687 | Histone modification erase cofactor(Histone acetylation) | 26153137 | Histone Modifier |
| ZNF711 | Histone modification erase cofactor(Histone acetylation) | 26153137 | Histone Modifier |
| ZNHIT1 | Chromatin remodelling cofactor, Histone modification erase cofactor( Histone acetylation) | 26153137 | Histone Modifier,Chromatin Remodeler |
| ZRANB3 | Chromatin remodelling, Histone modification read cofactor( Histone methylation) | 26153137;24240475 | Histone Modifier,Chromatin Remodeler |
| ZZZ3 | Histone modification read | 26153137 | Histone Modifier |
