# Supplementary material for: Characterization of molecular subtypes based on chromatin regulators and identification of the role of NPAS2 in lung adenocarcinoma
Source: Clin Epigenetics. 2023 Apr 29;15:72. doi: 10.1186/s13148-023-01486-w (PMC10149025; doi:10.1186/s13148-023-01486-w)
Supplement: Supplementary file 2 — Additional file 2: Table S2. Clinical characteristics of LUAD samples. [file 13148_2023_1486_MOESM2_ESM.docx]

**Supplementary table 1** Clinical characteristics of LUAD samples

| Variables | Total | TCGA | GSE37745 | GSE31210 |
| --- | --- | --- | --- | --- |
| Age (years) |  |  |  |  |
| <=65 | 457(57.12%) | 225(47.97%) | 56(53.33%) | 176(77.88%) |
| >65 | 343(42.88%) | 244(52.03%) | 49(46.67%) | 50(22.12%) |
| Gender |  |  |  |  |
| Female | 433(54.12%) | 252(53.73%) | 60(57.14%) | 121(53.54%) |
| Male | 367(45.88%) | 217(46.27%) | 45(42.86%) | 105(46.46%) |
| Stage |  |  |  |  |
| I | 492(61.5%) | 254(54.16%) | 70(66.67%) | 168(74.34%) |
| II | 190(23.75%) | 113(24.09%) | 19(18.1%) | 58(25.66%) |
| III | 89(11.12%) | 77(16.42%) | 12(11.43%) | 0(0%) |
| IV | 29(3.62%) | 25(5.33%) | 4(3.81%) | 0(0%) |
