# Supplementary figures and images for: Characterization of molecular subtypes based on chromatin regulators and identification of the role of NPAS2 in lung adenocarcinoma
Source: Clin Epigenetics. 2023 Apr 29;15:72. doi: 10.1186/s13148-023-01486-w (PMC10149025; doi:10.1186/s13148-023-01486-w)

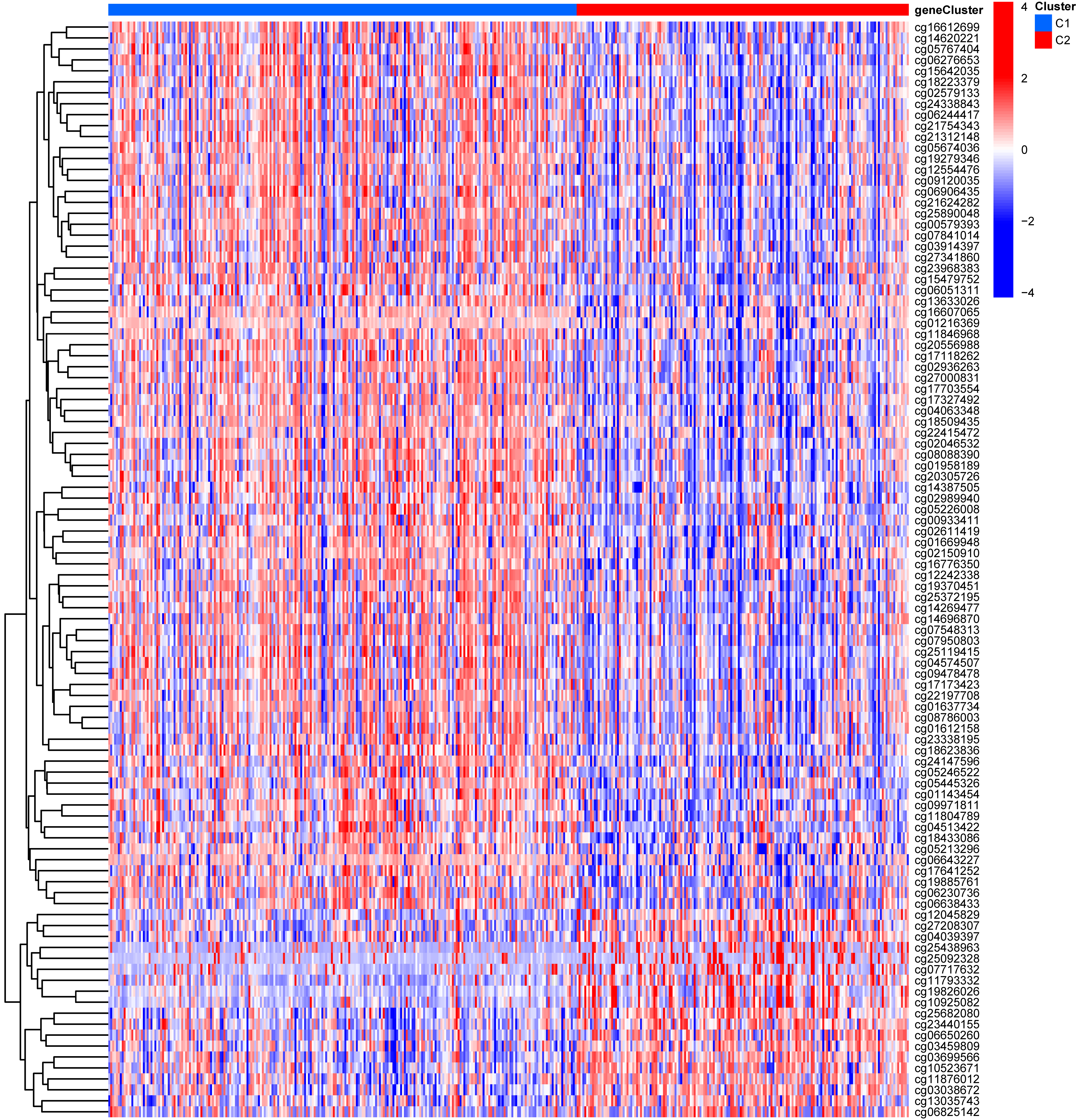

Supplement: Supplementary file 3 — Additional file 3: Fig. S1. Differentially methylated sites between Cluster C1 and C2. [file 13148_2023_1486_MOESM3_ESM.tif]

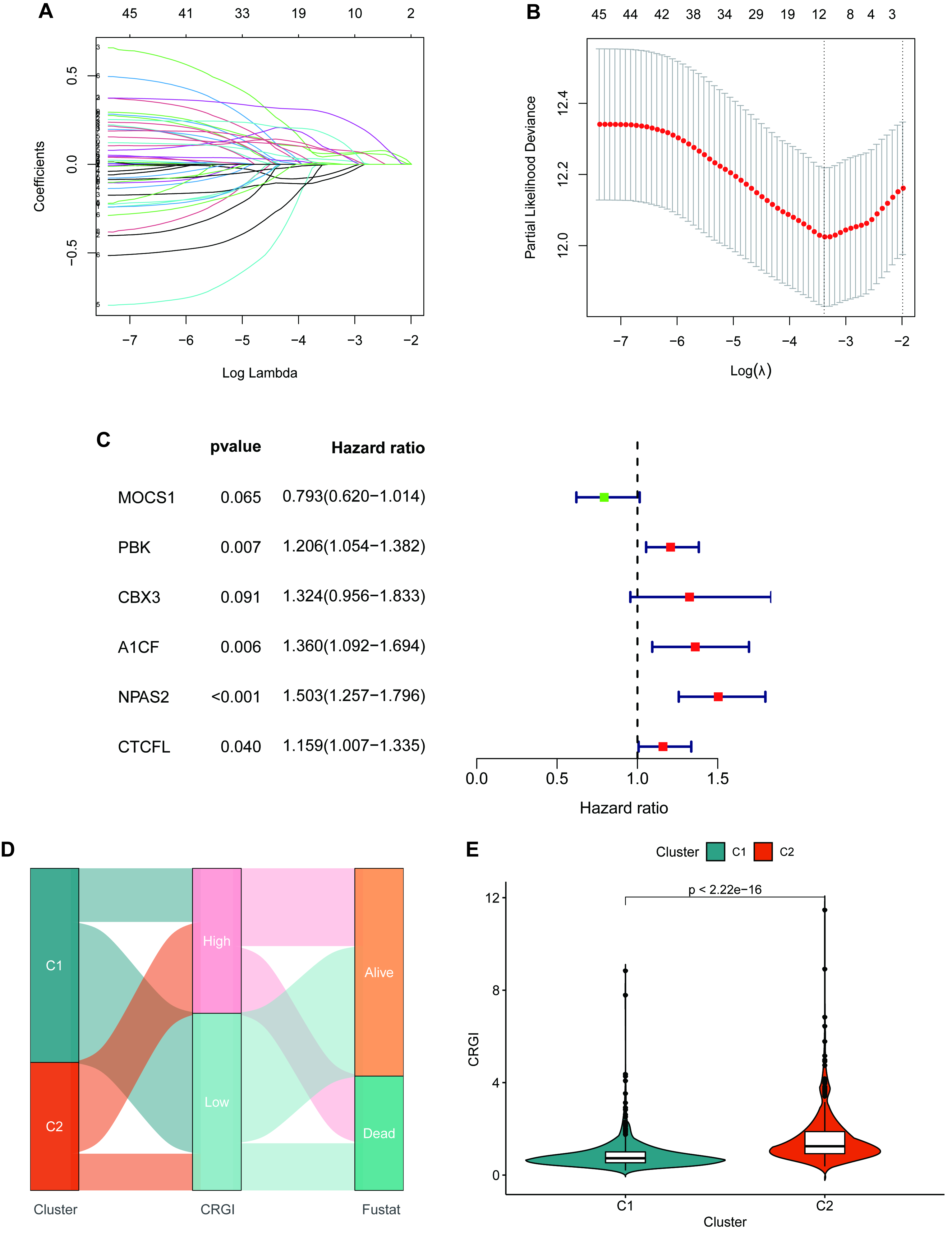

Supplement: Supplementary file 4 — Additional file 4: Fig. S2. Construction of a prognostic CR-related signature. (A–B) Variable selection in LASSO Cox regression analysis. (C) CRs selected in multivariate Cox regression analysis. (D) Correlation of CRGI with subtypes and survival status. (E) Difference in CRGI between the two subtypes. [file 13148_2023_1486_MOESM4_ESM.tif]

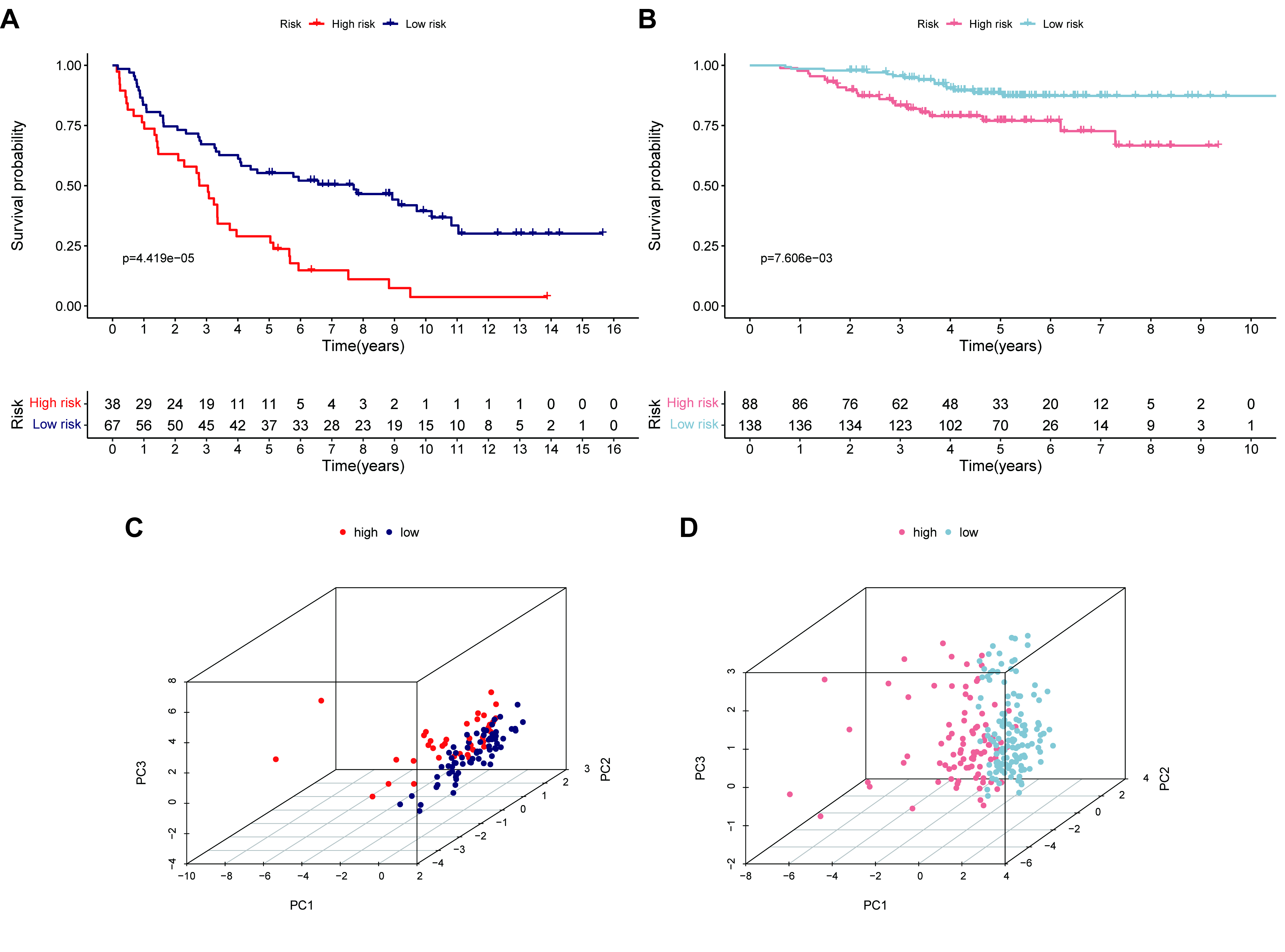

Supplement: Supplementary file 6 — Additional file 6: Fig. S3. Prognostic performance of CRGI in GEO datasets. (A) Survival difference between the high- and low-CRGI groups in GSE37745. (B) Survival difference between the high- and low-CRGI groups in GSE31210. (C) PCA plot showing distribution of the high- and low-CRGI groups in GSE37745. (D) PCA plot showing distribution of the high- and low-CRGI groups in GSE31210. [file 13148_2023_1486_MOESM6_ESM.tif]

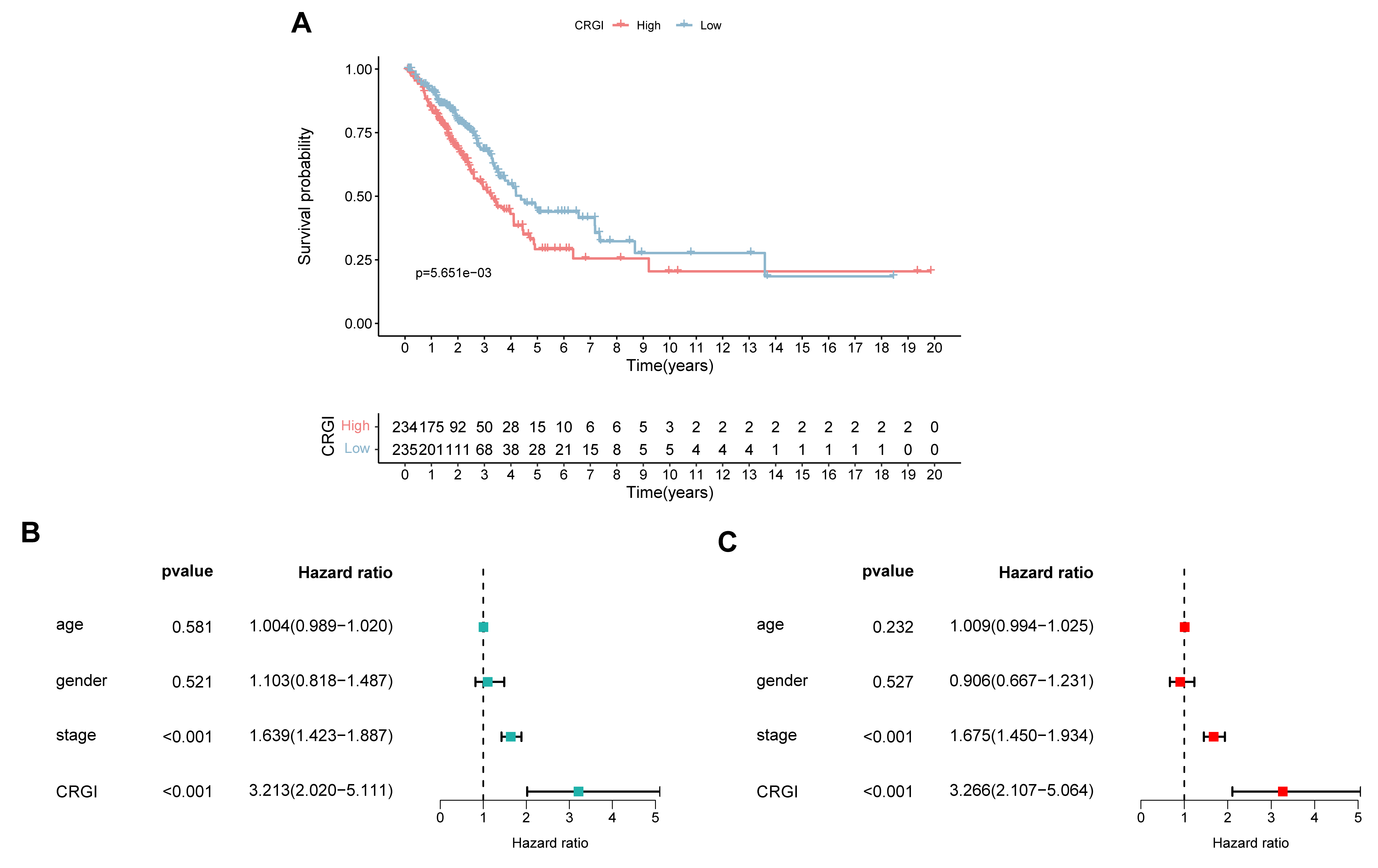

Supplement: Supplementary file 7 — Additional file 7: Fig. S4. Prognostic performance of CRGI after removal of cell cycle-related CRs. (A) Survival difference between the high- and low-CRGI groups after removal of cell cycle-related CRs. (C) Univariate Cox regression of CRGI and clinicopathological variables after removal of cell cycle-related CRs. (D) Multivariate Cox regression of CRGI and clinicopathological variables after removal of cell cycle-related CRs. [file 13148_2023_1486_MOESM7_ESM.tif]

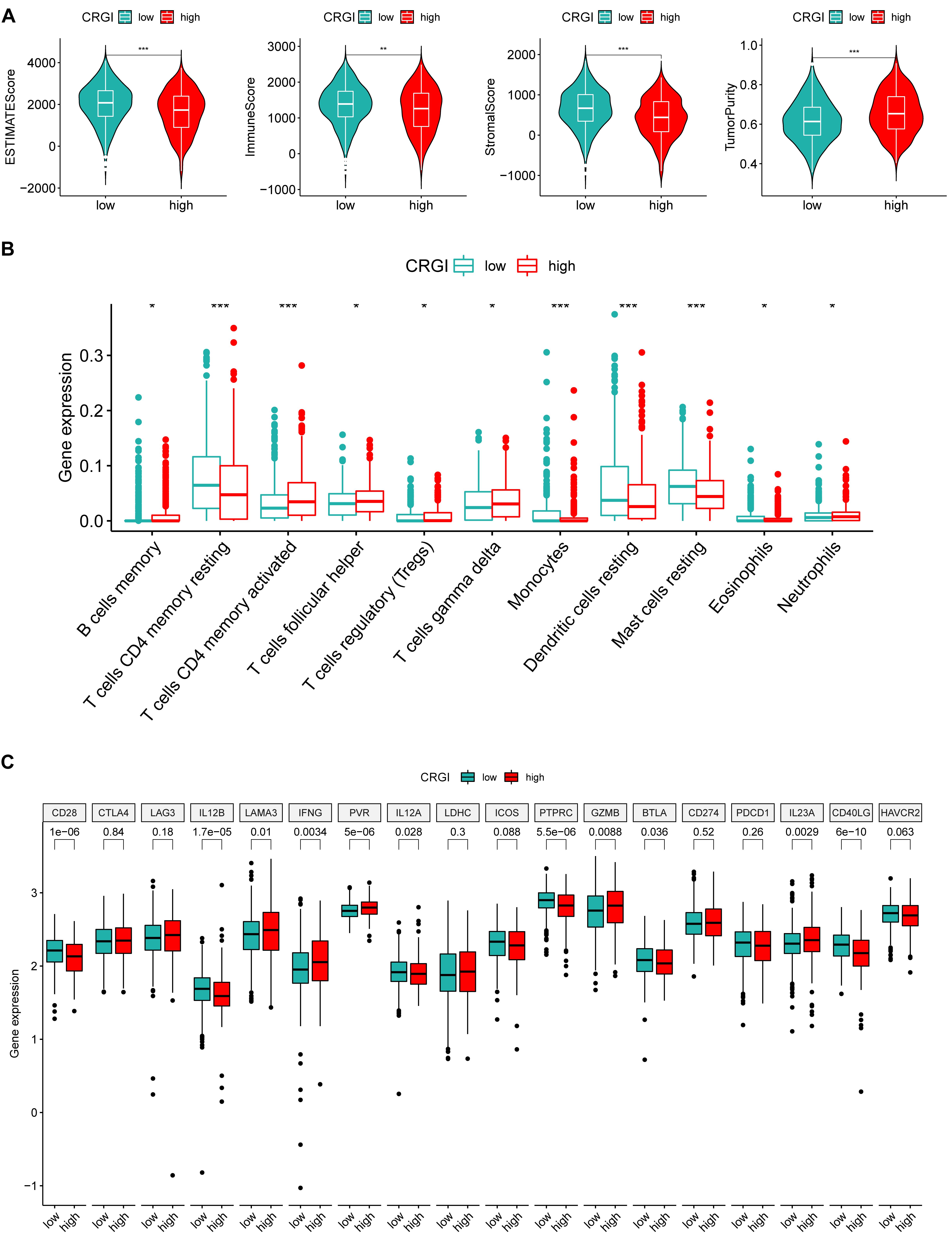

Supplement: Supplementary file 8 — Additional file 8: Fig. S5. Correlation between CRGI and TME after removal of cell cycle-related CRs. (A) Differences in ESTIMATE score, tumor purity, immune score, and stromal score between high- and low-CRGI groups after removal of cell cycle-related CRs. (B) Correlation between CRGI and infiltration of immune cells after removal of cell cycle-related CRs. (C) Differences in expression of immune checkpoint between high- and low-CRGI groups after removal of cell cycle-related CRs. [file 13148_2023_1486_MOESM8_ESM.tif]

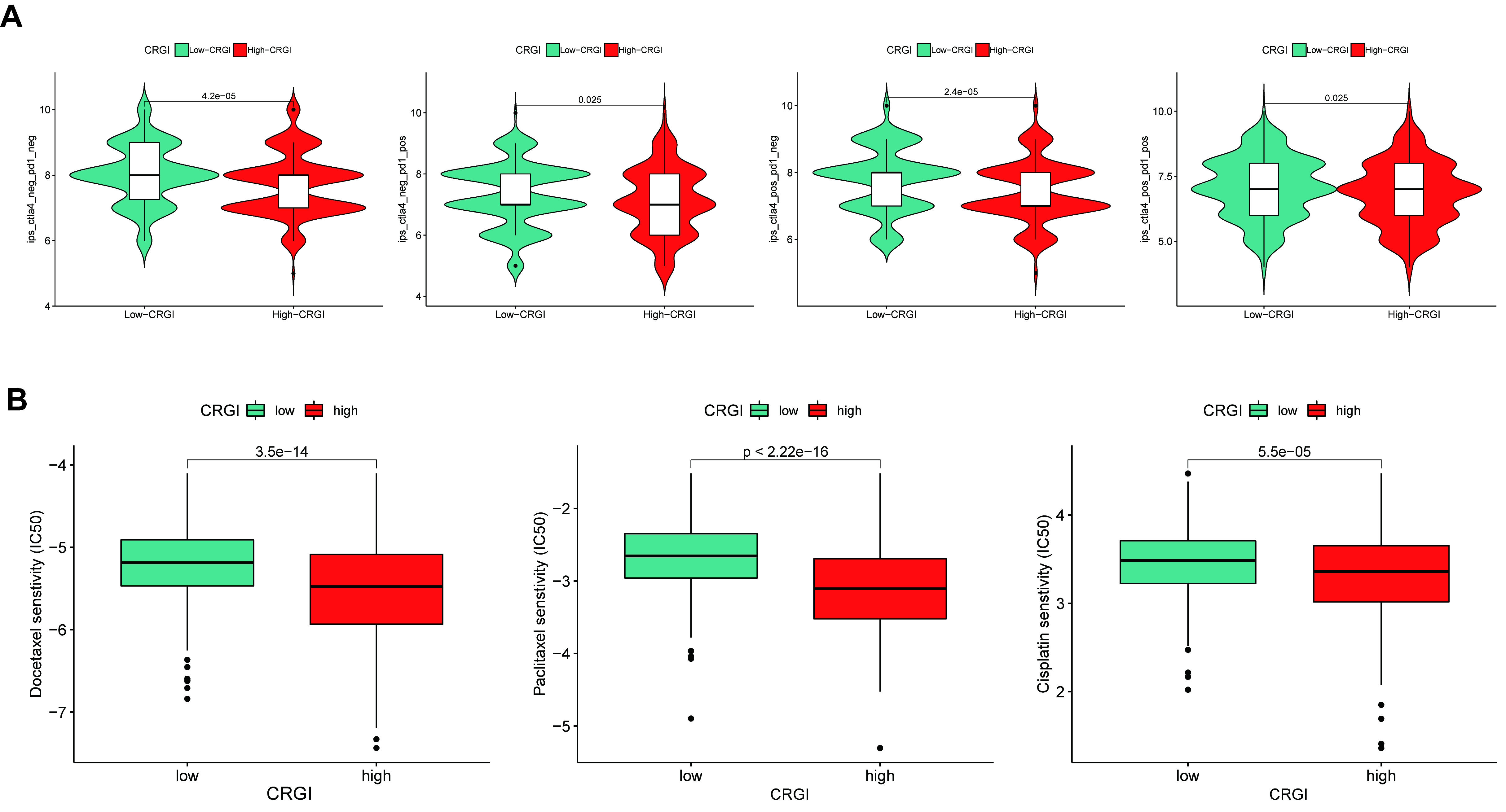

Supplement: Supplementary file 9 — Additional file 9: Fig. S6. Relationship between CRGI and response to treatment. (A) Differences in IPS between the high- and low-CRGI groups. (A) Differences in IC50 values of chemotherapeutic agents between the high- and low-CRGI groups. [file 13148_2023_1486_MOESM9_ESM.tif]
