# Supplementary material for: Characterization of molecular subtypes based on chromatin regulators and identification of the role of NPAS2 in lung adenocarcinoma
Source: Clin Epigenetics. 2023 Apr 29;15:72. doi: 10.1186/s13148-023-01486-w (PMC10149025; doi:10.1186/s13148-023-01486-w)
Supplement: Supplementary file 5 — Additional file 5: Table S3. The results of multivariate Cox regression analysis [file 13148_2023_1486_MOESM5_ESM.docx]

**Supplementary table 2** The results of multivariate Cox regression analysis

| id | coef | HR | 95%CI | P value |
| --- | --- | --- | --- | --- |
| MOCS1 | -0.2317 | 0.7932 | 0.6203-1.0142 | 0.0646 |
| PBK | 0.1877 | 1.2064 | 1.0535-1.3815 | 0.0066 |
| CBX3 | 0.2807 | 1.3241 | 0.9563-1.8334 | 0.0909 |
| A1CF | 0.3075 | 1.3601 | 1.0917-1.6944 | 0.0061 |
| NPAS2 | 0.4073 | 1.5027 | 1.2574-1.7959 | 0.0000 |
| CTCFL | 0.1476 | 1.1591 | 1.0065-1.3347 | 0.0403 |

CI = confidence interval; HR = hazard ratio
